# A haplotype-resolved, *de novo* genome assembly for the wood tiger moth (*Arctia plantaginis*) through trio binning

**DOI:** 10.1093/gigascience/giaa088

**Published:** 2020-08-18

**Authors:** Eugenie C Yen, Shane A McCarthy, Juan A Galarza, Tomas N Generalovic, Sarah Pelan, Petr Nguyen, Joana I Meier, Ian A Warren, Johanna Mappes, Richard Durbin, Chris D Jiggins

**Affiliations:** Department of Zoology, University of Cambridge, Downing Street, Cambridge CB2 3EJ, UK; Department of Genetics, University of Cambridge, Downing Street, Cambridge CB2 3EH, UK; Wellcome Sanger Institute, Wellcome Trust Genome Campus, Hinxton, Saffron Walden CB10 1SA, UK; Department of Biological and Environmental Science, University of Jyväskylä, FI-40014 Jyväskylä, Finland; Department of Zoology, University of Cambridge, Downing Street, Cambridge CB2 3EJ, UK; Wellcome Sanger Institute, Wellcome Trust Genome Campus, Hinxton, Saffron Walden CB10 1SA, UK; Biology Centre of the Czech Academy of Sciences, Institute of Entomology, Branišovská 1160/31, 370 05 České Budějovice, Czech Republic; University of South Bohemia, Faculty of Science, Branišovská 1645/31A, 370 05 České Budějovice, Czech Republic; Department of Zoology, University of Cambridge, Downing Street, Cambridge CB2 3EJ, UK; St John's College, University of Cambridge, St John's Street, Cambridge CB2 1TP, UK; Department of Zoology, University of Cambridge, Downing Street, Cambridge CB2 3EJ, UK; Department of Biological and Environmental Science, University of Jyväskylä, FI-40014 Jyväskylä, Finland; Department of Genetics, University of Cambridge, Downing Street, Cambridge CB2 3EH, UK; Wellcome Sanger Institute, Wellcome Trust Genome Campus, Hinxton, Saffron Walden CB10 1SA, UK; Department of Zoology, University of Cambridge, Downing Street, Cambridge CB2 3EJ, UK; St John's College, University of Cambridge, St John's Street, Cambridge CB2 1TP, UK

**Keywords:** wood tiger moth; *Arctia plantaginis*, Lepidoptera, genome assembly, trio binning, annotation, population genomics

## Abstract

**Background:**

Diploid genome assembly is typically impeded by heterozygosity because it introduces errors when haplotypes are collapsed into a consensus sequence. Trio binning offers an innovative solution that exploits heterozygosity for assembly. Short, parental reads are used to assign parental origin to long reads from their F1 offspring before assembly, enabling complete haplotype resolution. Trio binning could therefore provide an effective strategy for assembling highly heterozygous genomes, which are traditionally problematic, such as insect genomes. This includes the wood tiger moth (*Arctia plantaginis*), which is an evolutionary study system for warning colour polymorphism.

**Findings:**

We produced a high-quality, haplotype-resolved assembly for *Arctia plantaginis* through trio binning. We sequenced a same-species family (F1 heterozygosity ~1.9%) and used parental Illumina reads to bin 99.98% of offspring Pacific Biosciences reads by parental origin, before assembling each haplotype separately and scaffolding with 10X linked reads. Both assemblies are contiguous (mean scaffold N50: 8.2 Mb) and complete (mean BUSCO completeness: 97.3%), with annotations and 31 chromosomes identified through karyotyping. We used the assembly to analyse genome-wide population structure and relationships between 40 wild resequenced individuals from 5 populations across Europe, revealing the Georgian population as the most genetically differentiated with the lowest genetic diversity.

**Conclusions:**

We present the first invertebrate genome to be assembled via trio binning. This assembly is one of the highest quality genomes available for Lepidoptera, supporting trio binning as a potent strategy for assembling heterozygous genomes. Using our assembly, we provide genomic insights into the geographic population structure of *A. plantaginis*.

## Data Description

### Background

The ongoing explosion in *de novo* reference genome assembly for non-model organisms has been facilitated by the combination of advancing technologies and decreasing costs of next-generation sequencing [1]. Long-read sequencing technologies further revolutionized the quality of assembly achievable, with incorporation of long reads that can span common repetitive regions leading to radical improvements in contiguity [2]. However, heterozygosity still presents a major challenge to *de novo* assembly of diploid genomes. Most current technologies attempt to collapse parental haplotypes into a composite, haploid sequence, introducing erroneous duplications through mis-assembly of heterozygous sites as separate genomic regions. This problem is exacerbated in highly heterozygous genomes, resulting in fragmented and inflated assemblies that impede downstream analyses [3, 4]. Furthermore, a consensus sequence does not represent either true, parental haplotype, leading to loss of haplotype-specific information such as allelic and structural variants [5]. Whilst reducing heterozygosity by inbreeding has been a frequent approach, rearing inbred lines is unfeasible and highly time consuming for many non-model systems, and resulting genomes may no longer be representative of wild populations.

Trio binning is an innovative, new approach that takes advantage of heterozygosity instead of trying to remove it [6]. In this method, a family trio is sequenced with short reads for both parents and long reads for an F1 offspring. Parent-specific *k*-mer markers are then identified from the parental reads and used to assign offspring reads into maternal and paternal bins, before assembling each parental haploid genome separately [6]. The ability of trio binning to accurately distinguish parental haplotypes increases at greater heterozygosity, with high-quality, *de novo* assemblies achieved for bovid genomes by crossing different breeds [6] and species [7] to maximize heterozygosity. Therefore, trio binning has the potential to overcome current difficulties faced by highly heterozygous genomes, which have typically evaded high-quality assembly through conventional methods.

We utilized trio binning to assemble a high-quality, haplotype-resolved reference genome for the wood tiger moth (*Arctia plantaginis*, NCBI:txid874455; formerly *Parasemia plantaginis* [8]). At the time of writing, this represents the first trio-binned assembly available for an invertebrate animal species, diversifying the organisms for which published trio-binned assemblies exist beyond bovids [6, 7], zebra finches [9], humans [6, 9, 10], *Arabidopsis thaliana* [6], and additional trio-binned assemblies available for 8 other vertebrate species on the Vertebrate Genomes Project GenomeArk database [11]. Using a family trio with same-species *A. plantaginis* parents, 99.98% of offspring reads were successfully binned into parental haplotypes. This was facilitated by the high heterozygosity of the *A. plantaginis* genome; heterozygosity of the F1 offspring was estimated to be ~1.9%, exceeding levels obtained in all other published trio-binned assemblies through same-species crosses [6, 9, 10] and a yak-cow hybrid cross [7]. Both resulting haploid assemblies are highly contiguous and complete, strongly supporting trio binning as an effective strategy for *de novo* assembly of heterozygous genomes.

**Figure 1:**
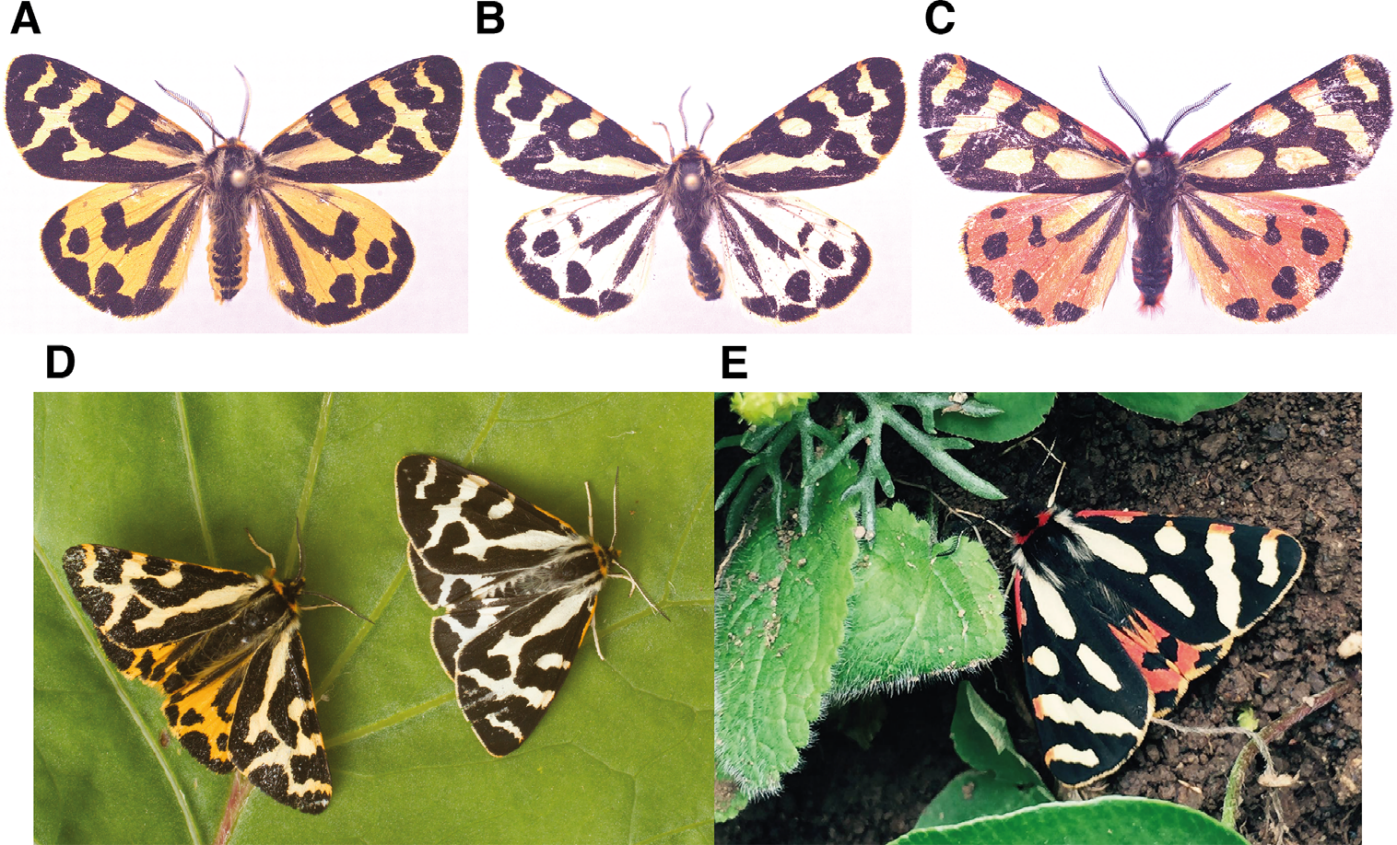
Discrete colour morphs of *Arctia plantaginis* males. Whilst forewings remain white, hindwings are polymorphic with variable black patterns, existing as discrete (**A)** yellow, (**B)** white, and (**C)** red morphs, which can only be found in the Caucasus region. **(A–C)** Pinned dead morphs. **(D,E)** Examples of morphs in the wild. Photos: Johanna Mappes and Ossi Nokelainen.

The presented *A. plantaginis* assembly will also provide an important contribution to the growing collection of lepidopteran reference genomes [12]. Comparative phylogenomic studies will benefit from the addition of *A. plantaginis* to the phylogenomic dataset [13, 14], being the first species to be sequenced within the Erebidae family [8, 15] and the first fully haplotype-resolved genome available for Lepidoptera. *A. plantaginis* itself is an important evolutionary study system, being a moth species that uses aposematic hindwing colouration to warn avian predators of its unpalatability [16]. Whilst female hindwing colouration varies continuously from orange to red, male hindwings exhibit a discrete colour polymorphism maintained within populations (Fig. 1), varying in frequency from yellow-white in Europe and Siberia and yellow-red in the Caucasus to black-white in North America and Northern Asia [17, 18]. Hence, *A. plantaginis* provides a natural system to study the evolutionary forces that promote phenotypic diversification on local and global scales, for which availability of a high-quality, haplotype-resolved and annotated reference genome will now transform genetic research.

## Materials and Methods

### Cross preparation and sequencing

To obtain an *A. plantaginis* family trio, selection lines for yellow and white male morphs were created from Finnish populations at the University of Jyväskylä over 3 consecutive generations. Larvae were fed with wild dandelion (*Taraxacum* spp.) and reared under natural light conditions, with an average temperature of 25°C during the day and 15–20°C at night until pupations. A father from the white selection line and mother from the yellow selection line were crossed, then collected and dry-frozen along with their F1 pupae at −20°C in 1.5 mL-sterile Eppendorf tubes.

For short-read sequencing of the father (sample ID: CAM015099; ENA accession No.: ERS4285278) and mother (sample ID: CAM015100; ENA accession No.: ERS4285279), DNA was extracted from adult thoraces using a Qiagen DNeasy Blood & Tissue Kit (Qiagen, Hilden, Germany) following the manufacturer's protocol, then library preparation and sequencing was performed by Novogene (Hong Kong, China). Illumina NEBNext (New England Biolabs, Ipswich, Massachusetts, USA) libraries were constructed with an insert size of 350 bp, following the manufacturer's protocol, and sequenced with 150-bp paired-end reads on an Illumina NovaSeq 6000 platform (Illumina NovaSeq 6000 Sequencing System, RRID:SCR_016387) (Illumina, San Diego, California, USA).

For long-read sequencing of a single F1 pupal offspring (Sample ID: CAM015101; ENA accession No.: ERS4285595), high–molecular weight DNA was extracted from the entire body of an F1 pupa using a Qiagen Blood & Culture DNA Midi Kit (Qiagen, Hilden, Germany) following the manufacturer's protocol, then library preparation and sequencing was performed by the Wellcome Sanger Institute (Cambridge, UK). A SMRTbell CLR (continuous long reads) sequencing library was constructed following the manufacturer's protocol and sequenced on 5 SMRT (single-molecule real-time) cells within a PacBio Sequel System (PacBio Sequel System, RRID:SCR_017989) (Pacific Biosciences, Menlo Park, California, USA) using version 3.0 chemistry and 10-hour runs. This generated 3,474,690 subreads, with a subread N50 of 18.8 kb and total of 39,471,717,610 bp. From the same sample, a 10X Genomics Chromium linked-read sequencing library (10X Genomics, Pleasanton, California, USA) was also prepared following the manufacturer's protocol, and sequenced with 150-bp paired-end reads on an Illumina HiSeq X Ten platform (Illumina HiSeq X Ten, RRID:SCR_016385) (Illumina, San Diego, California, USA). This generated 625,914,906 reads, and after mapping to the assembly described below, we estimate a barcoded molecule length of ~43 kb.

### Trio binning genome assembly

Canu version 1.8 (Canu, RRID:SCR_015880) [19] was used to bin *A. plantaginis* F1 offspring Pacific Biosciences (PacBio) subreads into those matching the paternal and maternal haplotypes defined by *k*-mers specific to the maternal and paternal Illumina data (Supplementary Fig. S1). This resulted in 1,662,000 subreads assigned to the paternal haplotype, 1,529,779 subreads assigned to the maternal haplotype, and 2,445 (0.07%) subreads unassigned. Using only the assigned reads, the haplotype-binned reads were assembled separately using wtdbg2 version 2.3 (wtdbg2, RRID:SCR_017225) [20], with the “-xsq” pre-set option for PacBio Sequel data and an estimated genome size of 550 Mb. The assemblies were polished using Arrow version 2.3.3 [21] and the haplotype-binned PacBio reads. The 10X linked-reads were then used to scaffold each assembly using scaff10x [22], followed by another round of Arrow polishing on the scaffolds. To polish further with the 10X linked-read Illumina data, we first concatenated the 2 scaffolded assemblies, mapped the 10X Illumina data with Long Ranger version 2.2.0 [23] longranger align, called variants with freebayes version 1.3.1 [24], then applied homozygous non-reference edits to the assembly using bcftools consensus [25]. The assembly was then split back into paternal and maternal components, giving separate paternal haplotype (iArcPla.TrioW) and maternal haplotype (iArcPla.TrioY) assemblies.

Assembly contaminants were identified and removed by checking the assemblies against vector/adapter sequences [26], common contaminants in eukaryotes [27] and mitochondrial sequences [28]. The assemblies were also checked against all chromosome-level genome sequences for other organisms from the RefSeq database version 85 [29]. This identified 2 scaffolds with mouse contamination, which were subsequently removed. The assemblies were further manually assessed and corrected using gEVAL [30] with the available PacBio and 10X data. This process involved locating regions of zero or extreme PacBio read coverage and missed or mis-joins indicated by the 10X data, then evaluating the flagged discordances and correcting them where possible, which were typically missed joins, mis-joins, and false duplications.

KAT version 2.4.2 [31] was used to compare *k*-mers from the 10X Illumina data to *k*-mers in each of the haplotype-resolved assemblies, and in the combined diploid assembly representing both haplotypes. For this analysis we used parameter K = 21, which clearly identified error, haploid, and diploid peaks for our dataset. Phasing of the assembled contigs and scaffolds was visualized using the parental *k*-mer databases produced by Canu [32]. To provide an estimate of assembly consensus accuracy, a quality value (QV) was computed for each assembly using Merqury version 1.0 [33]. Haploid genome size, heterozygosity, and repeat fraction of the F1 offspring were estimated using GenomeScope (GenomeScope, RRID:SCR_017014) [34] and *k*-mers derived from the 10X Illumina data. Assemblytics [35] was used to detect structural variants (SVs) between the parental haplotypes. For this, a whole-genome alignment was performed between the haplotype assemblies using the Nucmer module of MUMmer version 3.23 (MUMmer, RRID:SCR_018171) [36] with Assemblytics recommended options.

### Comparative quality assessment

To assess the quality of each parental haplotype of the *A. plantaginis* trio-binned assembly, standard contiguity metrics were computed, and assembly completeness was evaluated by calculating BUSCO scores using BUSCO version 3.0.2 (BUSCO, RRID:SCR_015008), comparing against the “insecta_odb9” database of 1,658 Insecta BUSCO genes with default Augustus (Augustus, RRID:SCR_008417) parameters [37]. A quality comparison was conducted by comparing unscaffolded, Arrow-polished versions of the trio-binned assemblies against an unscaffolded, Arrow-polished assembly of unbinned data from the same F1 offspring (iArcPla.wtdbg2). Quality comparisons were also performed for the final, scaffolded trio-binned assemblies against a representative selection of published lepidopteran reference genomes, for which the latest versions of 7 Lepidoptera species were downloaded: *Bicyclus anynana* version 1.2 [38], *Danaus plexippus* version 3 [39], *Heliconius melpomene* version Hmel.2.5 [40], *Manduca sexta* version Msex_1.0 [41], and *Melitaea cinxia* version MelCinx1.0 [42] were downloaded from Lepbase version 4.0 [12], whilst *Bombyx mori* version Bomo_genome_assembly [43] was downloaded from SilkBase version 2.1 [44] and *Trichoplusia ni* version PPHH01.1 [45] was downloaded from RefSeq version 94 [46]. Cumulative scaffold plots were visualized in R version 3.5.1 [47] using the ggplot2 package version 3.1.1 (ggplot2, RRID:SCR_014601) [48].

### Genome annotation

Genome annotations were produced for each parental haplotype of the *A. plantaginis* trio-binned assembly using the BRAKER2 version 2.1.3 pipeline [49]. A *de novo* library of repetitive sequences was identified with both genomes using RepeatScout version 1.0.5 (RepeatScout, RRID:SCR_014653) [50]. Repetitive regions of the genomes were soft-masked using RepeatMasker version 4.0.9 (RepeatMasker, RRID:SCR_012954) [51], Tandem Repeats Finder version 4.00 [52], and the RMBlast version 2.6.0 sequence search engine [53] combined with the Dfam_Consensus-20170127 database [54]. Raw RNA-seq reads were obtained from Galarza et al. [55] under study accession No. PRJEB14172, which came from whole-body tissue of *A. plantaginis* larvae from 2 families reared under 2 heat treatments. Using cutadapt version 1.8.1 (cutadapt, RRID:SCR_011841) [56], RNA-seq reads were trimmed for adapter contamination and quality trimmed at both ends of each read using a quality value of 3 (-q 3,3). Quality control was performed before and after trimming with fastqc version 0.11.8 [57]. RNA-seq reads were mapped to each respective genome using STAR version 2.7.1 [58]. Arthropod proteins were obtained from OrthoDB [59] and aligned to the genomes using GenomeThreader version 1.7.0 [60]. BRAKER2's *ab initio* gene predictions were carried out using homologous protein and *de novo* RNA-seq evidence using Augustus version 3.3.2 [49] and GeneMark-ET version 4.38 [49]. Annotation completeness was assessed using BUSCO version 3.0.2 against the “insecta_odb9” database of 1,658 Insecta BUSCO genes with default Augustus parameters [37].

### Cytogenetic analysis

Spread chromosome preparations for cytogenetic analysis were produced from wing imaginal discs and gonads of third to fifth instar larvae, according to Šíchová et al. [61]. Female and male genomic DNA were extracted using the CTAB (hexadecyltrimethylammonium bromide) method, adapted from Winnepenninckx et al. [62]. These were used to generate probe and competitor DNA, respectively, for genomic *in situ* hybridization (GISH). Female genomic probe was labelled with Cy3-dUTP (cyanine 3-deoxyuridine triphosphate; Jena Bioscience, Jena, Germany) by nick translation, following Kato et al. [63] with a 3.5-hour incubation at 15°C. Male competitor DNA was fragmented with a 20-minute boil. GISH was performed following the protocol of Yoshido et al. [64]. For each slide, the hybridization cocktail contained 250 ng of female labelled probe, 2–3 μg of male competitor DNA, and 25 μg of salmon sperm DNA. Preparations were counterstained with 0.5 mg/mL DAPI (4′,6-diamidino-2-phenylindole; Sigma-Aldrich, St. Louis, Missouri, USA) in DABCO antifade (1,4-diazabicyclo[2.2.2]octane; Sigma-Aldrich, St. Louis, Missouri, USA). Results were observed in the Zeiss Axioplan 2 Microscope (Carl Zeiss, Oberkochen, Germany) and documented with an Olympus CCDMonochrome Camera XM10, with the cellSens 1.9 digital imaging software (Olympus Europa Holding, Hamburg, Germany). Images were pseudo-colored and superimposed in Adobe Photoshop CS3.

### Population genomic analysis

We implemented the novel *A. plantaginis* reference assembly to analyse patterns of population genomic variation between 40 wild, adult males sampled from the European portion of *A. plantaginis'* Holarctic species range [18]. Samples were collected by netting and pheromone traps from central Finnish (n = 10) and southern Finnish populations (n = 10) where yellow and white morphs exist in equal proportions, an Estonian population (n = 5) where white morphs are frequent compared to rare yellow morphs, a Scottish population (n = 10) where only yellow morphs exist, and a Georgian population (n = 5) where red morphs exist alongside yellow morphs (Fig. 5A). Exact sampling localities are available in Supplementary Table S1. Whole genomic DNA extraction and short-read sequencing was performed following the same method as described for short-read sequencing of parental genomes during trio binning assembly. ENA accession numbers for all resequenced samples are available in Supplementary Table S2.

Reads were mapped against the paternal iArcPla.TrioW assembly (chosen owing to higher assembly completeness; Table 2) using BWA-MEM version 7.17 [65] with default parameters, resulting in a mean sequencing coverage of 13× (Supplementary Table S2). Alignments were sorted with SAMtools version 1.9 (SAMtools, RRID:SCR_002105) [66] and PCR-duplicates were removed with Picard version 2.18.15 (Picard, RRID:SCR_006525) [67]. Variants were called for each sample using GATK HaplotypeCaller version 3.7 [68, 69], followed by joint genotyping across all samples using GATK version 4.1 GenotypeGVCFs [68, 69], with expected heterozygosity set to 0.01. The raw single-nucleotide polymorphism (SNP) call set was quality filtered by applying thresholds: quality by depth (QD > 2.0), root mean square mapping quality (MQ > 50.0), mapping quality rank sum test (MQRankSum > −12.5), read position rank sum test (ReadPosRankSum > −8.0), Fisher strand bias (FS < 60.0), and strand odds ratio (SOR < 3.0). Filters by depth (DP) of greater than half the mean (DP > 409x) and less than double the mean (DP < 1,636x were also applied. Linkage disequilibrium (LD) pruning was applied using the ldPruning.sh script [70] with an LD threshold of *r*^2^ < 0.01, in 50-kb windows shifting by 10 kb. This call set was further filtered for probability of heterozygosity excess *P*-value > 1 × 10^−5^ using VCFtools version 0.1.15 (VCFtools, RRID:SCR_001235) [71] to exclude potential paralogous regions, giving our analysis-ready call set.

An unrooted, maximum likelihood (ML) phylogenetic tree was constructed to evaluate phylogenomic relationships, using our analysis-ready call set, which was further reduced in size by subsampling every other SNP. The best-scoring ML tree was built in RAxML version 8.2.12 [72] with 100 rapid bootstrap replicates, using the GTRGAMMA model (generalized time-reversible substitution model and gamma model of rate heterogeneity) and Lewis ascertainment bias correction to account for the lack of monomorphic sites, then visualized in FigTree version 1.4.4 (FigTree, RRID:SCR_008515) [73]. A principal component analysis (PCA) was also conducted to evaluate genome-wide population structure. A minor-allele frequency filter of 0.05 was applied to our analysis-ready call set using VCFtools version 0.1.15 [71] to remove PCA-uninformative SNPs, then PCA was performed in R version 3.5.1 [47] using the SNPRelate package version 3.3 [74].

## Results and Discussion

### Trio binning genome assembly

**Figure 2:**
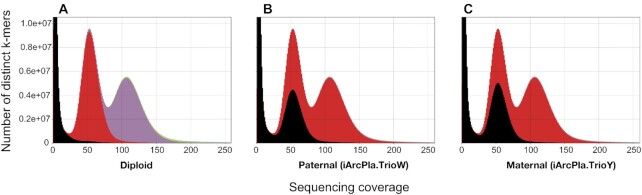
*k*-mer spectra plots for the *Arctia plantaginis* trio-binned genome assembly. Plots produced using KAT, showing the frequency of *k*-mers in an assembly vs the frequency of *k*-mers (i.e., sequencing coverage) in the raw 10X Illumina reads, for the **(A)** combined diploid assembly (paternal plus maternal), **(B)** paternal-only assembly (iArcPla.TrioW), and **(C)** maternal-only assembly (iArcPla.TrioY). Colours represent *k*-mer copy number in the assembly: black *k*-mers are not represented (0 copies), red *k*-mers are represented once (1 copy), purple *k*-mers are represented twice (2 copies), and green *k*-mers are represented thrice (3 copies). The first peak corresponds to *k-*mers present in the raw reads but missing from the assembly due to sequencing errors, the second peak corresponds to *k*-mers from heterozygous regions, and the third peak corresponds to *k*-mers from homozygous regions. These plots show a complete and well-separated assembly of both haplotypes in the F1 offspring diploid genome.

The *k*-mer spectra plots (Fig. 2) indicate a highly complete assembly of both parental haplotypes in the *A. plantaginis* diploid offspring genome. There is good separation between the parental haplotypes because each haploid assembly consists mostly of single-copy *k*-mers with low frequency of 2-copy *k*-mers, indicating a correctly haplotype-resolved assembly with low levels of artefactual duplication (Fig. 2B and C; Supplementary Fig. S2). This is also confirmed by the spectra plot for the combined diploid assembly (Fig. 2A), where homozygous regions consist mostly of 2-copy *k*-mers and heterozygous regions consist mostly of 1-copy *k*-mers, as expected from the presence of both complete, parental haplotypes and low artefactual duplication. Using Merqury [33], we estimated QV scores of Q34.7 for the paternal (iArcPla.TrioW) assembly and Q34.2 for the maternal (iArcPla.TrioY) assembly, indicating high (>99.9%) assembly accuracy.

Using GenomeScope [34], we estimated the F1 offspring haploid genome size to be 590 Mb with a repeat fraction of 27% and whole-genome heterozygosity of ~1.9% (Supplementary Fig. S3). This value was similar to our mean heterozygosity estimate of ~1.8% in a wild, Finnish population (Supplementary Table S4; method described in Supplementary Text S2), demonstrating that our reference assembly is representative of natural variation in a wild population. The slight discrepancy may be explained by the parents used for trio binning assembly being derived from different selection lines, leading to greater heterozygosity between the trio-binned parental haplotypes. Assemblytics [35] detected 32,203 SVs between the haplotype assemblies, affecting 51.6 Mb of the genome (Supplementary Table S5; Supplementary Fig. S4). Successful haplotype separation was facilitated by the high estimated heterozygosity (~1.9%) of the F1 offspring genome, as it has previously been discussed that higher heterozygosity makes trio binning easier [6]. Indeed, greater heterozygosity levels were obtained through our same-species *A. plantaginis* cross than obtained previously through same-species crosses for zebra finch (~1.6%) [9], *Arabidopsis* (~1.4%) [6], bovid (~0.9%) [6], and human (~0.1%) [6] trio-binned assemblies, as well as an inter-species yak (*Bos grunniens)* × cattle (*Bos taurus)* cross (~1.2%) [7].

### Genome annotation

We identified and masked 222,866,714 bp (41.04%) and 227,797,418 bp (42.80%) of repetitive regions in the iArcPla.TrioW and iArcPla.TrioY assemblies, respectively (Table 1). The BRAKER2 pipeline annotated a total of 19,899 protein-coding genes in the soft-masked iArcPla.TrioW genome with 98.00% BUSCO completeness, whilst 18,894 protein-coding genes were annotated in the soft-masked iArcPla.TrioY genome with 95.90% BUSCO completeness (Table 1).

**Table 1:** Genome annotation statistics for the *Arctia plantaginis* trio-binned assembly

Statistic	iArcPla.TrioW (paternal)	iArcPla.TrioY (maternal)
Total genome size (bp)	584,621,344	577,993,050
Repetitive sequences (bp)	239,949,688	247,356,128
Masked repeats (%)	41.04	42.80
Mapped RNA-seq reads (n)	599,065,138	590,780,528
Mapped RNA-seq reads (%)	366,732	94.13
Protein-coding genes (n)	19,899	18,894
Mean gene length (bp)	5,966	5,951
BUSCO Completeness (%; n: 1,658)	98.00	95.90
Repeat elements (n)		
Total	1,220,592	1,232,654
DNA Transposons	366,732	372,834
LTRs	127,169	139,770
LINEs	425,833	433,388
SINEs	43,022	71,790
Unclassified	257,836	214,872

Statistics generated using the BRAKER2 pipeline, for the paternal (iArcPla.TrioW) and maternal (iArcPla.TrioY) haplotype assemblies. LINE: long interspersed terminal repeat; LTR: long terminal repeat; SINE: short interspersed nuclear element.

### Comparative quality assessment

**Figure 3:**
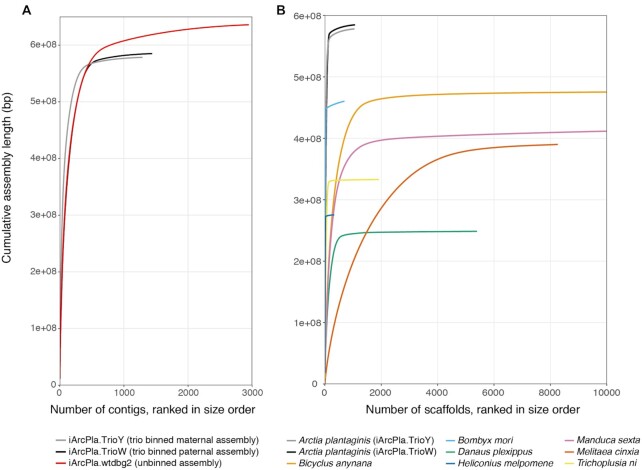
Cumulative scaffold plots visualize the high assembly contiguity of the trio-binned *Arctia plantaginis* genome. A highly contiguous assembly is represented by a near vertical line with a short horizontal tail of trailing tiny scaffolds. **(A)** Comparison of the unscaffolded *A. plantaginis* trio-binned assemblies iArcPla.TrioW (paternal haplotype) and iArcPla.TrioY (maternal haplotype) against the unscaffolded composite assembly using unbinned data from the same individual (iArcPla.wtdbg2). The much steeper curve and shorter horizontal tail for the trio-binned assemblies compared to the unbinned assembly shows that trio binning greatly improved contiguity. **(B)** Comparison of the *A. plantaginis* trio-binned assemblies against a representative selection of published lepidopteran genomes, shown up to the first 10,000 scaffolds. This comparison demonstrates that the *A. plantaginis* trio-binned assemblies are much more contiguous than most other lepidopteran genomes currently available.

The paternal (iArcPla.TrioW) assembly contains 1,069 scaffolds with N50 = 6.73 Mb and 98.1% complete BUSCOs, and the maternal (iArcPla.TrioY) assembly contains 1,050 scaffolds with N50 = 9.77 Mb and 96.4% complete BUSCOs (Table 2). Prior to scaffolding work with 10X data, both unscaffolded trio-binned assemblies are already more contiguous and complete than a composite haploid iArcPla.wtdbg2 assembly produced using unbinned data from the same individual (Table 2; Fig. 3A). This illustrates the quality improvement achieved by separating haplotypes before assembly, and further improvement of the trio-binned assemblies after scaffolding with 10X linked-reads (Table 2). The trio-binned assemblies are also less inflated than the unbinned assembly with halved duplicated BUSCOs (Table 2; Fig. 3A), suggesting a reduction in artefactual assembly duplication at heterozygous sites through read binning.

**Table 2:** Comparison of assembly contiguity and completeness between *Arctia plantaginis* and 7 publicly available lepidopteran assemblies

	Assembly contiguity					Assembly completeness (%)		
	Assembly size (Mb)	Total scaffolds/contigs	Longest scaffold/contig (Mb)	N50 (kb)	N50 count	Total complete BUSCOs	Single copy BUSCOs	Duplicated BUSCOs
*Arctia plantaginis* (binned: iArcPla.TrioW, scaffolded assembly)	585	1,069	21.5	6,730	24	98.1	96.9	1.2
*Arctia plantaginis* (binned: iArcPla.TrioY, scaffolded assembly)	578	1,050	24.4	9,770	18	96.4	95.3	1.1
*Arctia plantaginis* (binned: iArcPla.TrioW, unscaffolded assembly)	585	1,441	11.4	2,000	75	97.4	96.4	1.0
*Arctia plantaginis* (binned: iArcPla.TrioY, unscaffolded assembly)	578	1,290	23.8	4,016	37	95.1	94.1	1.0
*Arctia plantaginis* (unbinned: iArcPla.wtdbg2, unscaffolded assembly)	615	2,948	11.3	1,840	85	96.9	94.8	2.1
*Bicyclus anynana*	475	10,800	5.04	638.3	194	97.6	96.8	0.8
*Bombyx mori*	482	696	21.5	16,796	13	98.4	97.2	1.2
*Danaus plexippus*	249	5,397	6.24	715.6	101	98.0	96.0	2.0
*Heliconius melpomene*	275	332	18.1	14,308	9	97.7	96.7	1.0
*Manduca sexta*	419	20,871	3.25	664.0	169	96.7	93.9	2.8
*Melitaea cinxia*	390	8,261	0.668	119.3	970	83.0	82.9	0.1
*Trichoplusia ni*	333	1,916	8.93	4,648	27	97.4	96.6	0.8

Standard contiguity and BUSCO completeness metrics generated for each genome assembly, highlighting the high-quality *A. plantaginis* assembly achieved by trio binning. See Fig. 3 for assembly contiguity visualization via cumulative scaffold plots, and Supplementary Table S3 for the full BUSCO analysis summary.

The trio-binned *A. plantaginis* assemblies are of comparable quality to the best reference genomes available for Lepidoptera (Table 2; Fig. 3B). When compared to other published lepidopteran reference genomes, the quality of the *A. plantaginis* assemblies surpasses all but the best *Heliconius melpomene* [40] and *Bombyx mori* [43] assemblies (Table 2; Fig. 3B). As contiguity of the *H. melpomene* assembly was improved through pedigree linkage mapping and haplotypic sequence merging [40], whilst bacterial artificial chromosome and fosmid clones were used to close gaps in the *B. mori* assembly [43], it is impressive that trio binning has instantly propelled contiguity of the *A. plantaginis* genome to very near that of *H. melpomene* and *B. mori*, before incorporating information from additional technologies. Therefore, these comparisons strongly support trio binning as an effective strategy for *de novo* assembly of highly heterozygous genomes. Future scaffolding work has the potential to lead to a chromosomal-scale *A. plantaginis* assembly.

### Cytogenetic analysis

**Figure 4:**
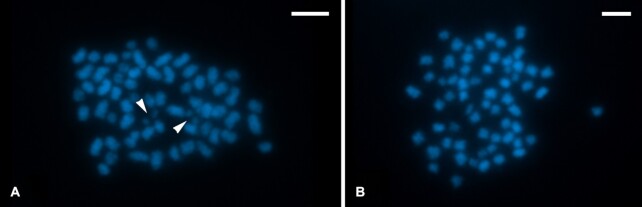
Cytogenetic analysis reveals 31 chromosomes in the *Arctia plantaginis* haploid genome. Chromosomes were counterstained with DAPI (blue). **(A)** Male mitotic metaphase consisted of 2n = 62 chromosomes. Note separated chromatids of the smallest chromosome pair (arrowheads). **(B)** Female mitotic complement consisted of 2n = 62 elements. Scale bar = 5 μm.

Mitotic nuclei prepared from wing imaginal discs of *A. plantaginis* larvae contained 2n = 62 chromosomes in both sexes (Fig. 4), in agreement with a previously reported modal chromosome number of arctiid moths [75], which is also the likely ancestral lepidopteran karyotype [42]. These insights will be helpful for future scaffolding work into a chromosomal-scale *A. plantaginis* reference assembly. Chromosomes decreased gradually in size, as is typical for lepidopteran karyotypes [76]. Owing to the holokinetic nature of lepidopteran chromosomes, separation of sister chromatids by parallel disjunction was observed in mitotic metaphases [77]. Notably, the 2 smallest chromosomes separated earlier compared to the other chromosomes (Fig. 4A), although this could be an artefact of the spreading technique used for chromosome preparation. The presence of a W chromosome was confirmed in female nuclei by genomic *in situ* hybridization (Supplementary Fig. S5; Supplementary Text S2).

### Population genomic variation across the European range

**Figure 5:**
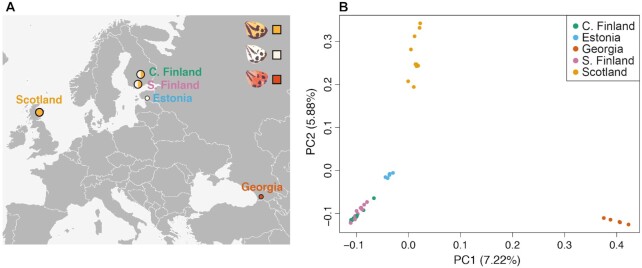
Sampling locations and population structure across *Arctia plantaginis'* European geographic range. **(A)** Sampling locations of 40 wild *A. plantaginis* males from the European portion of the Holarctic species range (see Supplementary Table S1 for exact sampling coordinates). Circle size represents sample size (central Finland: n = 10, Estonia: n = 5, Scotland: n = 10, southern Finland: n = 10, Georgia: n = 5), and circle colour indicates the proportion of each hindwing colour morph collected. **(B)** Genome-wide PCA (n = 40; 752,303 SNPs) with principal component 1 plotted against principal component 2, explaining 7.22% and 5.88% of total genetic variance, respectively.

**Figure 6:**
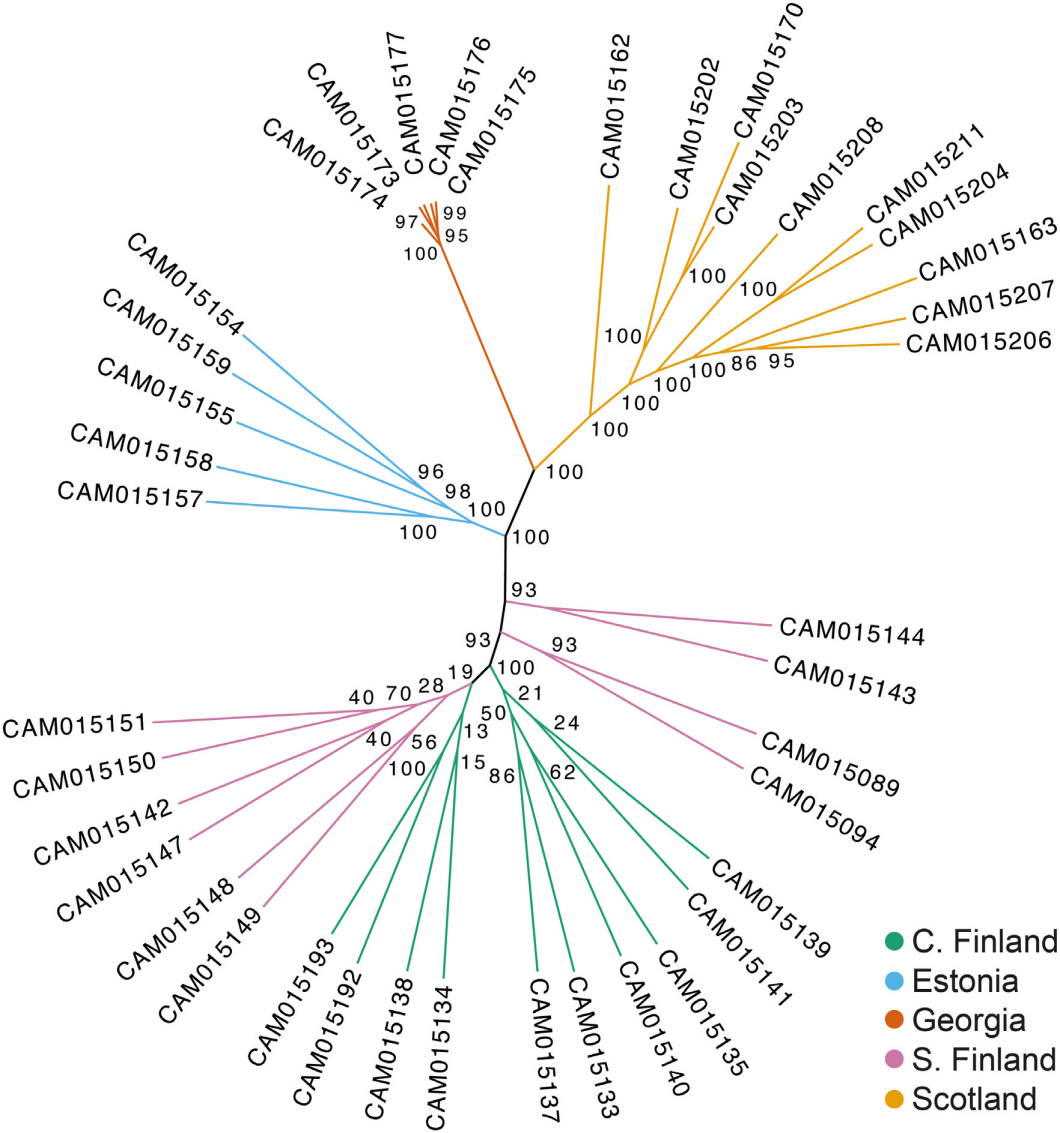
Maximum likelihood unrooted phylogeny of wild *Arctia plantaginis* males (n = 40) from the European geographic range. Tree constructed using RAxML with 100 rapid bootstraps, using 558,549 SNPs. Node labels indicate bootstrap support. See Fig. 5A for sampling locations.

As an empirical application of the *A. plantaginis* reference genome, we conducted a population resequencing analysis to describe genomic variation between 40 wild *A. plantaginis* males from 5 populations spread across Europe (Fig. 5A). PCA revealed clear population structuring with individuals clustering geographically by country of origin (Fig. 5B), in congruence with strongly supported phylogenomic groupings also by country of origin (Fig. 6). Central and southern Finnish individuals grouped into a single population as expected from their geographic proximity (Figs 5B and 6). The Finnish and Estonian populations clustered together away from the Scottish population along principal component (PC) 2 (Fig. 5B) and on the phylogenetic tree (Fig. 6), as would be predicted by effects of isolation by distance [78]. The Georgian population was highly genetically differentiated from all other sampled European populations, separating far along PC1 (Fig. 5B) and possessing a much longer inter-population branch in the ML tree (Fig. 6). Because the Georgian population has a distinctive genomic composition from the rest of the sampled distribution, this could support the hypothesis of incipient speciation in the Caucasus [18]. However, populations must be sampled in the large geographic gap between Georgia and the other populations in this preliminary analysis to determine whether genetic differentiation still persists when compared to nearby central European populations.

Internal branch lengths were strikingly shorter within the Georgian population, indicating much higher intra-population relatedness than in populations outside of Georgia (Fig. 6). This signal of low genetic variation within Georgia was unlikely caused by sampling relatives because individuals were collected from a large population. Whilst further sampling is required to confirm whether the signal persists across the Caucasus, this finding casts doubt on the hypothesis that the *A. plantaginis* species originated in the Caucasus, which is based on morphological parsimony [18]. If *A. plantaginis* spread from the Caucasus with a narrow founder population, as suggested in Hegna et al. [18], we would expect higher genetic diversity in the Caucasus compared to the other geographic regions. Similar patterns of strong genetic differentiation and low genetic diversity in the Caucasus and other European mountain ranges have been observed in the Holarctic butterfly *Boloria eunomia* [79], which likely retreated into refugia provided by warmer micro-habitats within European mountain ranges during particularly harsh glaciation periods. Perhaps a similar scenario occurred in *A. plantaginis*, with founders of the Caucasus population restricted during severe glacial conditions. The species origin of *A. plantaginis* therefore remains unknown and may be clarified by future inclusion of an *Arctia* outgroup to root the phylogenetic tree.

## Conclusions

By converting heterozygosity into an asset rather than a hindrance, trio binning provides an effective solution for *de novo* assembly of heterozygous regions, with our high-quality *A. plantaginis* reference genome paving the way for the use of trio binning to successfully assemble other highly heterozygous genomes. As the first trio-binned genome available for any invertebrate species, our *A. plantaginis* assembly adds support to trio binning as the best method for achieving fully haplotype-resolved, diploid genomes. Our assembly further highlights that trio binning can work well for a non-model system, provided a family trio can be obtained, which remains challenging for many non-model systems where it is difficult to obtain both parents and rear their offspring. Finally, the high-quality *A. plantaginis* reference assembly and annotation itself will contribute to Lepidoptera comparative phylogenomics by broadening taxonomic sampling into the Erebidae family, whilst facilitating genomic research on the *A. plantaginis* evolutionary study system.

## Availability of Supporting Data and Materials

The trio-binned assemblies, annotations, and all raw sequencing data for *Arctia plantaginis* reported in this article are available under ENA study accession No. PRJEB36595. All supporting data and materials are available in the *GigaScience* GigaDB database [80].

## Additional Files

**Supplementary Figure S1:** PacBio read length distribution for the *Arctia plantaginis* F1 offspring genome.

**Supplementary Figure S2:** *k*-mer blob plot visualizing haplotype-specific *k*-mers for *Arctia plantaginis*.

**Supplementary Figure S3**: GenomeScope profile of the *Arctia plantaginis* F1 offspring genome.

**Supplementary Figure S4:** Comparison of structural variant sizes between the *Arctia plantaginis* trio-binned haplotype assemblies.

**Supplementary Figure S5:** Cytogenetic analysis of *Arctia plantaginis* sex chromosomes.

**Supplementary Text S1:** Results for cytogenetic analysis of *Arctia plantaginis* sex chromosomes.

**Supplementary Text S2:** Method for estimating wild *Arctia plantaginis* genome heterozygosity.

**Supplementary Table S1:** Exact sampling localities of wild *Arctia plantaginis* males used in population genomic analysis.

**Supplementary Table S2:** Resequenced genome statistics for wild *Arctia plantaginis* males used in population genomic analysis.

**Supplementary Table S3:** Full BUSCO summary for *Arctia plantaginis* and 7 publicly available lepidopteran genome assemblies.

**Supplementary Table S4:** Heterozygosity per male in the wild Finnish *Arctia plantaginis* population.

**Supplementary Table S5:** Structural variant sizes present between the *Arctia plantaginis* trio-binned haplotype assemblies.

## Abbreviations

bp: base pairs; BUSCO: Benchmarking Universal Single-Copy Orthologs; BWA: Burrows-Wheeler Aligner; CLR: continuous long reads; CTAB: hexadecyltrimethylammonium bromide; Cy3-dUTP: cyanine 3-deoxyuridine triphosphate; DABCO: 1,4-diazabicyclo[2.2.2]octane; DAPI: 1,4-diazabicyclo[2.2.2]octane; ENA: European Nucleotide Archive; FS: Fisher strand bias; GATK: Genome Analysis Tool Kit; GISH: genomic *in situ* hybridization; GTRGAMMA: generalized time-reversible substitution model and gamma model of rate heterogeneity; KAT: Kmer Analysis Toolkit; kb: kilobase pairs; LD: linkage disequilibrium, Mb: megabase pairs, ML: maximum likelihood; MQ: root mean square mapping quality; MQRankSum: mapping quality rank sum test; PacBio: Pacific Biosciences; PC: principal component; PCA: principal component analysis; QD: quality by depth; QV: quality value; RAxML: Random Axelerated Maximum Likelihood; ReadPosRankSum: read position rank sum test; SMRT: Single Molecule, Real-Time; SNP: single-nucleotide polymorphism; SOR: strand odds ratio; STAR: Spliced Transcripts Alignment to a Reference; SV: structural variant.

## Competing Interests

The authors declare that they have no competing interests.

## Funding

C.D.J., E.C.Y., T.N.G., J.I.M., and I.A.W. were supported by the European Research Council Speciation Genetics advanced grant (No. 339873) and the Biotechnology and Biological Sciences Research Council (No. BB/R007500/1) to perform DNA extraction, sequencing, and genome annotation and population genomic analysis. S.A.M. and R.D. were supported by the Wellcome Trust (No. WT207492) to perform genome assembly. S.P. was supported by the Wellcome Trust (No. WT206194) to perform genome curation. T.N.G. was supported by the Biotechnology and Biological Sciences Research Council (No. BB/M011194/1) to perform genome annotation. J.A.G. and J.M. were supported by the Academy of Finland (project No. 320438 and 328474) and Jyväskylän Yliopisto to perform family rearing and fieldwork. P.N. was supported by the Grantová Agentura České Republiky (Reg. No. 20-20650Y) to perform cytogenetic analysis.

## Authors' Contributions

C.D.J. conceived and provided funding for the study. J.A.G. and J.M. performed rearing and fieldwork, for which J.M. provided funding. E.C.Y. and I.A.W. performed genomic extractions. S.A.M. performed genome assembly and R.D. provided funding. S.P. performed genome curation. T.N.G. performed genome annotation. P.N. performed cytogenetic analysis. E.C.Y. performed comparative quality assessment. E.C.Y. performed population genomic analysis, with contributions from J.I.M. E.C.Y., S.A.M., and P.N. produced figures. E.C.Y. wrote the manuscript with contributions from J.A.G., S.A.M., T.N.G., and P.N. and input from all authors.

## Supplementary Material

giaa088_GIGA-D-20-00060_Original_Submission

giaa088_GIGA-D-20-00060_Revision_1

giaa088_Response_to_Reviewer_Comments_Original_Submission

giaa088_Reviewer_1_Report_Original_Submission Annabel Charlotte Whibley -- 3/31/2020 Reviewed

giaa088_Reviewer_2_Report_Original_Submission Shanlin Liu -- 4/12/2020 Reviewed

giaa088_Supplemental_File
