# Correction to: A haplotype-resolved, de novo genome assembly for the wood tiger moth (*Arctia plantaginis*) through trio binning

**DOI:** 10.1093/gigascience/giab073

**Published:** 2021-10-23

**Authors:** Eugenie C Yen, Shane A McCarthy, Juan A Galarza, Tomas N Generalovic, Sarah Pelan, Petr Nguyen, Joana I Meier, Ian A Warren, Johanna Mappes, Richard Durbin, Chris D Jiggins

**Affiliations:** Department of Zoology, University of Cambridge, Downing Street, Cambridge CB2 3EJ, UK; Department of Genetics, University of Cambridge, Downing Street, Cambridge CB2 3EH, UK; Wellcome Sanger Institute, Wellcome Trust Genome Campus, Hinxton, Saffron Walden CB10 1SA, UK; Department of Biological and Environmental Science, University of Jyväskylä, FI-40014 Jyväskylä, Finland; Department of Zoology, University of Cambridge, Downing Street, Cambridge CB2 3EJ, UK; Wellcome Sanger Institute, Wellcome Trust Genome Campus, Hinxton, Saffron Walden CB10 1SA, UK; Biology Centre of the Czech Academy of Sciences, Institute of Entomology, Branišovská 1160/31, 370 05 České Budějovice, Czech Republic; University of South Bohemia, Faculty of Science, Branišovská 1645/31A, 370 05 České Budějovice, Czech Republic; Department of Zoology, University of Cambridge, Downing Street, Cambridge CB2 3EJ, UK; St John's College, University of Cambridge, St John's Street, Cambridge CB2 1TP, UK; Department of Zoology, University of Cambridge, Downing Street, Cambridge CB2 3EJ, UK; Department of Biological and Environmental Science, University of Jyväskylä, FI-40014 Jyväskylä, Finland; Department of Genetics, University of Cambridge, Downing Street, Cambridge CB2 3EH, UK; Wellcome Sanger Institute, Wellcome Trust Genome Campus, Hinxton, Saffron Walden CB10 1SA, UK; Department of Zoology, University of Cambridge, Downing Street, Cambridge CB2 3EJ, UK; St John's College, University of Cambridge, St John's Street, Cambridge CB2 1TP, UK

This is a correction to:

GigaScience, Volume 9, Issue 8, August 2020, giaa088, https://doi.org/10.1093/gigascience/giaa088

In the original version of the article “A haplotype-resolved, de novo genome assembly for the wood tiger moth (*Arctia plantaginis*) through trio binning” by Eugenie C. Yen et al. [1] there were a number of mis-reported statistics in Table 1.

**Table:** Table 1.

Genome annotation statistics		iArcPla.TrioW (paternal)	iArcPla.TrioY (maternal)
Total Genome size (bp)		584 621 344	577 993 050
Repetitive sequences (bp)		239 949 688	247 356 128
Masked repeats (%)		41.04	42.8
Mapped RNA-seq reads (n)		599 065 138	590 780 528
Mapped RNA-seq reads (%)		95.45	94.13
Protein-coding genes (n)		19 899	18 894
Mean gene length (bp)		5 966	5 951
BUSCO Completeness (%; n:1658)		98	95.9
Repeat Elements (n)	Total	1 220 592	1 232 654
	DNA Transposons	366 732	372 834
	LTR	127 169	139 770
	LINES	425 833	433 388
	SINES	43 022	71 790
	Unclassified	257 836	214 872

In the bottom section, the result is incorrect as this is the number of element types identified rather than the number of elements identified in the genome as these types may occur multiple times. Therefore, the correct number of elements that should be reported are as below:

The numbers have been corrected in the published manuscript, and the authors apologize for the error.
