# Supplementary material for: A haplotype-resolved, de novo genome assembly for the wood tiger moth (Arctia plantaginis) through trio binning
Source: Gigascience. 2020 Aug 18;9(8):giaa088. doi: 10.1093/gigascience/giaa088 (PMC7433188; doi:10.1093/gigascience/giaa088)
Supplement: giaa088_GIGA-D-20-00060_Revision_1 [file giaa088_giga-d-20-00060_revision_1.pdf]

GigaScience

A haplotype-resolved, de novo genome assembly for the wood tiger moth (Arctia plantaginis) through trio binning

--Manuscript Draft--

**Manuscript Number:**	GIGA-D-20-00060R1
**Full Title:**	A haplotype-resolved, de novo genome assembly for the wood tiger moth (Arctia plantaginis) through trio binning
**Article Type:**	Data Note
**Funding Information:**	European Research Council (339873) — Prof Chris D. Jiggins<br>Wellcome Trust (WT207492) — Prof Richard Durbin<br>Wellcome Trust (WT206194) — Ms Sarah Pelan<br>Academy of Finland (320438) — Prof Johanna Mappes<br>Academy of Finland (328474) — Prof Johanna Mappes<br>Czech Science Foundation (20-20650Y) — Dr Petr Nguyen

Abstract:

Background

Diploid genome assembly is typically impeded by heterozygosity, as it introduces errors when haplotypes are collapsed into a consensus sequence. Trio binning offers an innovative solution which exploits heterozygosity for assembly. Short, parental reads are used to assign parental origin to long reads from their F1 offspring before assembly, enabling complete haplotype resolution. Trio binning could therefore provide an effective strategy for assembling highly heterozygous genomes which are traditionally problematic, such as insect genomes. This includes the wood tiger moth (Arctia plantaginis), which is an evolutionary study system for warning colour polymorphism.

Findings

We produced a high-quality, haplotype-resolved assembly for Arctia plantaginis through trio binning. We sequenced a same-species family (F1 heterozygosity ~1.9%) and used parental Illumina reads to bin 99.98% of offspring Pacific Biosciences reads by parental origin, before assembling each haplotype separately and scaffolding with 10X linked-reads. Both assemblies are highly contiguous (mean scaffold N50: 8.2Mb) and complete (mean BUSCO completeness: 97.3%), with complete annotations and 31 chromosomes identified through karyotyping. We employed the assembly to analyse genome-wide population structure and relationships between 40 wild resequenced individuals from five populations across Europe, revealing the Georgian population as the most genetically differentiated with the lowest genetic diversity.

Conclusions

We present the first invertebrate genome to be assembled via trio binning. This assembly is one of the highest quality genomes available for Lepidoptera, supporting trio binning as a potent strategy for assembling highly heterozygous genomes. Using this assembly, we provide genomic insights into geographic population structure of Arctia plantaginis.

Corresponding Author: Eugenie Yen, BA (Hons), MPhil
University of Cambridge
Cambridge, Cambridgeshire UNITED KINGDOM

Corresponding Author Secondary Information:

Powered by Editorial Manager® and ProduXion Manager® from Aries Systems Corporation

**Corresponding Author's Institution:**	University of Cambridge
**Corresponding Author's Secondary Institution:**	
**First Author:**	Eugenie C Yen, BA (Hons), MPhil
**First Author Secondary Information:**	
**Order of Authors:**	Eugenie C Yen, BA (Hons), MPhil
	Shane A. McCarthy
	Juan A. Galarza
	Tomas N. Generalovic
	Sarah Pelan
	Petr Nguyen
	Joana I. Meier
	Ian A. Warren
	Johanna Mappes
	Richard Durbin
	Chris D. Jiggins
**Order of Authors Secondary Information:**	
**Response to Reviewers:**	Please see below for our point by point response to specific reviewer comments, and please see our attached Word document "Yen Response to Reviewers.docx" for a formatted version of the below text.<br><br>Reviewer 1 Specific Comments<br><br>"General<br>The resolution of the figures in the main submission, but not the supplement, is a little poor in the review copy."<br><br>We checked the resolution of our submitted figures, and we determine this issue should be specific to the reviewer copy only.<br><br>"Background<br>While I agree that full diploid reconstruction is/should be a eukaryotic genome assembly target and that there are few published examples, it might be worth also noting that the Vertebrate Genome Project contains, I believe, 12 trio-based assemblies that are publicly accessible."<br><br>We thank the reviewer for pointing this out, and we agree that the mentioned assemblies should be included. We have counted the number of trio-based assemblies currently available on the VGP GenomeArk data and changed the sentence on page 4 accordingly:<br><br>This represents the first trio binned assembly available for Insecta and indeed any invertebrate animal species, diversifying the organisms for which trio binning has been applied outside of bovids [6, 7], zebra finches [9], humans [6, 9, 10] and Arabidopsis thaliana [6].<br>to:<br>At the time of writing, this represents the first trio binned assembly available for an invertebrate animal species, diversifying the organisms for which published trio binned assemblies exist beyond bovids [6, 7], zebra finches [9], humans [6, 9, 10], Arabidopsis thaliana [6] and additional trio binned assemblies available for eight other vertebrate species on the Vertebrate Genomes Project GenomeArk database [11].<br><br>with added reference:

11. Vertebrate Genomes Project GenomeArk. https://vgp.github.io/genomeark. Accessed May 2020.

"Methods
Please confirm that you have not done any of the following (and if you have, please incorporate details in the methods)
Any additional quality trimming of RNAseq reads beyond adaptor removal with cutadapt?"

We have added the suggested details on page 9 by changing:

RNA-seq reads were trimmed for adapter contamination using cutadapt version 1.8.1 [48] and quality controlled pre and post trimming with fastqc version 0.11.8 [49].
to:
Using cutadapt version 1.8.1 [56], RNA-seq reads were trimmed for adapter contamination and quality trimmed at both ends of each read using a quality value of 3 (-q 3,3). Quality control was performed pre and post trimming with fastqc version 0.11.8 [57].

"Any pre-processing of PacBio reads to remove adaptor contamination etc?"

We confirm that there was no pre-processing of PacBio reads. We performed adapter contamination removal during the assembly curation stage, for which details have been added in our response to the reviewer comment "Can you elaborate further on the types of artefact/contamination/manual curation that was flagged by gEval?"

"Please consider also calculating and reporting QV to provide an estimate of assembly accuracy (presenting figures before and after polishing with the 10x reads would be of interest)."

We thank the reviewer for this suggestion and agree that reporting QV is useful. We have included a QV analysis in our revised manuscript. We have added a sentence describing the method on page 8:

To provide an estimate of assembly consensus accuracy, a quality value (QV) was computed for each assembly using Merqury version 1.0 [34].

and added a sentence describing the results on page 12-13:

Using Merqury [34], we estimated QV scores of Q34.7 for the paternal (iArcPla.TrioW) assembly and Q34.2 for the maternal (iArcPla.TrioY) assembly, indicating high (>99.9%) assembly accuracy.

with added reference:

34. Rhie A, Walenz BP, Koren S et al. Merqury: reference-free quality and phasing assessment for genome assemblies, BioRxiv. 2020; doi: https://doi.org/10.1101/2020.03.15.992941.

For the interest of the reviewer, QV prior to Illumina polishing was Q33.2 for the paternal assembly and Q32.7 for the maternal assembly. In VGP and other places, we are aiming for Q40, but in this case, we are lower than this likely due to the lower coverage we had per-haplotype (~25x per-haplotype).

"Can you elaborate further on the types of artefact/contamination/manual curation that was flagged by gEval?"

We have added the suggested details on page 7 by changing:

The assemblies were checked for contamination and further manually assessed and corrected using gEVAL [25].
to:
Assembly contaminants were identified and removed by checking the assemblies

against vector/adapter sequences in the UniVec database [26], common contaminants in eukaryotes [27] and organelle sequences [28, 29]. The assemblies were also checked against other organism sequences from the RefSeq database version 94 [30]. This identified mouse contamination in two scaffolds which were subsequently removed. The assemblies were further manually assessed and corrected using gEVAL [31] with the available PacBio and 10X data. This process involved locating regions of zero or extreme PacBio read coverage and missed or mis-joins indicated by the 10X data, then evaluating the flagged discordances and correcting them where possible, which were typically missed joins, mis-joins and false duplications.

with added references:

26. UniVec Database. NCBI. https://www.ncbi.nlm.nih.gov/tools/vecscreen/univec. Accessed March 2019.

27. Contam_in_euks.fa.gz. ftp://ftp.ncbi.nlm.nih.gov/pub/kitts/contam_in_euks.fa.gz. Accessed March 2019.

28. Mito.nt.gz. ftp://ftp.ncbi.nlm.nih.gov/blast/db/ FASTA/mito.nt.gz. Accessed March 2019.

29. RefSeq Plastid Database. NCBI. ftp://ftp.ncbi.nlm.nih.gov/refseq/release/plastid. Accessed March 2019.

30. RefSeq: NCBI Reference Sequence Database. www.ncbi.nlm.nih.gov/refseq. Accessed March 2019.

"I am interested to know more about how you constructed the plots in supplementary figure 1 (e.g. is this from custom parsing of reads lengths/counts in R or a direct visualisation of output from the assembler?). I ask with the vague hope that such qc descriptions might eventually become standardised so that direct comparisons of such metrics between assemblies might become straightforward."

For the interest of the reviewer, the plots in Supplementary Figure 1 are based on a Dazzler database (https://github.com/thegenemyers/DAZZ_DB) of the raw data. The command DBstats provided by DAZZ_DB outputs the histogram data used for these plots. We have a simple R script which parses this histogram data to make these plots from the database. This usually needs to be tweaked for individual datasets and for making a plot appropriate for a paper. We have added a sentence to the legend of Supplementary Figure 1 to briefly explain how the plot was constructed on page 2 of the Supplementary Material:

Plots were constructed from a Dazzler database [Supplementary Reference 1] of the raw data, using histogram data outputted by the ‘DBstats’ command.

with added supplementary reference:

1. The Dazzler Database Library. https://github.com/thegenemyers/DAZZ_DB. Accessed March 2019.

"The treatment of the population samples (extraction and sequencing) is the same as for the parental short read sequencing. You could refer back to the earlier description here to avoid repetition."

We have implemented this suggestion on page 11 by replacing the repeated description with:

Whole genomic DNA extraction and short read sequencing was performed following the same method as described for short read sequencing of parental genomes during trio binning assembly.

For clarity, perhaps elaborate briefly on the samples/tissue types within the published RNAseq dataset you use for annotation

Powered by Editorial Manager® and ProduXion Manager® from Aries Systems Corporation

	We have added this content on page 9 by changing:<br><br>Raw RNA-seq reads were obtained from Galarza et al. 2017 [47] under study accession number PRJEB14172<br>to:<br>Raw RNA-seq reads were obtained from Galarza et al. 2017 [55] under study accession number PRJEB14172, which came from whole body tissue of A. plantaginis larvae from two families reared under two heat treatments.<br><br>"Discussion<br>Prompted by your statement "Successful haplotype separation was possible due to the high estimated heterozygosity…", it might be interesting to explore further how relevant the degree of heterozygosity really is to the success of this approach. Your statement is certainly right for highly fragmented assemblies but with long contigs, it is my sense that even a substantially lower degree of heterozygosity can still give strong support to contig origin and thus fully resolve the haplotypes."<br><br>We thank the reviewer for drawing attention to this statement. We have changed the statement on page 13:<br><br>Successful haplotype separation was possible due to the high estimated heterozygosity…<br>to:<br>Successful haplotype separation was facilitated by the high estimated heterozygosity…<br><br>with a corresponding change to a similar statement on page 4:<br><br>This was possible due to the high heterozygosity of the A. plantaginis genome…<br>to:<br>This was facilitated by the high heterozygosity of the A. plantaginis genome…<br><br>We recognise that trio binning can be successfully applied to organisms with lower heterozygosity. Indeed, the other species with published trio binned assemblies that we reference in our manuscript all have lower heterozygosities, ranging down to 0.1% (humans) in the original trio binning method paper Koren et al. 2018 (our reference [6]). We do not believe it is appropriate to our manuscript to further investigate how changing heterozygosity affects the success of the trio binning method, since our manuscript is about the application of trio binning for the assembly of a single species, and not about the method itself. Furthermore, this has already been addressed in the original Koren et al. 2018 paper (our reference [6]), which considers crosses with a range of heterozygosities, with an Arabidopsis thaliana cross (1.4%), Homo sapiens cross (0.1%) and Bos taurus x Bos indicus cross (0.9%), and discusses how higher heterozygosity enables the trio binning method work better.<br><br>We have included a sentence referring to this discussion about heterozygosity in Koren et al. 2018 (our reference [6]) in the revised manuscript. We also note that we only discuss the yak-cow hybrid heterozygosity value of 1.2% as a comparison, when in fact within species heterozygosity for previously published trio binned assemblies for zebra finch (1.6%) and Arabidopsis (1.4%) are both higher. We have therefore included a comparison to species heterozygosity from all previously published trio binned assemblies to improve our discussion breadth. These changes are located on page 13:<br><br>Successful haplotype separation was possible due to the high estimated heterozygosity (~1.9%) of the F1 offspring genome (Supplementary Figure 3), with greater levels of heterozygosity achieved through our same-species A. plantaginis cross than previously achieved through an inter-species cross between yak (Bos grunniens) and cattle (Bos taurus), which gave an F1 heterozygosity of ~1.2% [7].<br>to:<br>Successful haplotype separation was facilitated by the high estimated heterozygosity (~1.9%) of the F1 offspring genome (Supplementary Figure 3), as it has previously been discussed that higher heterozygosity makes trio binning easier [6]. Indeed, greater heterozygosity levels were obtained through our same-species A. plantaginis cross than obtained previously through same-species crosses for zebra finch (~1.6%) [9], Arabidopsis (~1.4%) [6], bovid (~0.9%) [6] and human (~0.1%) [6] trio binned

assemblies, as well as an inter-species yak (Bos grunniens) x cattle (Bos taurus) cross (~1.2%) [7].

with a corresponding change on page 4:

… heterozygosity of the F1 offspring was estimated to be ~1.9%, exceeding levels (~1.2%) obtained when crossing different bovid species [7].
to:
… heterozygosity of the F1 offspring was estimated to be ~1.9%, exceeding levels obtained in all other published trio binned assemblies through same-species crosses [6, 9, 10] and a yak-cow hybrid cross [7].

"Please consider including some mention of how obtaining appropriate trio samples may be a challenge in non-traditional model systems."

We thank the reviewer for this suggestion, and we have included this content to page 19 by adding the sentence:

Our assembly further highlights that trio binning can work well for a non-model system, provided a family trio can be obtained, which remains challenging for many non-model systems where it is difficult to obtain both parents and rear their offspring.

"It is probably beyond the scope of this manuscript to touch on possible extensions of this approach to polyploid situations, but potentially this could be raised in the discussion."

We agree with the reviewer that this is beyond the scope of our manuscript. This is because we are not presenting our work as a novel method, but as an application of a previously published method to a new species, and note that reference [6] already briefly discusses the potential for applying similar ideas to polyploids.

"Rather than "top tier" perhaps consider using "platinum quality", which seems to be gaining increasing use as a descriptor for assemblies with full chromosome scaffolds and haplotypes resolved across the entire genome."

We thank the reviewer for drawing attention to this statement. We have altered our statement on page 15:

Future chromosomal-level scaffolding work through Hi-C scaffolding technology [67] will elevate the A. plantaginis assembly quality to the top tier.
to:
Future scaffolding work has the potential to lead to a chromosomal-scale A. plantaginis assembly.

We believe the revised statement is more informative because it is often unclear what descriptors like “top tier” and “platinum quality” mean, as they are continually being redefined and debated. We have also removed the statement and reference about Hi-C scaffolding technology, since Hi-C is not the only way to achieve chromosomal-scale assemblies, so our original discussion statement is too narrow and potentially confusing.

Reviewer 2 Specific Comments

"1. the authors may want to explain more on the results from the KAT (Kmer based) analysis. For example, how did you obtain the initial Kmer set, from your assemblies or the shotgun reads? If you distinguished single-copy and multiple copy Kmers by tallying their occurrence number in the parental and maternal genomes, how did you define those 0-copy Kmer?
In addition, what is the proportion of your Kmer set that was utilized in the KAT analysis comparing to the entire Kmer set which can be obtained from the genome assembly or the shotgun reads. Will enlarge the K value help to increase the proportion and in turn, increase the power of the analysis?"

Powered by Editorial Manager® and ProduXion Manager® from Aries Systems Corporation

	We thank the reviewer for this feedback. To answer the reviewer's questions, KAT plots a histogram of the frequencies of all of the Kmers in the raw read data set, coloured by the number of times that the Kmer appears in the assembly. 0-copy Kmers (shown in black in Figure 2) are those found in the raw reads but not in the assembly. Changing the value of K does not change the proportion of Kmers used because we are using all Kmers for any value of K. Enlarging the value of K will increase the fraction of Kmers in the error (0-copy) and haploid (1-copy) peaks at the expense of the diploid (2-copy) peak, since a single discrepancy in a run of diploid sequence will affect K Kmers. We used a standard value of K=21 which clearly identifies error, haploid and diploid peaks for this species and data set. We have clarified these points in our manuscript further by changing the sentence in the legend of Figure 2 on page 28:<br><br>The first peak corresponds to k-mers missing from the assembly due to sequencing errors…<br>to:<br>The first peak corresponds to k-mers present in the raw reads but missing from the assembly due to sequencing errors…<br><br>and added a sentence describing the chosen cut-off K value on page 8:<br><br>For this analysis we used parameter K=21, which clearly identified error, haploid and diploid peaks for our dataset.<br><br>"2. the authors claim a whole genome heterozygosity level of 1.9% for the wood tiger moth, which, however, is estimated using a Kmer based method before obtaining the genome assembly. As you have already obtained the high-quality genome assembly, you may want to re-calculate it, and also it will be great to show readers that how the heterozygous sites distribute on the genome and briefly categorize them according to their types, e.g. SNPs, small InDels and large structure variances(SVs). Validating and visualizing those heterozygous sites makes the quality assessment part more complete."<br><br>We thank the reviewer for this suggestion, and we have included a heterozygosity analysis using the genome assembly in our revised manuscript. We estimated heterozygosity for a wild Finnish population (n=20), using resequenced genomes available from our population genomics analysis. We chose to estimate heterozygosity for this population as the parents used for trio binning assembly were from selection lines derived from a natural Finnish population, making this comparison highly relevant. This comparison is further useful to show that our reference genome is still representative of natural variation in the wild, which is important for population genomic studies.<br><br>To perform this analysis, we selected BAM files for the 20 Finnish individuals and called variants with monomorphic sites for the 5 largest scaffolds in the iArcPla.TrioW reference assembly. This subsample is representative of the whole genome as it covers 96.5 Mbp (15%) of the total assembly. The raw callset was filtered in the same way as performed in our population genomics analysis, then the number of SNPs and indels was calculated for each individual using VCFtools with a minor allele count filter of 1, to filter out sites which were different to the reference assembly in all individuals. We then computed individual heterozygosity by dividing the total number of SNPs and indels by the total number of sites (minus the number of missing sites) per individual. This gave a mean heterozygosity value of ~1.8% across all individuals. This value is highly similar to our estimated heterozygosity for the F1 offspring genome (~1.9%), strengthening our result from kmer analysis. The slightly lower value in the wild might be explained by the parents used in our family trio being derived from different selection lines (3 generations), leading to greater heterozygosity between the trio binned parental haplotypes.<br><br>We have added Supplementary Text 2 (page 7 of Supplement) to describe the method for our heterozygosity analysis, and we have added Supplementary Table 4 (page 11 of Supplement), to report the number of SNPs, indels, total sites, and heterozygosity estimate per individual. On page 13 of our revised manuscript, we have changed:<br><br>Using GenomeScope, we estimated the F1 offspring haploid genome size to be 590Mb

Powered by Editorial Manager® and ProduXion Manager® from Aries Systems Corporation

with a repeat fraction of 27% (Supplementary Figure 3).
to:
Using GenomeScope [35], we estimated the F1 offspring haploid genome size to be 590Mb with a repeat fraction of 27% and whole genome heterozygosity of ~1.9% (Supplementary Figure 3). This value was similar to our mean heterozygosity estimate of ~1.8% in a wild, Finnish population (Supplementary Table 4; method described in Supplementary Text 1), demonstrating our reference assembly is representative of natural variation in a wild population. The slight discrepancy may be explained by the parents used for trio binning assembly being derived from different selection lines, leading to greater heterozygosity between the trio binned parental haplotypes.

In response to the reviewer's suggestion, we have included an analysis of SVs present between the trio binned parental haplotypes. To do this, we performed a whole genome alignment between the parental haplotype assemblies and used Assemblytics to detect SVs, which is the same method used in the original trio binning paper Koren et al. 2016 (our reference [6]). Assemblytics reports the number and total bp affected by insertions, deletions, tandem expansions, tandem contractions, repeat expansions and repeat contractions, for size ranges of 50-500 bp and 500-10000 bp.

We have added a sentence describing our method on page 8:

Assemblytics [36] was used to detect structural variants (SVs) between the parental haplotypes. For this, a whole-genome alignment was performed between the haplotype assemblies using the Nucmer module of MUMmer version 3.23 [37] with Assemblytics recommended options.

with a corresponding description of our results to page 13:

Assemblytics [36] detected 32203 SVs between the haplotype assemblies, affecting 51.6 Mbp of the genome (Supplementary Table 5; Supplementary Figure 4).

and added references:

36. Nattestad M, Schatz MC. Assemblytics: a web analytics tool for the detection of variants from an assembly. Bioinformatics. 2016; 32: 3021-3023.

37. Kurtz S, Phillippy A, Delcher AL et al. Versatile and open software for comparing large genomes. Genome Biology. 2004; 5: R12.

We have added Supplementary Figure 4 (page 5 of Supplement) and Supplementary Table 5 (page 11 of Supplement) to report and visualise the distribution of SV sizes present between the alignment of the parental haplotype assemblies.

Whilst we agree it would be interesting to characterise large SVs further, we believe that this type of extensive analysis is beyond the scope of our manuscript, which is a short Data Note to demonstrate the application of the trio binning method to another new species. We do not believe it is appropriate in our manuscript to visualise how heterozygous sites distribute across the genome, as we do not yet have an ordered, chromosomal-scale assembly, so this information would not be as useful at this moment in time. We further think that visualising heterozygosity along the genome would only be valuable if combined with a thorough investigation of the driving factors of the heterozygosity variation (such as selection, recombination, gene content etc.), which we also feel is beyond the scope of this Data Note paper. Without adding the suggested analysis, we maintain that we have provided a robust quality assessment of our trio binned reference assembly through KAT visualisation, the newly added QV analysis and the comparative assessment of contiguity metrics and BUSCO gene completeness against an unbinned assembly and 7 publicly available lepidopteran genomes, which place our assembly within the context of Lepidoptera genomics and clearly demonstrates it to be one of the best assemblies currently available for Lepidoptera.

"3. the authors may want to give the unbinned data based assembly a more integrity process, so that makes a fair comparison. For example, you did not apply the 10X data to further scaffold the assembly, or maybe you have but I missed it. You'd better clarify

it somewhere in your manuscript."

We thank the reviewer for this suggestion, and agree it would facilitate a fairer comparison than the one we report between scaffolded trio binned assemblies and an unscaffolded unbinned assembly. We have implemented the suggestion whilst avoiding the intensive process of producing a new assembly, by comparing unscaffolded versions of the trio binned assemblies against the unbinned assembly, which were all assembled using wtdbg2 followed by one round of Arrow polishing. We therefore compare binned and unbinned assemblies which are both unscaffolded, achieving a fair comparison in an equivalent manner to if we compare binned and unbinned assemblies which are both scaffolded, as suggested by the reviewer. Furthermore, the newly included summary statistics for the unscaffolded trio binned assemblies can also be compared against the scaffolded trio binned assemblies, adding information on the quality improvement after scaffolding with 10X data.

In our revised manuscript, we have altered the methods on page 8:

Quality comparisons were conducted against an assembly of unbinned data from the same F1 offspring (iArcPla.wtdbg2), and against a representative selection of published lepidopteran reference genomes. For this, the latest versions of seven Lepidoptera species were downloaded…
to:
A quality comparison was conducted by comparing unscaffolded, Arrow polished versions of the trio binned assemblies against an unscaffolded, Arrow polished assembly of unbinned data from the same F1 offspring (iArcPla.wtdbg2). Quality comparisons were also performed for the final, scaffolded trio binned assemblies against a representative selection of published lepidopteran reference genomes, for which the latest versions of seven Lepidoptera species were downloaded…

and changed the results on page 14-15:

The paternal (iArcPla.TrioW) assembly contains 1069 scaffolds and N50=6.73 Mb, and the maternal (iArcPla.TrioY) assembly contains 1050 scaffolds and N50=9.77 Mb (Table 2). Both trio binned assemblies are more contiguous than the composite haploid iArcPla.wtdbg2 assembly produced using unbinned data from the same individual, which contains 2948 scaffolds and N50=1.84 Mb (Table 2; Figure 3A), illustrating the contiguity improvement we achieved by separating haplotypes before assembly. The trio binned assemblies are more complete than the unbinned assembly (complete BUSCOs: iArcPla.TrioW=98.1%; iArcPla.TrioY=96.4%; iArcPla.wtdbg2=95.4%). The trio binned assemblies are also less inflated than the unbinned assembly (assembly size: iArcPla.TrioW=585 Mb; iArcPla.TrioY=578 Mb; iArcPla.wtdbg2=615 Mb) and duplicated BUSCOs halved (duplicated BUSCOs: iArcPla.TrioW=1.2%; iArcPla.TrioY=1.1%; iArcPla.wtdbg2=2.1%), suggesting a reduction in artefactual assembly duplication at heterozygous sites through read binning (Table 2; Figure 3A).
to:
The paternal (iArcPla.TrioW) assembly contains 1069 scaffolds with N50=6.73 Mb and 98.1% complete BUSCOs, and the maternal (iArcPla.TrioY) assembly contains 1050 scaffolds with N50=9.77 Mb and 96.4% complete BUSCOs (Table 3). Prior to scaffolding work with 10X data, both unscaffolded trio binned assemblies are already more contiguous and complete than a composite, haploid iArcPla.wtdbg2 assembly produced using unbinned data from the same individual (Table 2; Figure 3A). This illustrates the quality improvement achieved by separating haplotypes before assembly, and further improvement of the trio binned assemblies after scaffolding with 10X linked-reads (Table 2). The trio binned assemblies are also less inflated than the unbinned assembly with halved duplicated BUSCOs (Table 2; Figure 3A), suggesting a reduction in artefactual assembly duplication at heterozygous sites through read binning.

We have added quality statistics for the unscaffolded trio binned assemblies to Table 2 (page 16) and Supplementary Table 3 (page 10 of Supplement). We have also revised Figure 3A to show the revised cumulative contig length plot, and altered its legend on page 29:

Comparison of the A. plantaginis trio binned assemblies iArcPla.TrioW (paternal

Powered by Editorial Manager® and ProduXion Manager® from Aries Systems Corporation

	haplotype) and iArcPla.TrioY (maternal haplotype) against the composite assembly using unbinned data from the same individual (iArcPla.wtdbg2).<br>to:<br>Comparison of the unscaffolded A. plantaginis trio binned assemblies iArcPla.TrioW (paternal haplotype) and iArcPla.TrioY (maternal haplotype) against the unscaffolded composite assembly using unbinned data from the same individual (iArcPla.wtdbg2).
Additional Information:	
Question	Response
Are you submitting this manuscript to a special series or article collection?	No
**Experimental design and statistics**<br><br>Full details of the experimental design and statistical methods used should be given in the Methods section, as detailed in our Minimum Standards Reporting Checklist. Information essential to interpreting the data presented should be made available in the figure legends.<br><br>Have you included all the information requested in your manuscript?	Yes
**Resources**<br><br>A description of all resources used, including antibodies, cell lines, animals and software tools, with enough information to allow them to be uniquely identified, should be included in the Methods section. Authors are strongly encouraged to cite Research Resource Identifiers (RRIDs) for antibodies, model organisms and tools, where possible.<br><br>Have you included the information requested as detailed in our Minimum Standards Reporting Checklist?	Yes
**Availability of data and materials**<br><br>All datasets and code on which the conclusions of the paper rely must be either included in your submission or deposited in publicly available repositories (where available and ethically	Yes

appropriate), referencing such data using a unique identifier in the references and in the “Availability of Data and Materials” section of your manuscript.<br><br>Have you have met the above requirement as detailed in our Minimum Standards Reporting Checklist?	

Powered by Editorial Manager® and ProduXion Manager® from Aries Systems Corporation

Manuscript Click here to access/download;Manuscript;060-final-HL.docx

A haplotype-resolved, *de novo* genome assembly for the wood tiger moth (*Arctia plantaginis*) through trio binning

Eugenie C. Yen[1*], Shane A. McCarthy[2,3], Juan A. Galarza[4], Tomas N. Generalovic[1], Sarah Pelan[3], Petr Nguyen[5,6], Joana I. Meier[1,7], Ian A. Warren[1], Johanna Mappes[4], Richard Durbin[2,3] and Chris D. Jiggins[1,7]

[1] Department of Zoology, University of Cambridge, Cambridge, CB2 3EJ, United Kingdom

[2] Department of Genetics, University of Cambridge, Cambridge, CB2 3EH, United Kingdom

[3] Wellcome Sanger Institute, Wellcome Trust Genome Campus, Hinxton, Cambridge, CB10 1SA, United Kingdom

[4] Department of Biological and Environmental Science, University of Jyväskylä FI-40014, Jyväskylä, Finland

[5] Biology Centre of the Czech Academy of Sciences, Institute of Entomology, 370 05 České Budějovice, Czech Republic

[6] University of South Bohemia, Faculty of Science, 370 05 České Budějovice, Czech Republic

[7] St John's College, CB2 1TP, Cambridge, United Kingdom

[*]Corresponding Author: Eugenie C. Yen. Department of Zoology, Downing Street, University of Cambridge, Cambridge, CB2 3EJ, UK. Email: eugeniecyen@gmail.com. Phone: +447402737277.

ORCIDs:
Eugenie C. Yen, 0000-0003-4992-782X;
Shane A. McCarthy, 0000-0002-2715-4187;

Juan A. Galarza, 0000-0003-3938-1798;
Petr Nguyen, 0000-0003-1395-4287;
Joana I. Meier, 0000-0001-7726-2875;
Johanna Mappes, 0000-0002-5936-7355;
Richard Durbin, 0000-0002-9130-1006;
Chris D. Jiggins, 0000-0002-7809-062X.

ABSTRACT

Background: Diploid genome assembly is typically impeded by heterozygosity, as it introduces errors when haplotypes are collapsed into a consensus sequence. Trio binning offers an innovative solution which exploits heterozygosity for assembly. Short, parental reads are used to assign parental origin to long reads from their F1 offspring before assembly, enabling complete haplotype resolution. Trio binning could therefore provide an effective strategy for assembling highly heterozygous genomes which are traditionally problematic, such as insect genomes. This includes the wood tiger moth (*Arctia plantaginis*), which is an evolutionary study system for warning colour polymorphism. **Findings:** We produced a high-quality, haplotype-resolved assembly for *Arctia plantaginis* through trio binning. We sequenced a same-species family (F1 heterozygosity ~1.9%) and used parental Illumina reads to bin 99.98% of offspring Pacific Biosciences reads by parental origin, before assembling each haplotype separately and scaffolding with 10X linked-reads. Both assemblies are highly contiguous (mean scaffold N50: 8.2Mb) and complete (mean BUSCO completeness: 97.3%), with complete annotations and 31 chromosomes identified through karyotyping. We employed the assembly to analyse genome-wide population structure and relationships between 40 wild resequenced individuals from five populations across Europe, revealing the Georgian population as the most genetically differentiated with the lowest genetic diversity. **Conclusions:** We present the first invertebrate genome to be assembled via trio binning. This assembly is one of the highest quality genomes available for Lepidoptera, supporting trio binning as a potent strategy for assembling highly heterozygous genomes. Using this assembly, we provide genomic insights into geographic population structure of *Arctia plantaginis*.

Keywords: wood tiger moth; *Arctia plantaginis*; Lepidoptera; genome assembly; trio binning; annotation; population genomics

DATA DESCRIPTION

Background

The ongoing explosion in *de novo* reference genome assembly for non-model organisms has been facilitated by the combination of advancing technologies and falling costs of next generation sequencing [1]. Long-read sequencing technologies further revolutionised the quality of assembly achievable, with incorporation of long reads that can span common repetitive regions leading to radical improvements in contiguity [2]. However, heterozygosity still presents a major challenge to *de novo* assembly of diploid genomes. Most current technologies attempt to collapse parental haplotypes into a composite, haploid sequence, introducing erroneous duplications through mis-assembly of heterozygous sites as separate genomic regions. This problem is exacerbated in highly heterozygous genomes, resulting in fragmented and inflated assemblies which impede downstream analyses [3, 4]. Furthermore, a consensus sequence does not represent either true, parental haplotype, leading to loss of haplotype-specific information such as allelic and structural variants [5]. Whilst reducing heterozygosity by inbreeding has been a frequent approach, rearing inbred lines is unfeasible and highly time consuming for many non-model systems, and resulting genomes may no longer be representative of wild populations.

Trio binning is an innovative, new approach which takes advantage of heterozygosity instead of trying to remove it [6]. In this method, a family trio is sequenced with short reads for both parents and long reads for an F1 offspring. Parent-specific k-mer markers are then identified from the parental reads and used to assign offspring reads into maternal and paternal bins, before assembling each parental haploid genome separately [6]. The ability of trio binning to accurately distinguish parental haplotypes increases at greater heterozygosity, with high-quality, *de novo* assemblies achieved for bovid genomes by crossing different breeds [6] and species [7] to maximise heterozygosity. Therefore, trio binning has the potential to overcome current difficulties faced by highly heterozygous genomes, which have typically evaded high-quality assembly through conventional methods.

We utilised trio binning to assemble a high-quality, haplotype-resolved reference genome for the wood tiger moth (*Arctia plantaginis*, NCBI:txid874455; formerly *Parasemia plantaginis* [8]). At the time of writing, this represents the first trio binned assembly available for an invertebrate animal species, diversifying the organisms for which published trio binned assemblies exist beyond bovids [6, 7], zebra finches [9], humans [6, 9, 10], *Arabidopsis thaliana* [6] and additional trio binned assemblies available for eight other vertebrate species on the Vertebrate Genomes Project GenomeArk database [11]. Using a family trio with same-species *A. plantaginis* parents, 99.98% of offspring reads were successfully binned into parental haplotypes. This was facilitated by the high heterozygosity of the *A. plantaginis* genome; heterozygosity of the F1 offspring was estimated to be ~1.9%, exceeding levels obtained in all other published trio-binned assemblies through same-species crosses [6, 9, 10] and a yak-cow hybrid cross [7]. Both resulting haploid assemblies are highly contiguous and complete, strongly supporting trio binning as an effective strategy for *de novo* assembly of heterozygous genomes.

The presented *A. plantaginis* assembly will also provide an important contribution to the growing collection of lepidopteran reference genomes [12]. Comparative phylogenomic studies will benefit from the addition of *A. plantaginis* to the phylogenomic dataset [13, 14], being the first species to be sequenced within the Erebidae family [8, 15], and the first fully haplotype-resolved genome available for Lepidoptera. *A. plantaginis* itself is an important evolutionary study system, being a moth species which uses aposematic hindwing colouration to warn avian predators of its unpalatability [16]. Whilst female hindwing colouration varies continuously from orange to red, male hindwings exhibit a discrete colour polymorphism maintained within populations (Figure 1), varying in frequency from yellow-white in Europe and Siberia, yellow-red in the Caucasus, and black-white in North America and Northern Asia [17, 18]. Hence, *A. plantaginis* provides a natural system to study the evolutionary forces that promote phenotypic diversification on local and global scales, for which availability of a high-quality, haplotype-resolved and annotated reference genome will now transform genetic research.

Materials and Methods

Cross preparation and sequencing

To obtain an *A. plantaginis* family trio, selection lines for yellow and white male morphs were created from Finnish populations at the University of Jyväskylä over three consecutive generations. Larvae were fed with wild dandelion (Taraxacum spp.) and reared under natural light conditions, with an average temperature of 25°C during the day and 15-20°C at night until pupations. A father from the white selection line and mother from the yellow selection line were crossed, then collected and dry-frozen along with their F1 pupae at -20°C in 1.5 ml (millilitre) sterile Eppendorf tubes.

For short-read sequencing of the father (sample ID: CAM015099; ENA accession number: ERS4285278) and mother (sample ID: CAM015100; ENA accession number: ERS4285279), DNA was extracted from adult thoraces using a QIAGEN DNeasy Blood & Tissue Kit (Qiagen, Germany) following the manufacturer's protocol, then library preparation and sequencing was performed by Novogene (China). Illumina NEBNext (New England Biolabs, United States) libraries were constructed with an insert size of 350 bp (base pair), following the manufacturer's protocol, and sequenced with 150 bp paired end reads on an Illumina NovaSeq 6000 platform (Illumina, United States; RRID:SCR_016387).

For long-read sequencing of a single F1 pupal offspring (Sample ID: CAM015101; ENA accession number: ERS4285595), high-molecular weight DNA was extracted from the entire body of one F1 pupa using a QIAGEN Blood & Culture DNA Midi Kit (Qiagen, Germany) following the manufacturer's protocol, then library preparation and sequencing was performed by the Wellcome Sanger Institute (Cambridge, UK). A SMRTbell CLR (continuous long reads) sequencing library was constructed following the manufacturer's protocol, and sequenced on 5 SMRT (Single Molecule Real-Time) cells within a PacBio Sequel system (Pacific Biosciences, United States; RRID:SCR_017989) using version 3.0 chemistry and 10 hour runs. This generated 3,474,690 subreads, with a subread N50 of 18.8 kb and total of 39,471,717,610 bp. From the same sample, a 10X Genomics Chromium linked-read sequencing library (10X Genomics, United States) was also prepared following the manufacturer's protocol, and sequenced with 150 bp paired end reads on an Illumina HiSeq X Ten platform (Illumina, United States; RRID:SCR_016385). This generated 625,914,906 reads, and after mapping to the assembly described below, we estimate a barcoded molecule length of ~43 kbp.

Trio binning genome assembly

Canu version 1.8 (RRID:SCR_015880) [19] was used to bin *A. plantaginis* F1 offspring PacBio (Pacific Biosciences) subreads into those matching the paternal and maternal haplotypes defined by k-mers specific to the maternal and paternal Illumina data (Supplementary Figure 1). This resulted in 1,662,000 subreads assigned to the paternal haplotype, 1,529,779 subreads assigned to the maternal haplotype, and 2,445 (0.07%) subreads unassigned. Using only the assigned reads, the haplotype binned reads were assembled separately using wtdbg2 version 2.3 (RRID:SCR_017225) [20], with the '-xsq' pre-set option for PacBio Sequel data and an estimated genome size of 550Mb. The assemblies were polished using Arrow version 2.3.3 [21] and the haplotype binned PacBio reads. The 10X linked-reads were then used to scaffold each assembly using scaff10x [22], followed by another round of Arrow polishing on the scaffolds. To polish further with the 10X linked-read Illumina data, we first concatenated the two scaffolded assemblies, mapped the 10X Illumina data with Long Ranger version 2.2.0 [23] longranger align, called variants with freebayes version 1.3.1 [24], then applied homozygous non-reference edits to the assembly using bcftools consensus [25]. The assembly was then split back into paternal and maternal components, giving separate paternal haplotype (iArcPla.TrioW) and maternal haplotype (iArcPla.TrioY) assemblies.

Assembly contaminants were identified and removed by checking the assemblies against vector/adapter sequences in the UniVec database [26], common contaminants in eukaryotes [27] and organelle sequences [28, 29]. The assemblies were also checked against other organism sequences from the RefSeq database version 94 [30]. This identified mouse contamination in two scaffolds which were subsequently removed. The assemblies were

further manually assessed and corrected using gEVAL [31] with the available PacBio and 10X data. This process involved locating regions of zero or extreme PacBio read coverage and missed or mis-joins indicated by the 10X data, then evaluating the flagged discordances and correcting them where possible, which were typically missed joins, mis-joins and false duplications.

The Kmer Analysis Toolkit (KAT) version 2.4.2 [32] was used to compare k-mers from the 10X Illumina data to k-mers in each of the haplotype-resolved assemblies, and in the combined diploid assembly representing both haplotypes. For this analysis we used parameter K=21, which clearly identified error, haploid and diploid peaks for our dataset. Phasing of the assembled contigs and scaffolds was visualised using the parental k-mer databases produced by Canu [33]. To provide an estimate of assembly consensus accuracy, a quality value (QV) was computed for each assembly using Merqury version 1.0 [34]. Haploid genome size, heterozygosity and repeat fraction of the F1 offspring were estimated using GenomeScope (RRID:SCR_017014) [35] and k-mers derived from the 10X Illumina data. Assemblytics [36] was used to detect structural variants (SVs) between the parental haplotypes. For this, a whole-genome alignment was performed between the haplotype assemblies using the Nucmer module of MUMmer version 3.23 (MUMmer, RRID:SCR_018171) [37] with Assemblytics recommended options.

Comparative quality assessment

To assess the quality of each parental haplotype of the *A. plantaginis* trio binned assembly, standard contiguity metrics were computed, and assembly completeness was evaluated by calculating BUSCO (Benchmarking Universal Single-Copy Ortholog) scores using BUSCO version 3.0.2 (RRID:SCR_015008), comparing against the ‘insecta_odb9’ database of 1658

Insecta BUSCO genes with default Augustus (RRID:SCR_008417) parameters [38]. A quality comparison was conducted by comparing unscaffolded, Arrow polished versions of the trio binned assemblies against an unscaffolded, Arrow polished assembly of unbinned data from the same F1 offspring (iArcPla.wtdbg2). Quality comparisons were also performed for the final, scaffolded trio binned assemblies against a representative selection of published lepidopteran reference genomes, for which the latest versions of seven Lepidoptera species were downloaded: *Bicyclus anynana* version 1.2 [39], *Danaus plexippus* version 3 [40], *Heliconius melpomene* version Hmel.2.5 [41], *Manduca sexta* version Msex_1.0 [42] and *Melitaea cinxia* version MelCinx1.0 [43] were downloaded from Lepbase version 4.0 [12], whilst *Bombyx mori* version Bomo_genome_assembly [44] was downloaded from SilkBase version 2.1 [45] and *Trichoplusia ni* version PPHH01.1 [46] was downloaded from RefSeq version 94 [30]. Cumulative scaffold plots were visualised in R version 3.5.1 [47] using the ggplot2 package version 3.1.1 (RRID:SCR_014601) [48].

Genome annotation

Genome annotations were produced for each parental haplotype of the *A. plantaginis* trio binned assembly using the BRAKER2 version 2.1.3 pipeline [49]. A *de novo* library of repetitive sequences was identified with both genomes using RepeatScout version 1.0.5 (RRID:SCR_014653) [50]. Repetitive regions of the genomes were soft masked using RepeatMasker version 4.0.9 (RRID:SCR_012954) [51], Tandem Repeats Finder version 4.00 [52] and the RMBlast version 2.6.0 sequence search engine [53] combined with the Dfam_Consensus-20170127 database [54]. Raw RNA-seq reads were obtained from Galarza et al. 2017 [55] under study accession number PRJEB14172, which came from whole body tissue of *A. plantaginis* larvae from two families reared under two heat treatments. Using cutadapt version 1.8.1 (RRID:SCR_011841) [56], RNA-seq reads were trimmed for adapter

contamination and quality trimmed at both ends of each read using a quality value of 3 (-q 3,3). Quality control was performed pre and post trimming with fastqc version 0.11.8 [57]. RNA-seq reads were mapped to each respective genome using STAR (Spliced Transcripts Alignment to a Reference) version 2.7.1 [58]. Arthropod proteins were obtained from OrthoDB [59] and aligned to the genomes using GenomeThreader version 1.7.0 [60]. BRAKER2's *ab initio* gene predictions were carried out using homologous protein and *de novo* RNA-seq evidence using Augustus version 3.3.2 [49] and GeneMark-ET version 4.38 [49]. Annotation completeness was assessed using BUSCO version 3.0.2 against the 'insecta_odb9' database of 1658 Insecta BUSCO genes with default Augustus parameters [38].

Cytogenetic analysis

Spread chromosome preparations for cytogenetic analysis were produced from wing imaginal discs and gonads of third to fifth instar larvae, according to Šíchová et al. 2013 [61]. Female and male gDNA were extracted using the CTAB (hexadecyltrimethylammonium bromide) method, adapted from Winnepenninckx et al. 1993 [62]. These were used to generate probe and competitor DNA, respectively, for genomic *in situ* hybridization (GISH). Female genomic probe was labelled with Cy3-dUTP (cyanine 3-deoxyuridine triphosphate; Jena Bioscience, Germany) by nick translation, following Kato et al. 2006 [63] with a 3.5 hour incubation at 15°C. Male competitor DNA was fragmented with a 20 minute boil. GISH was performed following the protocol of Yoshido et al. 2005 [64]. For each slide, the hybridization cocktail contained 250 ng of female labelled probe, 2-3 µg of male competitor DNA, and 25 µg of salmon sperm DNA. Preparations were counterstained with 0.5 mg/ml DAPI (4',6-diamidino-2-phenylindole; Sigma-Aldrich) in DABCO antifade (1,4-diazabicyclo[2.2.2]octane; Sigma-Aldrich). Results were observed in the Zeiss Axioplan 2

Microscope (Carl Zeiss, Germany) and documented with an Olympus CCDMonochrome Camera XM10, with the cellSens 1.9 digital imaging software (Olympus Europa Holding, Germany). Images were pseudo-colored and superimposed in Adobe Photoshop CS3.

Population genomic analysis

We implemented the novel *A. plantaginis* reference assembly to analyse patterns of population genomic variation between 40 wild, adult males sampled from the European portion of *A. plantaginis'* Holarctic species range [18]. Samples were collected by netting and pheromone traps from Central Finnish (n=10) and Southern Finnish populations (n=10) where yellow and white morphs exist in equal proportions, an Estonian population (n=5) where white morphs are frequent compared to rare yellow morphs, a Scottish population (n=10) where only yellow morphs exist, and a Georgian population (n=5) where red morphs exist alongside yellow morphs (Figure 5A). Exact sampling localities are available in Supplementary Table 1. Whole genomic DNA extraction and short read sequencing was performed following the same method as described for short read sequencing of parental genomes during trio binning assembly. ENA accession numbers for all resequenced samples are available in Supplementary Table 2.

Reads were mapped against the paternal iArcPla.TrioW assembly (chosen due to higher assembly completeness; Table 2) using BWA-MEM (Burrows-Wheeler Aligner) version 7.17 [65] with default parameters, resulting in a mean sequencing coverage of 13X (Supplementary Table 2**)**. Alignments were sorted with SAMtools version 1.9 (RRID:SCR_002105) [66] and PCR-duplicates were removed with Picard version 2.18.15 (RRID:SCR_006525) [67]. Variants were called for each sample using Genome Analysis Tool Kit (GATK) HaplotypeCaller version 3.7 [68, 69], followed by joint genotyping across

all samples using GATK version 4.1 GenotypeGVCFs [68, 69], with expected heterozygosity set to 0.01. The raw SNP (single nucleotide polymorphism) callset was quality filtered by applying thresholds: quality by depth (QD>2.0), root mean square mapping quality (MQ>50.0), mapping quality rank sum test (MQRankSum>-12.5), read position rank sum test (ReadPosRankSum>-8.0), Fisher strand bias (FS<60.0) and strand odds ratio (SOR<3.0). Filters by depth (DP) of greater than half the mean (DP>409X) and less than double the mean (DP<1636X) were also applied. Linkage disequilibrium (LD) pruning was applied using the ldPruning.sh script [70] with an LD threshold of $r^2<0.01$, in 50kb windows shifting by 10kb. This callset was further filtered for probability of heterozygosity excess p-value>$1x10^{-5}$ using VCFtools version 0.1.15 (RRID:SCR_001235) [71] to exclude potential paralogous regions, giving our analysis-ready callset.

An unrooted, maximum likelihood (ML) phylogenetic tree was constructed to evaluate phylogenomic relationships, using our analysis-ready callset which was further reduced in size by subsampling every other SNP. The best-scoring ML tree was built in RAxML (Random Axelerated Maximum Likelihood) version 8.2.12 [72] with 100 rapid bootstrap replicates, using the GTRGAMMA model (generalised time-reversible substitution model and gamma model of rate heterogeneity) and Lewis ascertainment bias correction to account for the lack of monomorphic sites, then visualised in FigTree version 1.4.4 (RRID:SCR_008515) [73]. A principle component analysis (PCA) was also conducted to evaluate genome-wide population structure. A minor allele frequency filter of 0.05 was applied to our analysis-ready callset using VCFtools version 0.1.15 [71] to remove PCA-uninformative SNPs, then PCA was performed in R version 3.5.1 [47] using the SNPRelate package version 3.3 [74].

Results and Discussion

Trio binning genome assembly

K-mer spectra plots (Figure 2) indicate a highly complete assembly of both parental haplotypes in the *A. plantaginis* diploid offspring genome. There is good separation between the parental haplotypes, as each haploid assembly consists mostly of single-copy k-mers with low frequency of 2-copy k-mers, indicating a correctly haplotype-resolved assembly with low levels of artefactual duplication (Figure 2B, 2C; Supplementary Figure 2). This is also confirmed by the spectra plot for the combined diploid assembly (Figure 2A), where homozygous regions consist mostly of 2-copy k-mers and heterozygous regions consist mostly of 1-copy k-mers, as expected from the presence of both complete, parental haplotypes and low artefactual duplication. Using Merqury [34], we estimated QV scores of Q34.7 for the paternal (iArcPla.TrioW) assembly and Q34.2 for the maternal (iArcPla.TrioY) assembly, indicating high (>99.9%) assembly accuracy.

Using GenomeScope [35], we estimated the F1 offspring haploid genome size to be 590Mb with a repeat fraction of 27% and whole genome heterozygosity of ~1.9% (Supplementary Figure 3). This value was similar to our mean heterozygosity estimate of ~1.8% in a wild, Finnish population (Supplementary Table 4; method described in Supplementary Text 2), demonstrating our reference assembly is representative of natural variation in a wild population. The slight discrepancy may be explained by the parents used for trio binning assembly being derived from different selection lines, leading to greater heterozygosity between the trio binned parental haplotypes. Assemblytics [36] detected 32203 SVs between the haplotype assemblies, affecting 51.6 Mbp of the genome (Supplementary Table 5; Supplementary Figure 4). Successful haplotype separation was facilitated by the high estimated heterozygosity (~1.9%) of the F1 offspring genome, as it has previously been

discussed that higher heterozygosity makes trio binning easier [6]. Indeed, greater heterozygosity levels were obtained through our same-species *A. plantaginis* cross than obtained previously through same-species crosses for zebra finch (~1.6%) [9], *Arabidopsis* (~1.4%) [6], bovid (~0.9%) [6] and human (~0.1%) [6] trio binned assemblies, as well as an inter-species yak (*Bos grunniens)* x cattle (*Bos taurus)* cross (~1.2%) [7].

Genome annotation

We identified and masked 222,866,714 bp (41.04%) and 227,797,418 bp (42.80%) of repetitive regions in the iArcPla.TrioW and iArcPla.TrioY assemblies, respectively (Table 1). The BRAKER2 pipeline annotated a total of 19,899 protein coding genes in the soft-masked iArcPla.TrioW genome with 98.0% BUSCO completeness, whilst 18,894 protein coding genes were annotated in the soft-masked iArcPla.TrioY genome with 95.9% BUSCO completeness (Table 1).

Table 1. **Genome annotation statistics for the *Arctia plantaginis* trio binned assembly.** Statistics generated using the BRAKER2 pipeline, for the paternal (iArcPla.TrioW) and maternal (iArcPla.TrioY) haplotype assemblies.

		iArcPla.TrioW (paternal)	iArcPla.TrioY (maternal)
Total Genome size (bp)		584,621,344	577,993,050
Repetitive sequences (bp)		239,949,688	247,356,128
Masked repeats (%)		41.04	42.80
Mapped RNA-seq reads (n)		599,065,138	590,780,528
Mapped RNA-seq reads (%)		95.45	94.13
Protein-coding genes (n)		19,899	18,894
Mean gene length (bp)		5,966	5,951
BUSCO Completeness (%; n:1658)		98.00	95.90
Repeat Elements (n)	Total	11,320	12,576
	DNA Transposons	3,222	3,366
	LTR	1,891	2,192

	LINES	3,006	3,506
	SINES	544	547
	Unclassified	2,657	2,965

Comparative quality assessment

The paternal (iArcPla.TrioW) assembly contains 1069 scaffolds with N50=6.73 Mb and 98.1% complete BUSCOs, and the maternal (iArcPla.TrioY) assembly contains 1050 scaffolds with N50=9.77 Mb and 96.4% complete BUSCOs (Table 2). Prior to scaffolding work with 10X data, both unscaffolded trio binned assemblies are already more contiguous and complete than a composite haploid iArcPla.wtdbg2 assembly produced using unbinned data from the same individual (Table 2; Figure 3A). This illustrates the quality improvement achieved by separating haplotypes before assembly, and further improvement of the trio binned assemblies after scaffolding with 10X linked-reads (Table 2). The trio binned assemblies are also less inflated than the unbinned assembly with halved duplicated BUSCOs (Table 2; Figure 3A), suggesting a reduction in artefactual assembly duplication at heterozygous sites through read binning.

The trio binned *A. plantaginis* assemblies are of comparable quality to the best reference genomes available for Lepidoptera (Table 2; Figure 3B). When compared to other published lepidopteran reference genomes, quality of the *A. plantaginis* assemblies surpasses all but the best *Heliconius melpomene* [41] and *Bombyx mori* [44] assemblies (Table 2; Figure 3B). As contiguity of the *H. melpomene* assembly was improved through pedigree linkage mapping and haplotypic sequence merging [41], whilst bacterial artificial chromosome (BAC) and fosmid clones were used to close gaps in the *B. mori* assembly [44], it is impressive that trio binning has instantly propelled contiguity of the *A. plantaginis* genome to very near that of *H. melpomene* and *B. mori,* before incorporating information from any additional technologies.

Therefore, these comparisons strongly support trio binning as an effective strategy for *de novo* assembly of highly heterozygous genomes. Future scaffolding work has the potential to lead to a chromosomal-scale *A. plantaginis* assembly.

Table 2. Comparison of assembly contiguity and completeness between *Arctia plantaginis* and seven publicly available lepidopteran assemblies. Standard contiguity and BUSCO completeness metrics generated for each genome assembly, highlighting the high-quality *A. plantaginis* assembly achieved by trio binning. See **Figure 3** for assembly contiguity visualisation via cumulative scaffold plots, and **Supplementary Table 3** for the full BUSCO analysis summary.

	Assembly contiguity					Assembly completeness		
	Assembly size (Mb)	Total scaffolds/ contigs	Longest scaffold/ contig (Mb)	N50 (kb)	N50 count	Total complete BUSCOs	Single copy BUSCOs	Duplicated BUSCOs
Arctia plantaginis (binned: iArcPla.TrioW, scaffolded assembly)	585	1069	21.5	6730	24	98.1%	96.9%	1.2%
Arctia plantaginis (binned: iArcPla.TrioY, scaffolded assembly)	578	1050	24.4	9770	18	96.4%	95.3%	1.1%
Arctia plantaginis (binned: iArcPla.TrioW, unscaffolded assembly)	585	1441	11.4	2000	75	97.4%	96.4%	1.0%
Arctia plantaginis (binned: iArcPla.TrioY, unscaffolded assembly)	578	1290	23.8	4016	37	95.1%	94.1%	1.0%
Arctia plantaginis (unbinned: iArcPla.wtdbg2, unscaffolded assembly)	615	2948	11.3	1840	85	96.9%	94.8%	2.1%
Bicyclus anynana	475	10800	5.04	638.3	194	97.6%	96.8%	0.8%
Bombyx mori	482	696	21.5	16796	13	98.4%	97.2%	1.2%
Danaus plexippus	249	5397	6.24	715.6	101	98.0%	96.0%	2.0%
Heliconius melpomene	275	332	18.1	14308	9	97.7%	96.7%	1.0%

Manduca sexta	419	20871	3.25	664.0	169	96.7%	93.9%	2.8%
Melitaea cinxia	390	8261	0.668	119.3	970	83.0%	82.9%	0.1%
Trichoplusia ni	333	1916	8.93	4648	27	97.4%	96.6%	0.8%

Cytogenetic analysis

Mitotic nuclei prepared from wing imaginal discs of *A. plantaginis* larvae contained 2n=62 chromosomes in both sexes (Figure 4) in agreement with a previously reported modal chromosome number of arctiid moths [75], which is also the likely ancestral lepidopteran karyotype [43]. These insights will be helpful for future scaffolding work into a chromosomal-scale *A. plantaginis* reference assembly. Chromosomes decreased gradually in size, as is typical for lepidopteran karyotypes [76]. Due to the holokinetic nature of lepidopteran chromosomes, separation of sister chromatids by parallel disjunction was observed in mitotic metaphases [77]. Notably, two smallest chromosomes separated earlier compared to the other chromosomes (Figure 4A), although this could be an artefact of the spreading technique used for chromosome preparation. The presence of a W chromosome was confirmed in female nuclei by genomic *in situ* hybridization (Supplementary Figure 5; Supplementary Text 2).

Population genomic variation across the European range

As an empirical application of the *A. plantaginis* reference genome, we conducted a population resequencing analysis to describe genomic variation between 40 wild *A. plantaginis* males from five populations spread across Europe (Figure 5A). PCA revealed clear population structuring with individuals clustering geographically by country of origin (Figure 5B), in congruence with strongly supported phylogenomic groupings also by country of origin (Figure 6). Central and Southern Finnish individuals grouped into a single

population as expected from their geographic proximity (Figure 5B; Figure 6). The Finnish and Estonian populations clustered together away from the Scottish population along principle component (PC) 2 (Figure 5B) and on the phylogenetic tree (Figure 6), as would be predicted by effects of isolation by distance [78]. The Georgian population was highly genetically differentiated from all other sampled European populations, separating far along PC1 (Figure 5B) and possessing a much longer inter-population branch in the ML tree (Figure 6). Since the Georgian population has a distinctive genomic composition from the rest of the sampled distribution, this could support the hypothesis of incipient speciation in the Caucasus [18]. However, populations must be sampled in the large geographic gap between Georgia and the other populations in this preliminary analysis, to determine if genetic differentiation still persists when compared to nearby Central European populations.

Internal branch lengths were strikingly shorter within the Georgian population, indicating much higher intra-population relatedness than in populations outside of Georgia (Figure 6). This signal of low genetic variation within Georgia was unlikely caused by sampling relatives, as individuals were collected from a large population. Whilst further sampling is required to confirm whether the signal persists across the Caucasus, this finding casts doubt on the hypothesis that the *A. plantaginis* species originated in the Caucasus, which is based on morphological parsimony [18]. If *A. plantaginis* spread from the Caucasus with a narrow founder population, as suggested in Hegna et al. 2015 [18], we would expect higher genetic diversity in the Caucasus compared to the other geographic regions. Similar patterns of strong genetic differentiation and low genetic diversity in Caucasus and other European mountain ranges have been observed in the Holarctic butterfly *Boloria eunomia* [79], which likely retreated into refugia provided by warmer micro-habitats within European mountain ranges during particularly harsh glaciation periods. Perhaps a similar scenario occurred in *A.*

plantaginis, with founders of the Caucasus population restricted during severe glacial conditions. The species origin of *A. plantaginis* therefore remains unknown, and may be clarified by future inclusion of an *Arctia* outgroup to root the phylogenetic tree.

Conclusions

By converting heterozygosity into an asset rather than a hindrance, trio binning provides an effective solution for *de novo* assembly of heterozygous regions, with our high-quality *A. plantaginis* reference genome paving the way for the use of trio binning to successfully assemble other highly heterozygous genomes. As the first trio binned genome available for any invertebrate species, our *A. plantaginis* assembly adds support to trio binning as the best method for achieving fully haplotype-resolved, diploid genomes. Our assembly further highlights that trio binning can work well for a non-model system, provided a family trio can be obtained, which remains challenging for many non-model systems where it is difficult to obtain both parents and rear their offspring. Finally, the high-quality *A. plantaginis* reference assembly and annotation itself will contribute to Lepidoptera comparative phylogenomics by broadening taxonomic sampling into the Erebidae family, whilst facilitating genomic research on the *A. plantaginis* evolutionary study system.

Availability of supporting data

The trio binned assemblies, annotations and all raw sequencing data for *Arctia plantaginis* reported in this article are available under ENA study accession number PRJEB36595. All supporting data and materials are available in the *GigaScience* GigaDB database [80].

Additional files

Supplementary Figure 1: PacBio read length distribution for the *Arctia plantaginis* F1 offspring genome

Supplementary Figure 2: K-mer blob plot visualising haplotype specific k-mers for *Arctia plantaginis*

Supplementary Figure 3: GenomeScope profile of the *Arctia plantaginis* F1 offspring genome

Supplementary Figure 4: Comparison of structural variant sizes between the *Arctia plantaginis* trio binned haplotypes assemblies

Supplementary Figure 5: Cytogenetic analysis of *Arctia plantaginis* sex chromosomes

Supplementary Text 1: Results for cytogenetic analysis of *Arctia plantaginis* sex chromosomes

Supplementary Text 2: Method for estimating wild *Arctia plantaginis* genome heterozygosity

Supplementary Table 1: Exact sampling localities of wild *Arctia plantaginis* males used in population genomic analysis

Supplementary Table 2: Resequenced genome statistics for wild *Arctia plantaginis* males used in population genomic analysis

Supplementary Table 3: Full BUSCO summary for *Arctia plantaginis* and seven publicly available lepidopteran genome assemblies

Supplementary Table 4. Heterozygosity per male in the wild Finnish *Arctia plantaginis* population

Supplementary Table 5. Structural variant sizes present between the *Arctia plantaginis* trio binned haplotypes assemblies

DECLARATIONS

List of abbreviations

BAC: bacterial artificial chromosome, bp: base pairs, BUSCO: Benchmarking Universal Single-Copy Ortholog, BWA: Burrows-Wheeler Aligner, CLR: continuous long reads, CTAB: hexadecyltrimethylammonium bromide, Cy3-dUTP: cyanine 3-deoxyuridine triphosphate, DABCO: 1,4- diazabicyclo[2.2.2]octane, DAPI: 1,4- diazabicyclo[2.2.2]octane, ENA: European Nucleotide Archive, FS: Fisher strand bias, GATK: Genome Analysis Tool Kit, GISH: genomic *in situ* hybridization, GTRGAMMA: generalised time-reversible substitution model and gamma model of rate heterogeneity, KAT: Kmer Analysis Toolkit, kbp: kilobase pairs, LD: linkage disequilibrium, Mbp: megabase pairs, ml: millilitre, ML: maximum likelihood, MQ: root mean square mapping quality, MQRankSum: mapping quality rank sum test, PacBio: Pacific Biosciences, PC: principle component, PCA: principle component analysis, QD: quality by depth, QV: quality value, RAxML: Random Axelerated Maximum Likelihood, ReadPosRankSum: read position rank sum test, SMRT: Single Molecule, Real-Time, SNP: single nucleotide polymorphism, SOR: strand odds ratio, STAR: Spliced Transcripts Alignment to a Reference, SV: structural variant

Ethics approval and consent to participate

Not applicable.

Consent for publication

Not applicable.

Competing interests

The authors declare that they have no competing interests.

Funding

CDJ, ECY, TNG, JIM and IAW were supported by ERC Speciation Genetics Advanced Grant 339873 to perform DNA extraction, sequencing and genome annotation and population genomic analysis. SAM and RD were supported by Wellcome grant WT207492 to perform genome assembly. SP was supported by Wellcome grant WT206194 to perform genome curation. JAG and JM were supported by the Academy of Finland (project number 320438 and 328474) and the University of Jyväskylä to perform family rearing and fieldwork. PN was supported by the grant of Czech Science Foundation reg. no. 20-20650Y to perform cytogenetic analysis.

Author contributions

CDJ conceived and provided funding for the study. JAG and JM performed rearing and fieldwork, for which JM provided the funding. ECY and IAW performed genomic extractions. SAM performed genome assembly, for which RD provided funding. SP performed genome curation. TNG performed genome annotation. PN performed cytogenetic analysis. ECY performed comparative quality assessment. ECY performed population genomic analysis, with contributions from JIM. ECY, SAM and PN produced figures. ECY wrote the manuscript with contributions from JAG, SAM, TNG and PN, and input from all authors.

Acknowledgements

We thank Kaisa Suisto for assisting in sample rearing, and we thank the Agency of Protected Areas of Georgia for granting us access to perform fieldwork. We also thank Novogene (China) for performing Illumina whole genome library preparation and sequencing, and the Wellcome Sanger Institute (Cambridge, UK) for performing PacBio and 10X Chromium library preparation and sequencing.

REFERENCES

1. Ellegren H. Genome sequencing and population genomics in non-model organisms. Trends in Ecology & Evolution. 2013; 29: 51–63.

2. Jayakumar V, Sakakibara Y. Comprehensive evaluation of non-hybrid genome assembly tools for third-generation PacBio long-read sequence data. Brief Bioinform. 2017; 20: 866–876.

3. Vinson JP, Jaffe DB, O'Neill K, et al. Assembly of polymorphic genomes: Algorithms and application to *Ciona savignyi*. Genome Res. 2005; 15: 1127–1135.

4. Pryszcz LP, Gabaldón T. Redundans: an assembly pipeline for highly heterozygous genomes. Nucleic Acids Res. 2016; doi:10.1093/nar/gkw294.

5. Garg S, Rautiainen M, Novak AM, et al. A graph-based approach to diploid genome assembly. Bioinformatics. 2018; 34: i105–i114.

6. Koren S, Rhie A, Walenz BP, et al. *De novo* assembly of haplotype-resolved genomes with trio binning. Nature Biotechnology. 2018; 36: 1174–1182.

7. Rice ES, Koren S, Rhie A, et al. Chromosome-length haplotigs for yak and cattle from trio binning assembly of an F1 hybrid. GigaScience. 2020; doi:10.1093/gigascience/giaa029

8. Rönkä K, Mappes J, Kaila L, et al. Putting Parasemia in its phylogenetic place: a molecular analysis of the subtribe Arctiina (Lepidoptera). Systematic Entomology. 2016; 41: 844–853.

9. Kronenberg ZN, Rhie A, Koren S, et al. Extended haplotype phasing of de novo genome assemblies with FALCON-Phase. BioRxiv. 2019; doi: https://doi.org/10.1101/327064.

10. Wenger AM, Peluso P, Rowell WJ, et al. Accurate circular consensus long-read sequencing improves variant detection and assembly of a human genome. Nature Biotechnology. 2019; 37: 1155–1162.

11. Vertebrate Genomes Project GenomeArk. https://vgp.github.io/genomeark. Accessed May 2020.

12. Challis RJ, Kumar S, Dasmahapatra KK, et al. Lepbase: the Lepidopteran genome database. BioRxiv. 2016; doi: https://doi.org/10.1101/056994. URL: download.lepbase.org. Accessed July 2019.

13. Kawahara AY, Breinholt JW. Phylogenomics provides strong evidence for relationships of butterflies and moths. Proceedings of the Royal Society B: Biological Sciences. 2014; 281: 20140970.

14. Breinholt JW, Earl C, Lemmon AR, et al. Resolving Relationships among the Megadiverse Butterflies and Moths with a Novel Pipeline for Anchored Phylogenomics. Syst Biol. 2018; 67: 78–93.

15. Triant DA, Cinel SD, Kawahara AY. Lepidoptera genomes: current knowledge, gaps and future directions. Current Opinion in Insect Science. 2018; 25: 99–105.

16. Lindstedt C, Eager H, Ihalainen E, et al. Direction and strength of selection by predators for the color of the aposematic wood tiger moth. Behav Ecol. 2011; 22: 580–587.

17. Galarza JA, Nokelainen O, Ashrafi R, et al. Temporal relationship between genetic and warning signal variation in the aposematic wood tiger moth (*Parasemia plantaginis*). Molecular Ecology. 2014; 23: 4939–4957.

18. Hegna RH, Galarza JA, Mappes J. Global phylogeography and geographical variation in warning coloration of the wood tiger moth (*Parasemia plantaginis*). Journal of Biogeography. 2015; 42: 1469–1481.

19. Koren S, Walenz BP, Berlin K, et al. Canu: scalable and accurate long-read assembly via adaptive k-mer weighting and repeat separation. Genome Research. 2017; 27: 722-736.

20. Ruan J, Li H. Fast and accurate long-read assembly with wtdbg2. Nat Methods. 2019; 17: 155-8.

21. GenomicConsensus. https://github.com/PacificBiosciences/GenomicConsensus. Accessed March 2019.

22. Scaff10X. https://github.com/wtsi-hpag/Scaff10X. Accessed March 2019.

23. Long Ranger. https://github.com/10XGenomics/longranger. Accessed March 2019.

24. Garrison E, Marth G. Haplotype-based variant detection from short-read sequencing. arXiv. 2012; preprint arXiv:1207.3907 [q-bio.GN].

25. Freebayes-polish. https://github.com/VGP/vgp-assembly/tree/master/pipeline/freebayes-polish. Accessed March 2019.

26. UniVec Database. NCBI. https://www.ncbi.nlm.nih.gov/tools/vecscreen/univec. Accessed March 2019.

27. Contam_in_euks.fa.gz. ftp://ftp.ncbi.nlm.nih.gov/pub/kitts/contam_in_euks.fa.gz. Accessed March 2019.

28. Mito.nt.gz. ftp://ftp.ncbi.nlm.nih.gov/blast/db/ FASTA/mito.nt.gz. Accessed March 2019.

29. RefSeq Plastid Database. NCBI. ftp://ftp.ncbi.nlm.nih.gov/refseq/release/plastid. Accessed March 2019.

30. RefSeq: NCBI Reference Sequence Database. www.ncbi.nlm.nih.gov/refseq. Accessed March 2019.

31. Chow W, Brugger K, Caccamo M, et al. gEVAL — a web-based browser for evaluating genome assemblies. Bioinformatics. 2016; 32: 2508–2510.

32. Mapleson D, Accinelli GG, Kettleborough G, et al. KAT: A K-mer Analysis Toolkit to quality control NGS datasets and genome assemblies. Bioinformatics. 2016; doi: 10.1093/bioinformatics/btw663.

33. TrioBinning. https://github.com/arangrhie/TrioBinning. Accessed March 2019.

34. Rhie A, Walenz BP, Koren S et al. Merqury: reference-free quality and phasing assessment for genome assemblies, BioRxiv. 2020; doi: https://doi.org/10.1101/2020.03.15.992941.

35. Vurture GW, Sedlazeck FJ, Nattestad M, et al. GenomeScope: fast reference-free genome profiling from short reads. Bioinformatics. 2017; doi: https://doi.org/10.1093/bioinformatics/btx153.

36. Nattestad M, Schatz MC. Assemblytics: a web analytics tool for the detection of variants from an assembly. Bioinformatics. 2016; 32: 3021-3023.

37. Kurtz S, Phillippy A, Delcher AL et al. Versatile and open software for comparing large genomes. Genome Biology. 2004; 5: R12.

38. Simão FA, Waterhouse RM, Ioannidis P, et al. BUSCO: assessing genome assembly and annotation completeness with single-copy orthologs. Bioinformatics. 2015; 31: 3210–3212.

39. Nowell RW, Elsworth B, Oostra V, et al. A high-coverage draft genome of the mycalesine butterfly *Bicyclus anynana*. Gigascience. 2017; 6: 1–7.

40. Zhan S, Reppert SM. MonarchBase: the monarch butterfly genome database. Nucleic Acids Res. 2013; 41: D758–D763.

41. Davey JW, Chouteau M, Barker SL, et al. Major Improvements to the *Heliconius melpomene* Genome Assembly Used to Confirm 10 Chromosome Fusion Events in 6 Million Years of Butterfly Evolution. G3 (Bethesda). 2016; 6: 695–708.

42. Kanost MR, Arrese EL, Cao X, et al. Multifaceted biological insights from a draft genome sequence of the tobacco hornworm moth, *Manduca sexta*. Insect Biochem Mol Biol. 2016; 76: 118–147.

43. Ahola V, Lehtonen R, Somervuo P, et al. The Glanville fritillary genome retains an ancient karyotype and reveals selective chromosomal fusions in Lepidoptera. Nature Communications. 2014; 5: 4737.

44. Kawamoto M, Jouraku A, Toyoda A, et al. High-quality genome assembly of the silkworm, *Bombyx mori*. Insect Biochemistry and Molecular Biology. 2019; 107: 53–62.

45. SilkBase. silkbase.ab.a.u- tokyo.ac.jp/cgi-bin/download.cgi. Accessed June 2019.

46. Chen W, Yang X, Tetreau G, et al. A high-quality chromosome-level genome assembly of a generalist herbivore, *Trichoplusia ni*. Molecular Ecology Resources. 2019; 19: 485–496.

47. R Core Team (2018). R: A language and environment for statistical computing. R Foundation for Statistical Computing, Vienna, Austria. URL https://www.R-project.org/.

48. Wickham H. ggplot2: Elegant Graphics for Data Analysis. Springer-Verlag New York. 2016.

49. Hoff KJ, Lange S, Lomsadze A, et al. BRAKER1: Unsupervised RNA-Seq-Based Genome Annotation with GeneMark-ET and AUGUSTUS. Bioinformatics. 2016; 32: 767–769.

50. Price AL, Jones NC, Pevzner PA. De novo identification of repeat families in large genomes. Bioinformatics. 2005; 21: i351–i358.

51. Smit AFA, Hubley R, Green P. *RepeatMasker Open-4.0*. 2013-2015; URL: http://www.repeatmasker.org. Accessed June 2019.

52. Benson G. Tandem repeats finder: a program to analyze DNA sequences. Nucleic Acids Res. 1999; 27: 573–580.

53. RMBlast. http://www.repeatmasker.org/RMBlast.html. Accessed June 2019.

54. Hubley R, Finn RD, Clements J, et al. The Dfam database of repetitive DNA families. Nucleic Acids Res. 2016; 44: D81–D89. URL: http://www.repeatmasker.org/libraries. Accessed June 2019.

55. Galarza JA, Dhaygude K, Mappes J. (2017). *De novo* transcriptome assembly and its annotation for the aposematic wood tiger moth (*Parasemia plantaginis*). Genomics Data. 2017; 12: 71–73.

56. Martin M. Cutadapt removes adapter sequences from high-throughput sequencing reads. EMBnet.Journal. 2011; 17: 10–12.

57. Andrews S. FASTQC. A quality control tool for high throughput sequence data. 2010; URL: http://www.bioinformatics.babraham.ac.uk/projects/fastqc/. Accessed June 2019.

58. Dobin A, Davis CA, Schlesinger F, et al. STAR: ultrafast universal RNA-seq aligner. Bioinformatics. 2013; 29: 15–21.

59. Kriventseva EV, Kuznetsov D, Tegenfeldt F, et al. OrthoDB v10: sampling the diversity of animal, plant, fungal, protist, bacterial and viral genomes for evolutionary and functional annotations of orthologs. Nucleic Acids Res. 2019; 47: D807–D811.

60. *GenomeThreader* Gene Prediction Software. genomethreader.org. Accessed June 2019.

61. Šíchová J, Nguyen P, Dalíková M, et al. Chromosomal Evolution in Tortricid Moths: Conserved Karyotypes with Diverged Features. PLOS ONE. 2013; 8: e64520.

62. Winnepenninckx B, Backeljau T, De Wachter R. Extraction of high molecular weight DNA from molluscs. Trends Genet. 1993; 9: 407.

63. Kato A, Albert PS, Birchler JA. Sensitive fluorescence in situ hybridization signal detection in maize using directly labeled probes produced by high concentration DNA polymerase nick translation. Biotechnic & Histochemistry. 2006; 81: 71–78.

64. Yoshido A, Marec F, Sahara K. Resolution of sex chromosome constitution by genomic in situ hybridization and fluorescence in situ hybridization with (TTAGG)(n) telomeric probe in some species of Lepidoptera. Chromosoma. 2005; 114: 193–202.

65. Li H. Aligning sequence reads, clone sequences and assembly contigs with BWA-MEM. ArXiv. 2013; ArXiv:1303.3997 [q-Bio].

66. Li H, Handsaker B, Wysoker A, et al. The Sequence Alignment/Map format and SAMtools. Bioinformatics. 2009; 25: 2078–2079.

67. Picard. broadinstitute.github.io/picard. Accessed October 2019.

68. McKenna A, Hanna M, Banks E, et al. The Genome Analysis Toolkit: A MapReduce framework for analyzing next-generation DNA sequencing data. Genome Res. 2010; 20: 1297–1303.

69. Poplin R, Ruano-Rubio V, DePristo MA, et al. Scaling accurate genetic variant discovery to tens of thousands of samples. BioRxiv. 2017; doi: https://doi.org/10.1101/201178.

70. joanam scripts. https://github.com/joanam/scripts/blob/master/ldPruning.sh. Accessed November 2019.

71. Danecek P, Auton A, Abecasis G, et al. The variant call format and VCFtools. Bioinformatics. 2011; 27: 2156–2158.

72. Stamatakis A. RAxML version 8: a tool for phylogenetic analysis and post-analysis of large phylogenies. Bioinformatics. 2014; 30: 1312–1313.

73. Rambaut A. FigTree version 1.4.3. 2014; URL: http://tree.bio.ed.ac.uk/software/figtree/. Accessed November 2019.

74. Zheng X, Levine D, Shen J, et al. A high-performance computing toolset for relatedness and principal component analysis of SNP data. Bioinformatics. 2012; 28: 3326–3328.

75. Robinson R. Lepidoptera Genetics. 1st ed. Oxford: Pergamon Press; 1971.

76. De Prins J, Saitoh K. Lepidoptera, Moths and Butterflies. In: Kristensen NP, editors. Handbook of Zoology. Berlin & New York: Walter de Gruyter; 2003. p. 449-468.

77. Murakami A, Imai HT. Cytological evidence for holocentric chromosomes of the silkworms, Bombyx mori and B. mandarina, (Bombycidae, Lepidoptera). Chromosoma. 1974; 47: 167–178.

78. Aguillon SM, Fitzpatrick JW, Bowman R, et al. Deconstructing isolation-by-distance: The genomic consequences of limited dispersal. PLoS Genet. 2017; 13: e1006911.

79. Maresova J, Habel JC, Neve G, et al. Cross-continental phylogeography of two Holarctic Nymphalid butterflies, *Boloria eunomia* and *Boloria selene*. PLOS ONE. 2019; 14: e0214483.

80. Yen EC; McCarthy SA; Galarza JA; Generalovic TN; Pelan S; Nguyen P; Meier JI; Warren IA; Mappes J; Durbin R; Jiggins CD. Supporting data for "A haplotype-resolved, de novo genome assembly for the wood tiger moth (*Arctia plantaginis*) through trio binning" GigaScience Database.2020; http://dx.doi.org/10.5524/100774.

FIGURE LEGENDS

Figure 1. Discrete colour morphs of *Arctia plantaginis* males. Whilst forewings remain white, hindwings are polymorphic with variable black patterns, existing as discrete (**A**) yellow (**B**) white and (**C**) red morphs, which can only be found in the Caucasus region. **(A-C)** show pinned dead morphs. **(D-E)** show examples of morphs in the wild. Photos: Johanna Mappes and Ossi Nokelainen.

Figure 2. **K-mer spectra plots for the *Arctia plantaginis* trio binned genome assembly.** Plots produced using K-mer Analysis Toolkit (KAT), showing the frequency of k-mers in an assembly versus the frequency of k-mers (i.e. sequencing coverage) in the raw 10X Illumina

reads, for the **(A)** combined diploid assembly (paternal plus maternal), **(B)** paternal-only assembly (iArcPla.TrioW), and **(C)** maternal-only assembly (iArcPla.TrioY). Colours represent k-mer copy number in the assembly: black k-mers are not represented (0-copy), red k-mers are represented once (1-copy), purple k-mers are represented twice (2-copy) and green k-mers are represented thrice (3-copy). The first peak corresponds to k-mers present in the raw reads but missing from the assembly due to sequencing errors, the second peak corresponds to k-mers from heterozygous regions, and the third peak corresponds to k-mers from homozygous regions. These plots show a complete and well-separated assembly of both haplotypes in the F1 offspring diploid genome.

Figure 3. Cumulative scaffold plots visualise the high assembly contiguity of the trio binned *Arctia plantaginis* genome. A highly contiguous assembly is represented by a near vertical line with a short horizontal tail of trailing tiny scaffolds. **(A)** Comparison of the unscaffolded *A. plantaginis* trio binned assemblies iArcPla.TrioW (paternal haplotype) and iArcPla.TrioY (maternal haplotype) against the unscaffolded composite assembly using unbinned data from the same individual (iArcPla.wtdbg2). The much steeper curve and shorter horizontal tail for the trio binned assemblies compared to the unbinned assembly shows that trio binning greatly improved contiguity. **(B)** Comparison of the *A. plantaginis* trio binned assemblies against a representative selection of published lepidopteran genomes, shown up to the first 10000 scaffolds. This comparison demonstrates that the *A. plantaginis* trio binned assemblies are much more contiguous than most other lepidopteran genomes currently available.

Figure 4. Cytogenetic analysis reveals 31 chromosomes in the *Arctia plantaginis* haploid genome. Chromosomes were counterstained with DAPI (blue). **(A)** Male mitotic metaphase

consisted of 2n=62 chromosomes. Note separated chromatids of the smallest chromosome pair (arrowheads). **(B)** Female mitotic complement consisted of 2n=62 elements. Scale bar=5 µm.

Figure 5. Sampling locations and population structure across *Arctia plantaginis'* European geographic range. **(A)** Sampling locations of 40 wild *A. plantaginis* males from the European portion of the Holarctic species range (see **Supplementary Table 1** for exact sampling coordinates). Circle size represents sample size (Central Finland: n=10, Estonia: n=5, Scotland: n=10, Southern Finland: n=10, Georgia: n=5), and circle colour indicates the proportion of each hindwing colour morph collected. **(B)** Genome-wide PCA (n=40; 752303 SNPs) with principle component 1 plotted against principle component 2, explaining 7.22% and 5.88% of total genetic variance, respectively.

Figure 6. Maximum likelihood unrooted phylogeny of wild *Arctia plantaginis* males (n=40) from the European geographic range. Tree constructed using RAxML with 100 rapid bootstraps, using 558549 SNPs. Node labels indicate bootstrap support. See **Figure 5A** for sampling locations.

Figure 1

Click here to access/download;Figure;Figure1_photos.png

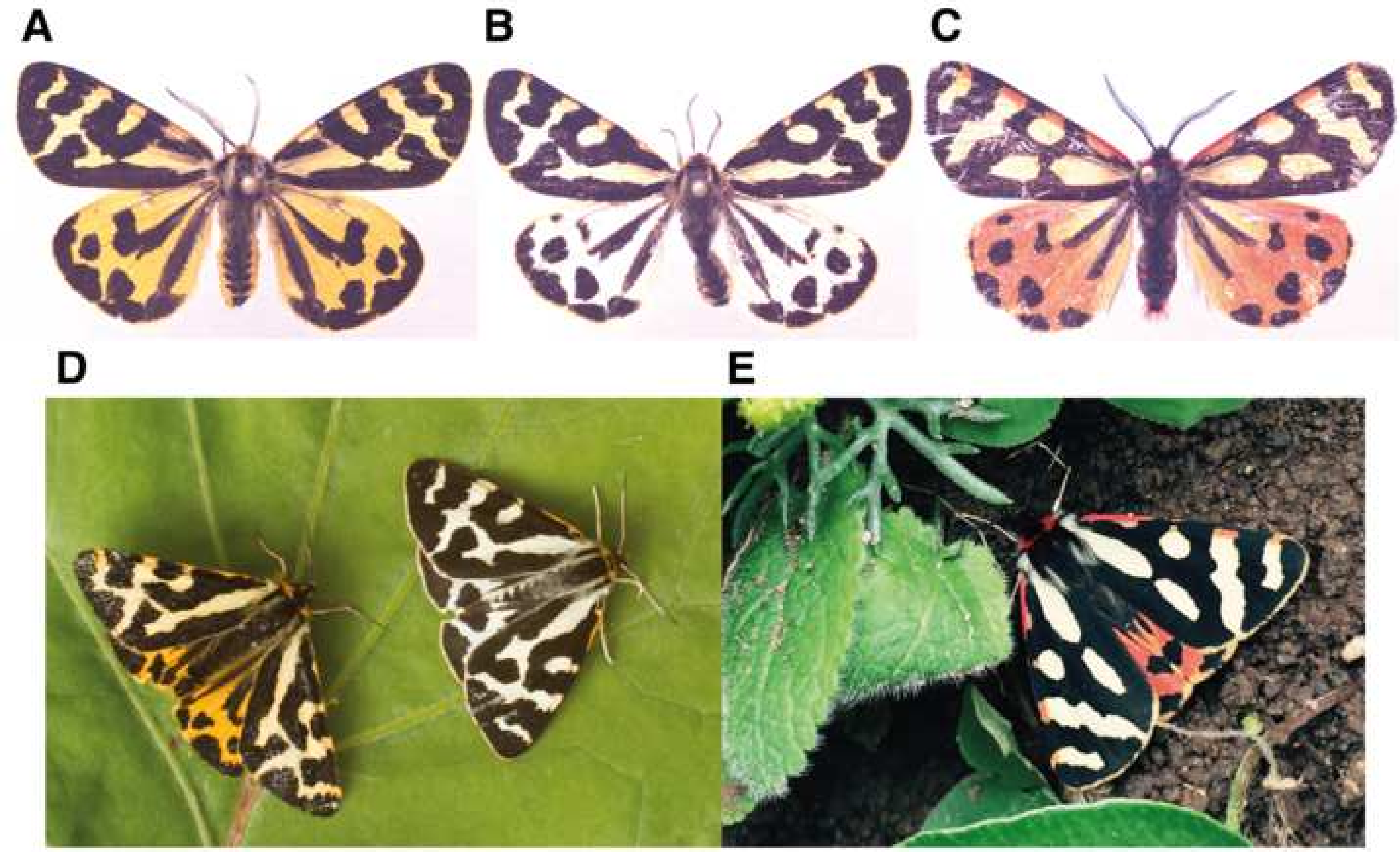

Figure 2

Click here to access/download;Figure;Figure2_kmerspectra.png

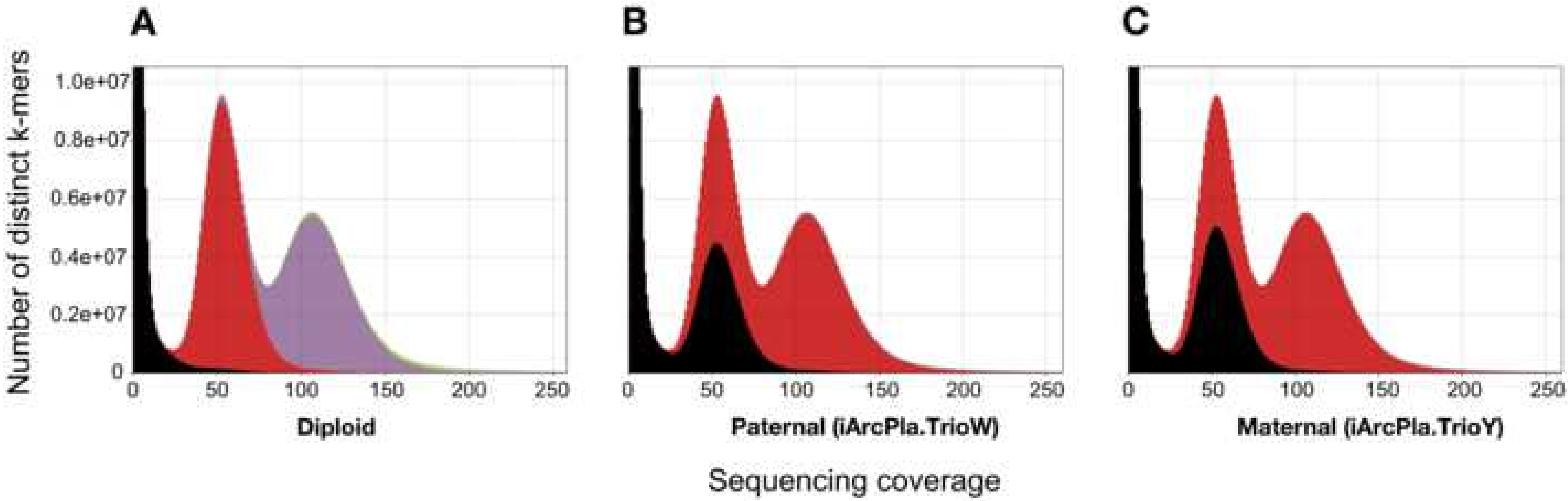

Figure 3

Click here to access/download;Figure;REVISED_Figure3_cumscaffplots.png

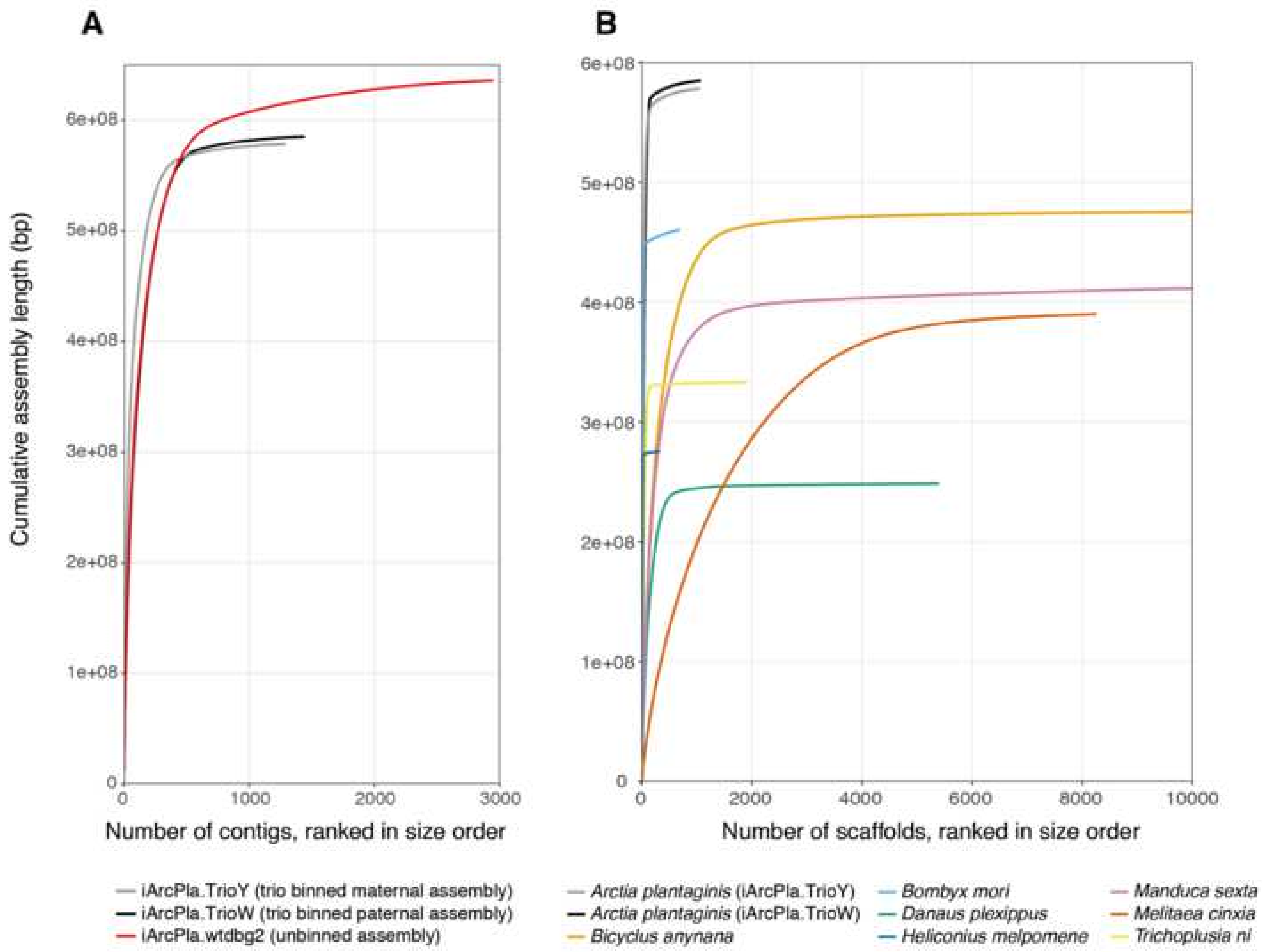

Figure 4

Click here to access/download;Figure;Figure4_cytogenetic.png

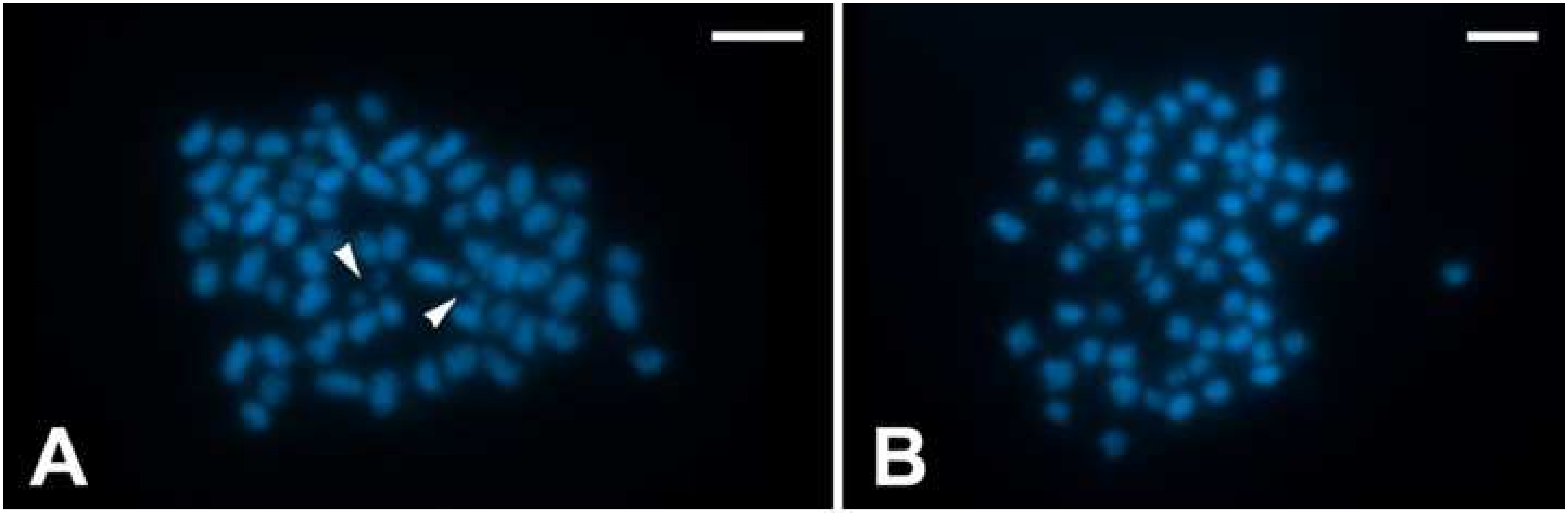

Figure 5

Click here to access/download;Figure;Figure5_map_PCA.png

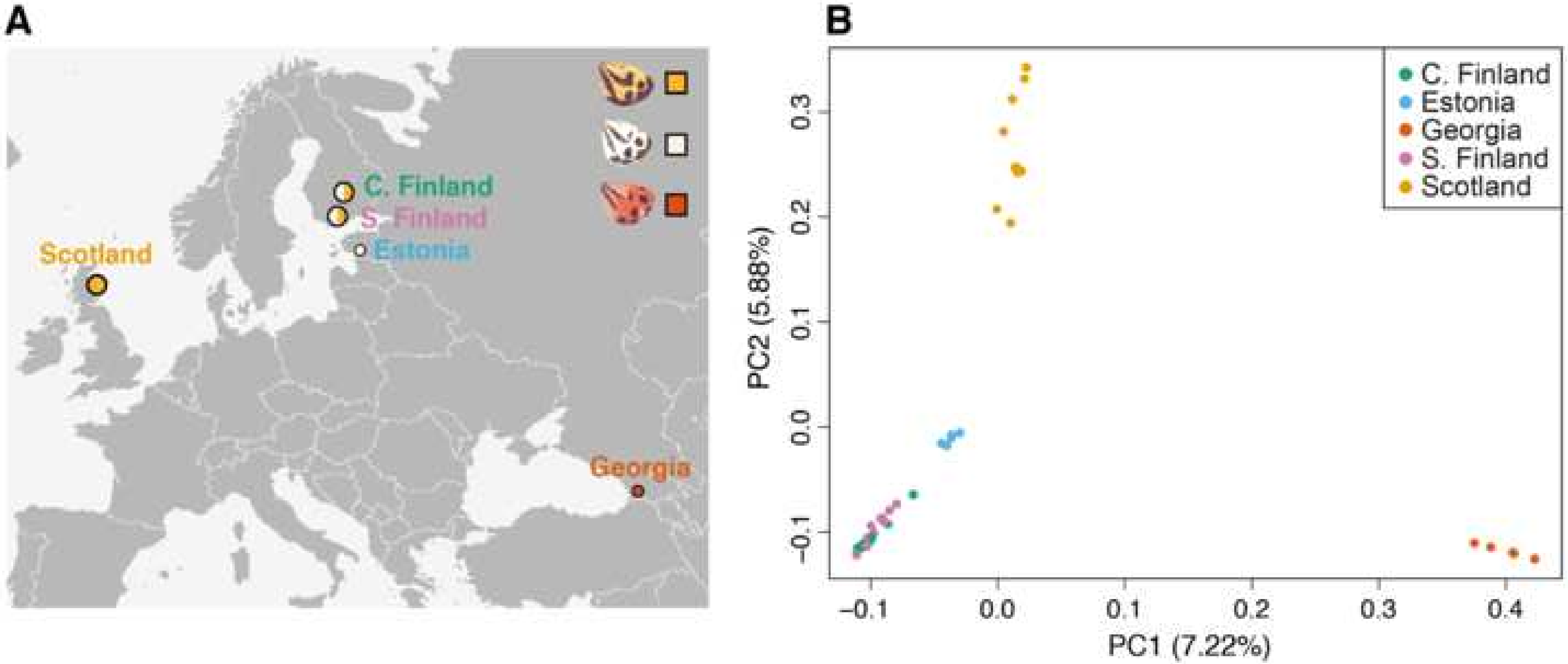

Figure 6 Click here to access/download;Figure;Figure6_RAxML.png

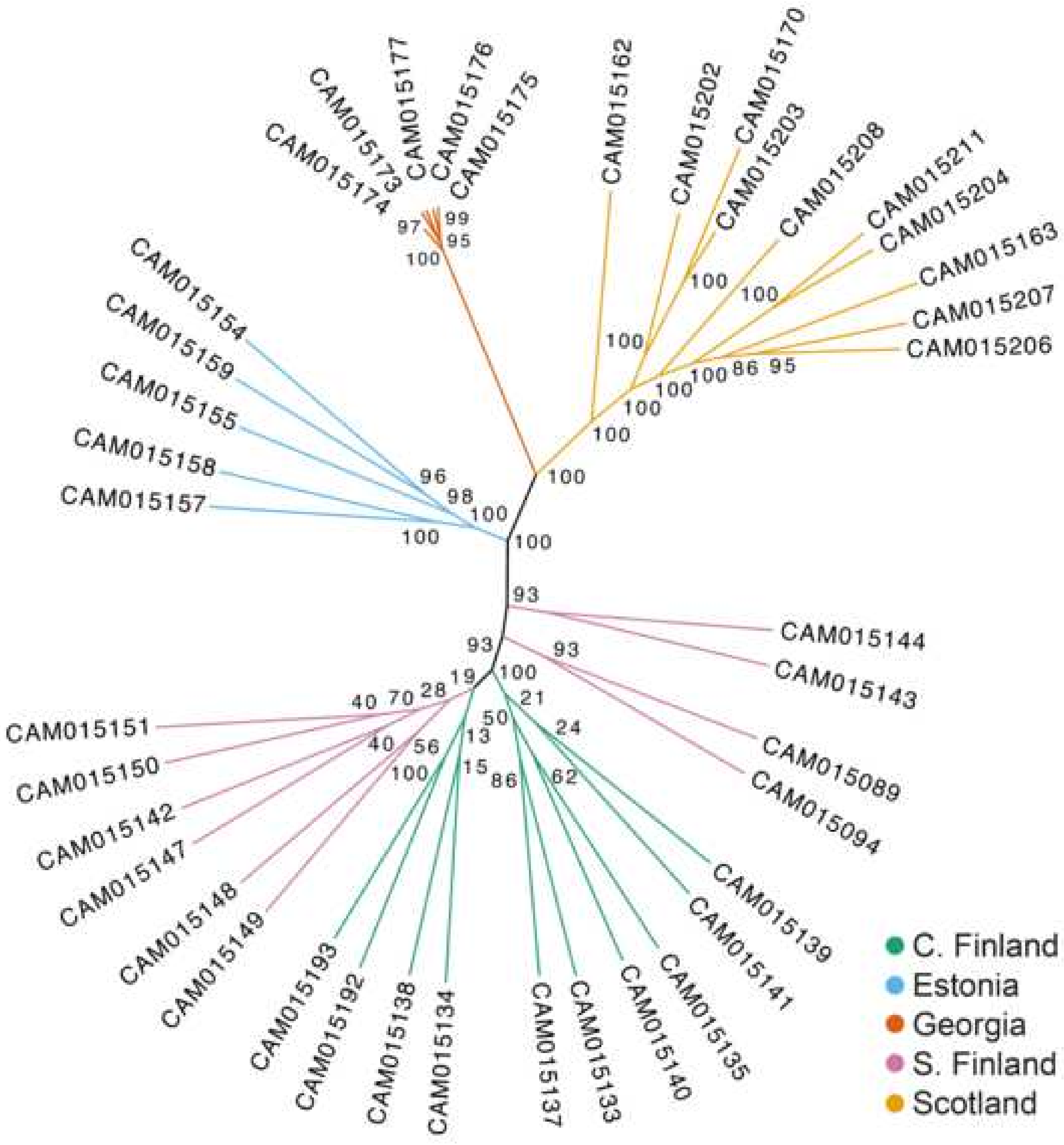

Click here to access/download

Supplementary Material

Yen Supplement Revision Tigermoth Triobinning.docx

Cover Letter and Response to Reviewers Click here to access/download;Personal Cover;Yen Response to Reviewers.docx

Manuscript GIGA-D-20-00060
Response to Reviewers

Dear Editor and Reviewers,

The authors of this manuscript thank you for providing us with the opportunity to submit a revised version of our manuscript **"A haplotype-resolved, *de novo* genome assembly for the wood tiger moth (*Arctia plantaginis*) through trio binning"**. We thank you very much for your time and effort dedicated to providing feedback on our manuscript, and we appreciate your insightful comments which have greatly improved our manuscript.

We have addressed all of the raised points and implemented the suggested minor revisions in our manuscript accordingly. Please see below for the reviewers' comments with our detailed point-by-point response in blue, with quotations from our original and revised manuscript italicised and indented. All page numbers refer to the revised manuscript file. We have also corrected minor errors in BUSCO values reported for the unbinned *Arctia plantaginis* and *Heliconius melpomene* assemblies, and updated the statement for "Availability of supporting data", as we have now populated our ENA accession number with the *A. plantaginis* trio binned assemblies and annotations.

We hope that our revised manuscript is acceptable for publication in *GigaScience*, and we look forward to hearing from you again.

Sincerely,

Eugenie C. Yen

Reviewer #1: Yen and colleagues present a haplotype-resolved draft assembly of the wood tiger moth genome using a trio-binning approach to leverage heterozygosity, a component of genome biology that is typically considered a confounding factor in the quest for a high quality assembly.
The manuscript is clearly structured and well-written. The primary data and assembly strategy are well-described with comprehensive and appropriate inclusion of methods, software versions and parameters. I can confirm that the ENA accession number is active and populated with the appropriate raw data. The assembly itself appears to be of excellent quality (in terms of completeness and contiguity) and the comparisons to a composite haplotype assembly from the same primary reads as well as to other lepidopteran genomes are highly relevant. Karyotypic analysis, presented here alongside the assembly, will be a useful point of reference for future scaffolding efforts. Finally, the authors demonstrate an application of the genome with a preliminary survey and population genomics analysis of 5 populations sampled across Europe and are appropriately cautious in the conclusions they draw from this analysis.
The work described here thoughtfully presents an accomplished assembly, with an approach that should be of broad interest, constitutes an important resource for lepidopteran biology and which anticipates the movement of the genome assembly field towards full diploid reconstruction. I have only minor comments and suggestions, which are set out below.

General
The resolution of the figures in the main submission, but not the supplement, is a little poor in the review copy.

We checked the resolution of our submitted figures, and we determine this issue should be specific to the reviewer copy only.

Background
While I agree that full diploid reconstruction is/should be a eukaryotic genome assembly target and that there are few published examples, it might be worth also noting that the Vertebrate Genome Project contains, I believe, 12 trio-based assemblies that are publicly accessible.

We thank the reviewer for pointing this out, and we agree that the mentioned assemblies should be included. We have counted the number of trio-based assemblies currently available on the VGP GenomeArk data and changed the sentence on page 4 accordingly:

> *This represents the first trio binned assembly available for Insecta and indeed any invertebrate animal species, diversifying the organisms for which trio binning has been applied outside of bovids [6, 7], zebra finches [9], humans [6, 9, 10] and Arabidopsis thaliana [6].*

to:

> *At the time of writing, this represents the first trio binned assembly available for an invertebrate animal species, diversifying the organisms for which published trio*

binned assemblies exist beyond bovids [6, 7], zebra finches [9], humans [6, 9, 10], Arabidopsis thaliana [6] and additional trio binned assemblies available for eight other vertebrate species on the Vertebrate Genomes Project GenomeArk database [11].

with added reference:

11. Vertebrate Genomes Project GenomeArk. https://vgp.github.io/genomeark. Accessed May 2020.

Methods
Please confirm that you have not done any of the following (and if you have, please incorporate details in the methods)
Any additional quality trimming of RNAseq reads beyond adaptor removal with cutadapt?

We have added the suggested details on page 9 by changing:

RNA-seq reads were trimmed for adapter contamination using cutadapt version 1.8.1 [48] and quality controlled pre and post trimming with fastqc version 0.11.8 [49].

to:

Using cutadapt version 1.8.1 [56], RNA-seq reads were trimmed for adapter contamination and quality trimmed at both ends of each read using a quality value of 3 (-q 3,3). Quality control was performed pre and post trimming with fastqc version 0.11.8 [57].

Any pre-processing of PacBio reads to remove adaptor contamination etc?

We confirm that there was no pre-processing of PacBio reads. We performed adapter contamination removal during the assembly curation stage, for which details have been added in our response to the reviewer comment “Can you elaborate further on the types of artefact/contamination/manual curation that was flagged by gEval?”

Please consider also calculating and reporting QV to provide an estimate of assembly accuracy (presenting figures before and after polishing with the 10x reads would be of interest).

We thank the reviewer for this suggestion and agree that reporting QV is useful. We have included a QV analysis in our revised manuscript. We have added a sentence describing the method on page 8:

To provide an estimate of assembly consensus accuracy, a quality value (QV) was computed for each assembly using Merqury version 1.0 [34].

and added a sentence describing the results on page 12-13:

Using Merqury [34], we estimated QV scores of Q34.7 for the paternal (iArcPla.TrioW) assembly and Q34.2 for the maternal (iArcPla.TrioY) assembly, indicating high (>99.9%) assembly accuracy.

with added reference:

34. Rhie A, Walenz BP, Koren S et al. Merqury: reference-free quality and phasing assessment for genome assemblies, BioRxiv. 2020; doi: https://doi.org/10.1101/2020.03.15.992941.

For the interest of the reviewer, QV prior to Illumina polishing was Q33.2 for the paternal assembly and Q32.7 for the maternal assembly. In VGP and other places, we are aiming for Q40, but in this case, we are lower than this likely due to the lower coverage we had per-haplotype (~25x per-haplotype).

Can you elaborate further on the types of artefact/contamination/manual curation that was flagged by gEval?

We have added the suggested details on page 7 by changing:

The assemblies were checked for contamination and further manually assessed and corrected using gEVAL [25].

to:

Assembly contaminants were identified and removed by checking the assemblies against vector/adapter sequences in the UniVec database [26], common contaminants in eukaryotes [27] and organelle sequences [28, 29]. The assemblies were also checked against other organism sequences from the RefSeq database version 94 [30]. This identified mouse contamination in two scaffolds which were subsequently removed. The assemblies were further manually assessed and corrected using gEVAL [31] with the available PacBio and 10X data. This process involved locating regions of zero or extreme PacBio read coverage and missed or mis-joins indicated by the 10X data, then evaluating the flagged discordances and correcting them where possible, which were typically missed joins, mis-joins and false duplications.

with added references:

26. UniVec Database. NCBI. https://www.ncbi.nlm.nih.gov/tools/vecscreen/univec. Accessed March 2019.

27. Contam_in_euks.fa.gz. ftp://ftp.ncbi.nlm.nih.gov/pub/kitts/contam_in_euks.fa.gz. Accessed March 2019.

28. Mito.nt.gz. ftp://ftp.ncbi.nlm.nih.gov/blast/db/ FASTA/mito.nt.gz. Accessed March 2019.

29. RefSeq Plastid Database. NCBI. ftp://ftp.ncbi.nlm.nih.gov/refseq/release/plastid. Accessed March 2019.

30. RefSeq: NCBI Reference Sequence Database. www.ncbi.nlm.nih.gov/refseq. Accessed March 2019.

I am interested to know more about how you constructed the plots in supplementary figure 1 (e.g. is this from custom parsing of reads lengths/counts in R or a direct visualisation of output from the assembler?). I ask with the vague hope that such qc descriptions might eventually become standardised so that direct comparisons of such metrics between assemblies might become straightforward.

For the interest of the reviewer, the plots in Supplementary Figure 1 are based on a Dazzler database (https://github.com/thegenemyers/DAZZ_DB) of the raw data. The command DBstats provided by DAZZ_DB outputs the histogram data used for these plots. We have a simple R script which parses this histogram data to make these plots from the database. This usually needs to be tweaked for individual datasets and for making a plot appropriate for a paper. We have added a sentence to the legend of Supplementary Figure 1 to briefly explain how the plot was constructed on page 2 of the Supplementary Material:

Plots were constructed from a Dazzler database [Supplementary Reference 1] of the raw data, using histogram data outputted by the 'DBstats' command.

with added supplementary reference:

1. The Dazzler Database Library. https://github.com/thegenemyers/DAZZ_DB. Accessed March 2019.

The treatment of the population samples (extraction and sequencing) is the same as for the parental short read sequencing. You could refer back to the earlier description here to avoid repetition.

We have implemented this suggestion on page 11 by replacing the repeated description with:

Whole genomic DNA extraction and short read sequencing was performed following the same method as described for short read sequencing of parental genomes during trio binning assembly.

For clarity, perhaps elaborate briefly on the samples/tissue types within the published RNAseq dataset you use for annotation

We have added this content on page 9 by changing:

> *Raw RNA-seq reads were obtained from Galarza et al. 2017 [47] under study accession number PRJEB14172*

to:

> *Raw RNA-seq reads were obtained from Galarza et al. 2017 [55] under study accession number PRJEB14172, which came from whole body tissue of A. plantaginis larvae from two families reared under two heat treatments.*

Discussion

Prompted by your statement "Successful haplotype separation was possible due to the high estimated heterozygosity…", it might be interesting to explore further how relevant the degree of heterozygosity really is to the success of this approach. Your statement is certainly right for highly fragmented assemblies but with long contigs, it is my sense that even a substantially lower degree of heterozygosity can still give strong support to contig origin and thus fully resolve the haplotypes.

We thank the reviewer for drawing attention to this statement. We have changed the statement on page 13:

> *Successful haplotype separation was possible due to the high estimated heterozygosity…*

to:

> *Successful haplotype separation was facilitated by the high estimated heterozygosity…*

with a corresponding change to a similar statement on page 4:

> *This was possible due to the high heterozygosity of the A. plantaginis genome…*

to:

> *This was facilitated by the high heterozygosity of the A. plantaginis genome…*

We recognise that trio binning can be successfully applied to organisms with lower heterozygosity. Indeed, the other species with published trio binned assemblies that we reference in our manuscript all have lower heterozygosities, ranging down to 0.1% (humans) in the original trio binning method paper Koren et al. 2018 (our reference [6]). We do not believe it is appropriate to our manuscript to further investigate how changing heterozygosity affects the success of the trio binning method, since our manuscript is about the application of trio binning for the assembly of a single species, and not about the method itself. Furthermore, this has already been addressed in the original Koren et al. 2018 paper (our reference [6]), which considers crosses with a range of heterozygosities, with an *Arabidopsis thaliana* cross (1.4%), *Homo sapiens* cross (0.1%) and *Bos taurus* x *Bos indicus* cross (0.9%), and discusses how higher heterozygosity enables the trio binning method work better.

We have included a sentence referring to this discussion about heterozygosity in Koren et al. 2018 (our reference [6]) in the revised manuscript. We also note that we only discuss the yak-cow hybrid heterozygosity value of 1.2% as a comparison, when in fact within species heterozygosity for previously published trio binned assemblies for zebra finch (1.6%) and *Arabidopsis* (1.4%) are both higher. We have therefore included a comparison to species heterozygosity from all previously published trio binned assemblies to improve our discussion breadth. These changes are located on page 13:

> *Successful haplotype separation was possible due to the high estimated heterozygosity (~1.9%) of the F1 offspring genome (Supplementary Figure 3), with greater levels of heterozygosity achieved through our same-species A. plantaginis cross than previously achieved through an inter-species cross between yak (Bos grunniens) and cattle (Bos taurus), which gave an F1 heterozygosity of ~1.2% [7].*

to:

> *Successful haplotype separation was facilitated by the high estimated heterozygosity (~1.9%) of the F1 offspring genome (Supplementary Figure 3), as it has previously been discussed that higher heterozygosity makes trio binning easier [6]. Indeed, greater heterozygosity levels were obtained through our same-species A. plantaginis cross than obtained previously through same-species crosses for zebra finch (~1.6%) [9], Arabidopsis (~1.4%) [6], bovid (~0.9%) [6] and human (~0.1%) [6] trio binned assemblies, as well as an inter-species yak (Bos grunniens) x cattle (Bos taurus) cross (~1.2%) [7].*

with a corresponding change on page 4:

> *... heterozygosity of the F1 offspring was estimated to be ~1.9%, exceeding levels (~1.2%) obtained when crossing different bovid species [7].*

to:

> *... heterozygosity of the F1 offspring was estimated to be ~1.9%, exceeding levels obtained in all other published trio binned assemblies through same-species crosses [6, 9, 10] and a yak-cow hybrid cross [7].*

Please consider including some mention of how obtaining appropriate trio samples may be a challenge in non-traditional model systems.

We thank the reviewer for this suggestion, and we have included this content to page 19 by adding the sentence:

Our assembly further highlights that trio binning can work well for a non-model system, provided a family trio can be obtained, which remains challenging for many non-model systems where it is difficult to obtain both parents and rear their offspring.

It is probably beyond the scope of this manuscript to touch on possible extensions of this approach to polyploid situations, but potentially this could be raised in the discussion.

We agree with the reviewer that this is beyond the scope of our manuscript. This is because we are not presenting our work as a novel method, but as an application of a previously published method to a new species, and note that reference [6] already briefly discusses the potential for applying similar ideas to polyploids.

Rather than "top tier" perhaps consider using "platinum quality", which seems to be gaining increasing use as a descriptor for assemblies with full chromosome scaffolds and haplotypes resolved across the entire genome.

We thank the reviewer for drawing attention to this statement. We have altered our statement on page 15:

> *Future chromosomal-level scaffolding work through Hi-C scaffolding technology [67] will elevate the A. plantaginis assembly quality to the top tier.*

to:

> *Future scaffolding work has the potential to lead to a chromosomal-scale A. plantaginis assembly.*

We believe the revised statement is more informative because it is often unclear what descriptors like “top tier” and “platinum quality” mean, as they are continually being redefined and debated. We have also removed the statement and reference about Hi-C scaffolding technology, since Hi-C is not the only way to achieve chromosomal-scale assemblies, so our original discussion statement is too narrow and potentially confusing.

Reviewer #2: The manuscript presents a haplotype-resolved genome assembly for the wood tiger moth that possesses a high-level whole genome heterozygosity level. The manuscript was well written and all the materials and methods were presented in a clear and organized manner. Importantly, it will contribute the studies on entomological genomics and meets the scope of GigaScience. I recommend it be accepted for publication after addressing several minor issues as follows:

1. the authors may want to explain more on the results from the KAT (Kmer based) analysis. For example, how did you obtain the initial Kmer set, from your assemblies or the shotgun reads? If you distinguished single-copy and multiple copy Kmers by tallying their occurrence number in the parental and maternal genomes, how did you define those 0-copy Kmer?
In addition, what is the proportion of your Kmer set that was utilized in the KAT analysis comparing to the entire Kmer set which can be obtained from the genome assembly or the shotgun reads. Will enlarge the K value help to increase the proportion and in turn, increase the power of the analysis?

We thank the reviewer for this feedback. To answer the reviewer’s questions, KAT plots a histogram of the frequencies of all of the Kmers in the raw read data set, coloured by the number of times that the Kmer appears in the assembly. 0-copy Kmers (shown in black in

Figure 2) are those found in the raw reads but not in the assembly. Changing the value of K does not change the proportion of Kmers used because we are using all Kmers for any value of K. Enlarging the value of K will increase the fraction of Kmers in the error (0-copy) and haploid (1-copy) peaks at the expense of the diploid (2-copy) peak, since a single discrepancy in a run of diploid sequence will affect K Kmers. We used a standard value of K=21 which clearly identifies error, haploid and diploid peaks for this species and data set. We have clarified these points in our manuscript further by changing the sentence in the legend of Figure 2 on page 28:

> *The first peak corresponds to k-mers missing from the assembly due to sequencing errors…*

to:

> *The first peak corresponds to k-mers present in the raw reads but missing from the assembly due to sequencing errors…*

and added a sentence describing the chosen cut-off K value on page 8:

> *For this analysis we used parameter K=21, which clearly identified error, haploid and diploid peaks for our dataset.*

2. the authors claim a whole genome heterozygosity level of 1.9% for the wood tiger moth, which, however, is estimated using a Kmer based method before obtaining the genome assembly. As you have already obtained the high-quality genome assembly, you may want to re-calculate it, and also it will be great to show readers that how the heterozygous sites distribute on the genome and briefly categorize them according to their types, e.g. SNPs, small InDels and large structure variances(SVs). Validating and visualizing those heterozygous sites makes the quality assessment part more complete.

We thank the reviewer for this suggestion, and we have included a heterozygosity analysis using the genome assembly in our revised manuscript. We estimated heterozygosity for a wild Finnish population (n=20), using resequenced genomes available from our population genomics analysis. We chose to estimate heterozygosity for this population as the parents used for trio binning assembly were from selection lines derived from a natural Finnish population, making this comparison highly relevant. This comparison is further useful to show that our reference genome is still representative of natural variation in the wild, which is important for population genomic studies.

To perform this analysis, we selected BAM files for the 20 Finnish individuals and called variants with monomorphic sites for the 5 largest scaffolds in the iArcPla.TrioW reference assembly. This subsample is representative of the whole genome as it covers 96.5 Mbp (15%) of the total assembly. The raw callset was filtered in the same way as performed in our population genomics analysis, then the number of SNPs and indels was calculated for each individual using VCFtools with a minor allele count filter of 1, to filter out sites which were different to the reference assembly in all individuals. We then computed individual

heterozygosity by dividing the total number of SNPs and indels by the total number of sites (minus the number of missing sites) per individual. This gave a mean heterozygosity value of ~1.8% across all individuals. This value is highly similar to our estimated heterozygosity for the F1 offspring genome (~1.9%), strengthening our result from kmer analysis. The slightly lower value in the wild might be explained by the parents used in our family trio being derived from different selection lines (3 generations), leading to greater heterozygosity between the trio binned parental haplotypes.

We have added Supplementary Text 2 (page 7 of Supplement) to describe the method for our heterozygosity analysis, and we have added Supplementary Table 4 (page 11 of Supplement), to report the number of SNPs, indels, total sites, and heterozygosity estimate per individual. On page 13 of our revised manuscript, we have changed:

> *Using GenomeScope, we estimated the F1 offspring haploid genome size to be 590Mb with a repeat fraction of 27% (Supplementary Figure 3).*

to:

> *Using GenomeScope [35], we estimated the F1 offspring haploid genome size to be 590Mb with a repeat fraction of 27% and whole genome heterozygosity of ~1.9% (Supplementary Figure 3). This value was similar to our mean heterozygosity estimate of ~1.8% in a wild, Finnish population (Supplementary Table 4; method described in Supplementary Text 1), demonstrating our reference assembly is representative of natural variation in a wild population. The slight discrepancy may be explained by the parents used for trio binning assembly being derived from different selection lines, leading to greater heterozygosity between the trio binned parental haplotypes.*

In response to the reviewer's suggestion, we have included an analysis of SVs present between the trio binned parental haplotypes. To do this, we performed a whole genome alignment between the parental haplotype assemblies and used Assemblytics to detect SVs, which is the same method used in the original trio binning paper Koren et al. 2016 (our reference [6]). Assemblytics reports the number and total bp affected by insertions, deletions, tandem expansions, tandem contractions, repeat expansions and repeat contractions, for size ranges of 50-500 bp and 500-10000 bp.

We have added a sentence describing our method on page 8:

> *Assemblytics [36] was used to detect structural variants (SVs) between the parental haplotypes. For this, a whole-genome alignment was performed between the haplotype assemblies using the Nucmer module of MUMmer version 3.23 [37] with Assemblytics recommended options.*

with a corresponding description of our results to page 13:

Assemblytics [36] detected 32203 SVs between the haplotype assemblies, affecting 51.6 Mbp of the genome (Supplementary Table 5; Supplementary Figure 4).

and added references:

36. Nattestad M, Schatz MC. Assemblytics: a web analytics tool for the detection of variants from an assembly. Bioinformatics. 2016; 32: 3021-3023.

37. Kurtz S, Phillippy A, Delcher AL et al. Versatile and open software for comparing large genomes. Genome Biology. 2004; 5: R12.

We have added Supplementary Figure 4 (page 5 of Supplement) and Supplementary Table 5 (page 11 of Supplement) to report and visualise the distribution of SV sizes present between the alignment of the parental haplotype assemblies.

Whilst we agree it would be interesting to characterise large SVs further, we believe that this type of extensive analysis is beyond the scope of our manuscript, which is a short Data Note to demonstrate the application of the trio binning method to another new species. We do not believe it is appropriate in our manuscript to visualise how heterozygous sites distribute across the genome, as we do not yet have an ordered, chromosomal-scale assembly, so this information would not be as useful at this moment in time. We further think that visualising heterozygosity along the genome would only be valuable if combined with a thorough investigation of the driving factors of the heterozygosity variation (such as selection, recombination, gene content etc.), which we also feel is beyond the scope of this Data Note paper. Without adding the suggested analysis, we maintain that we have provided a robust quality assessment of our trio binned reference assembly through KAT visualisation, the newly added QV analysis and the comparative assessment of contiguity metrics and BUSCO gene completeness against an unbinned assembly and 7 publicly available lepidopteran genomes, which place our assembly within the context of Lepidoptera genomics and clearly demonstrates it to be one of the best assemblies currently available for Lepidoptera.

3. the authors may want to give the unbinned data based assembly a more integrity process, so that makes a fair comparison. For example, you did not apply the 10X data to further scaffold the assembly, or maybe you have but I missed it. You'd better clarify it somewhere in your manuscript.

We thank the reviewer for this suggestion, and agree it would facilitate a fairer comparison than the one we report between scaffolded trio binned assemblies and an unscaffolded unbinned assembly. We have implemented the suggestion whilst avoiding the intensive process of producing a new assembly, by comparing unscaffolded versions of the trio binned assemblies against the unbinned assembly, which were all assembled using wtdbg2 followed by one round of Arrow polishing. We therefore compare binned and unbinned assemblies which are both unscaffolded, achieving a fair comparison in an equivalent manner to if we compare binned and unbinned assemblies which are both scaffolded, as suggested by the

reviewer. Furthermore, the newly included summary statistics for the unscaffolded trio binned assemblies can also be compared against the scaffolded trio binned assemblies, adding information on the quality improvement after scaffolding with 10X data.

In our revised manuscript, we have altered the methods on page 8:

> *Quality comparisons were conducted against an assembly of unbinned data from the same F1 offspring (iArcPla.wtdbg2), and against a representative selection of published lepidopteran reference genomes. For this, the latest versions of seven Lepidoptera species were downloaded…*

to:

> *A quality comparison was conducted by comparing unscaffolded, Arrow polished versions of the trio binned assemblies against an unscaffolded, Arrow polished assembly of unbinned data from the same F1 offspring (iArcPla.wtdbg2). Quality comparisons were also performed for the final, scaffolded trio binned assemblies against a representative selection of published lepidopteran reference genomes, for which the latest versions of seven Lepidoptera species were downloaded…*

and changed the results on page 14-15:

> *The paternal (iArcPla.TrioW) assembly contains 1069 scaffolds and N50=6.73 Mb, and the maternal (iArcPla.TrioY) assembly contains 1050 scaffolds and N50=9.77 Mb (Table 2). Both trio binned assemblies are more contiguous than the composite haploid iArcPla.wtdbg2 assembly produced using unbinned data from the same individual, which contains 2948 scaffolds and N50=1.84 Mb (Table 2; Figure 3A), illustrating the contiguity improvement we achieved by separating haplotypes before assembly. The trio binned assemblies are more complete than the unbinned assembly (complete BUSCOs: iArcPla.TrioW=98.1%; iArcPla.TrioY=96.4%; iArcPla.wtdbg2=95.4%). The trio binned assemblies are also less inflated than the unbinned assembly (assembly size: iArcPla.TrioW=585 Mb; iArcPla.TrioY=578 Mb; iArcPla.wtdbg2=615 Mb) and duplicated BUSCOs halved (duplicated BUSCOs: iArcPla.TrioW=1.2%; iArcPla.TrioY=1.1%; iArcPla.wtdbg2=2.1%), suggesting a reduction in artefactual assembly duplication at heterozygous sites through read binning (Table 2; Figure 3A).*

to:

> *The paternal (iArcPla.TrioW) assembly contains 1069 scaffolds with N50=6.73 Mb and 98.1% complete BUSCOs, and the maternal (iArcPla.TrioY) assembly contains 1050 scaffolds with N50=9.77 Mb and 96.4% complete BUSCOs (Table 3). Prior to scaffolding work with 10X data, both unscaffolded trio binned assemblies are already more contiguous and complete than a composite, haploid iArcPla.wtdbg2 assembly produced using unbinned data from the same individual (Table 2; Figure 3A). This illustrates the quality improvement achieved by separating haplotypes before assembly, and further improvement of the trio binned assemblies after scaffolding with 10X linked-reads (Table 2). The trio binned assemblies are also less inflated*

than the unbinned assembly with halved duplicated BUSCOs (Table 2; Figure 3A), suggesting a reduction in artefactual assembly duplication at heterozygous sites through read binning.

We have added quality statistics for the unscaffolded trio binned assemblies to Table 2 (page 16) and Supplementary Table 3 (page 10 of Supplement). We have also revised Figure 3A to show the revised cumulative contig length plot, and altered its legend on page 29:

Comparison of the A. plantaginis trio binned assemblies iArcPla.TrioW (paternal haplotype) and iArcPla.TrioY (maternal haplotype) against the composite assembly using unbinned data from the same individual (iArcPla.wtdbg2).

to:

Comparison of the unscaffolded A. plantaginis trio binned assemblies iArcPla.TrioW (paternal haplotype) and iArcPla.TrioY (maternal haplotype) against the unscaffolded composite assembly using unbinned data from the same individual (iArcPla.wtdbg2).
