# Supplementary material for: A haplotype-resolved, de novo genome assembly for the wood tiger moth (Arctia plantaginis) through trio binning
Source: Gigascience. 2020 Aug 18;9(8):giaa088. doi: 10.1093/gigascience/giaa088 (PMC7433188; doi:10.1093/gigascience/giaa088)
Supplement: giaa088_Supplemental_File [file giaa088_supplemental_file.docx]

**Supplementary Material**

**A haplotype-resolved, *de novo* genome assembly for the wood tiger moth (*Arctia plantaginis*) through trio binning**

Eugenie C. Yen^1*^, Shane A. McCarthy^2,3^, Juan A. Galarza^4^, Tomas N. Generalovic^1^, Sarah Pelan^3^, Petr Nguyen^5,6^, Joana I. Meier^1,7^, Ian A. Warren^1^, Johanna Mappes^4^, Richard Durbin^2,3^ and Chris D. Jiggins^1,7^

^1^ Department of Zoology, University of Cambridge, Cambridge, CB2 3EJ, United Kingdom

^2^ Department of Genetics, University of Cambridge, Cambridge, CB2 3EH, United Kingdom

^3^ Wellcome Sanger Institute, Wellcome Trust Genome Campus, Hinxton, Cambridge, CB10 1SA, United Kingdom

^4^ Department of Biological and Environmental Science, University of Jyväskylä FI-40014, Jyväskylä, Finland

^5^ Biology Centre of the Czech Academy of Sciences, Institute of Entomology, 370 05 České Budějovice, Czech Republic

^6^ University of South Bohemia, Faculty of Science, 370 05 České Budějovice, Czech Republic

^7^ St John’s College, CB2 1TP, Cambridge, United Kingdom

**^*^**Corresponding Author: Eugenie C. Yen. Department of Zoology, Downing Street, University of Cambridge, Cambridge, CB2 3EJ, UK. Email: eugeniecyen@gmail.com. Phone: +447402737277.

**
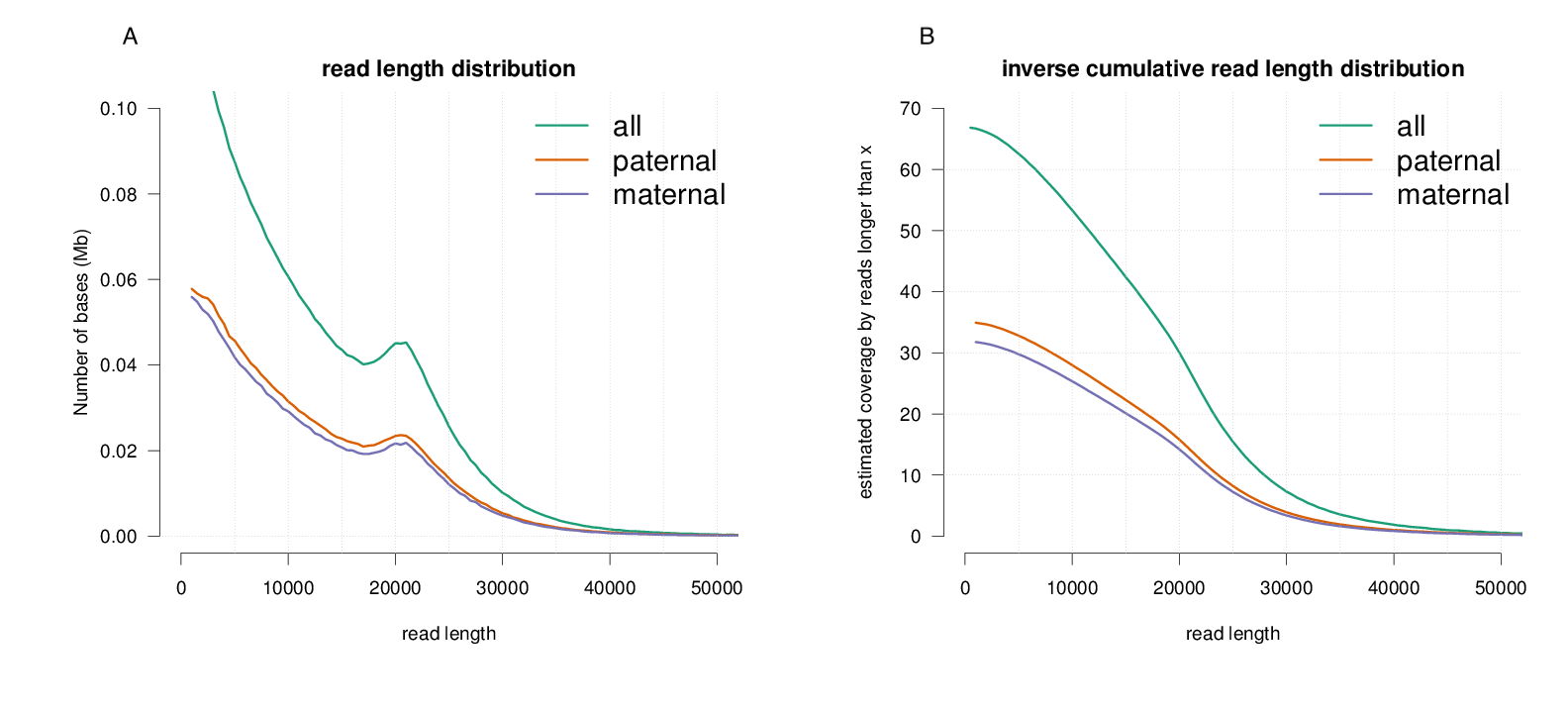
**

**Supplementary Figure 1**. **PacBio read length distribution for the *Arctia plantaginis* F1 offspring genome**. **(A)** Read length distribution of the entire PacBio dataset and for those reads assigned to either the maternal or paternal haplotypes. Plots were constructed from a Dazzler database [Supplementary Reference 1] of the raw data, using histogram data outputted by the ‘DBstats’ command. **(B)** Inverse cumulative read distribution to show coverage above a certain read length for the entire PacBio dataset and for those reads assigned to either the maternal or paternal haplotypes. A genome size of 590 Mb is assumed for the coverage estimate.

**
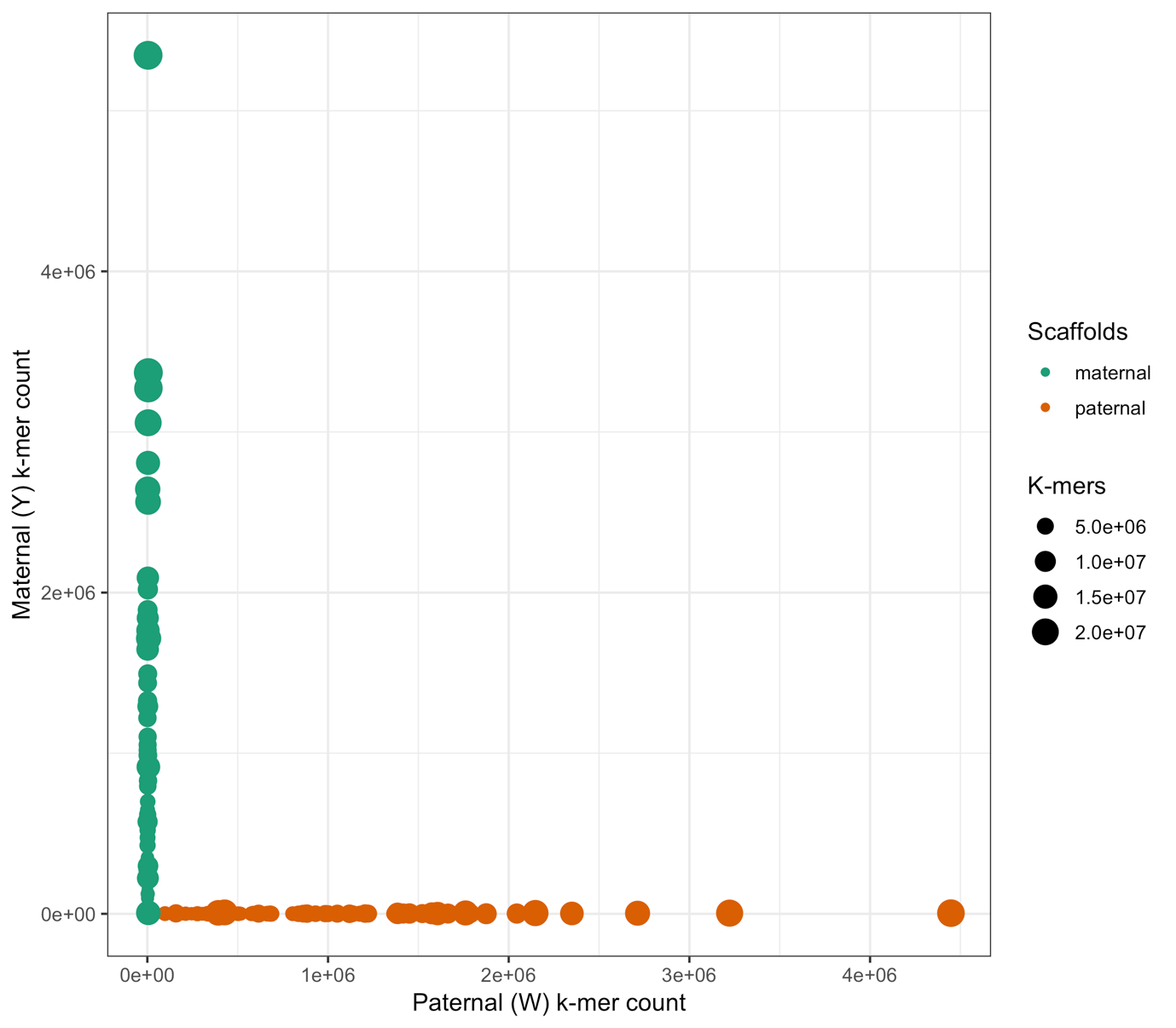
**

**Supplementary Figure 2.** **K-mer blob plot visualising haplotype specific k-mers for *Arctia plantaginis***. Plot showing for each scaffold the maternal (green) and paternal (red) assembly how many k-mers in that scaffold are found in the maternal or paternal k-mer sets. The size of the blob represents the total number of k-mers in that scaffold. There is good separation between the haplotypes, with each assembly consisting mostly of k-mers associated with the associated parental k-mer.

**
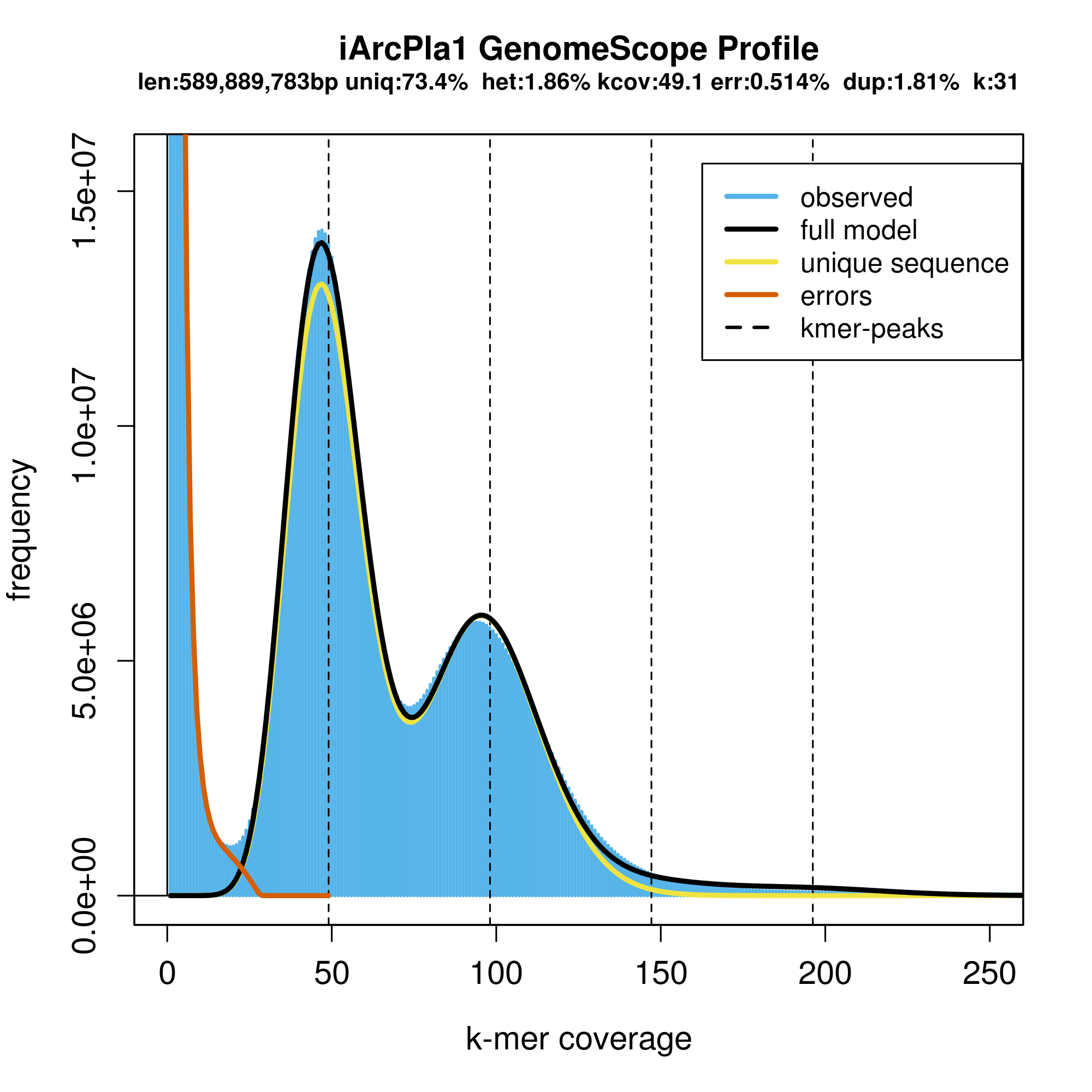
**

**Supplementary Figure 3.** **GenomeScope profile of the *Arctia plantaginis* F1 offspring genome**. Plot produced using GenomeScope [35]. K-mers are derived from 10X Genomics Illumina data. The estimated haploid genome size is 590Mb, heterozygosity 1.9% and repeat fraction 27%.


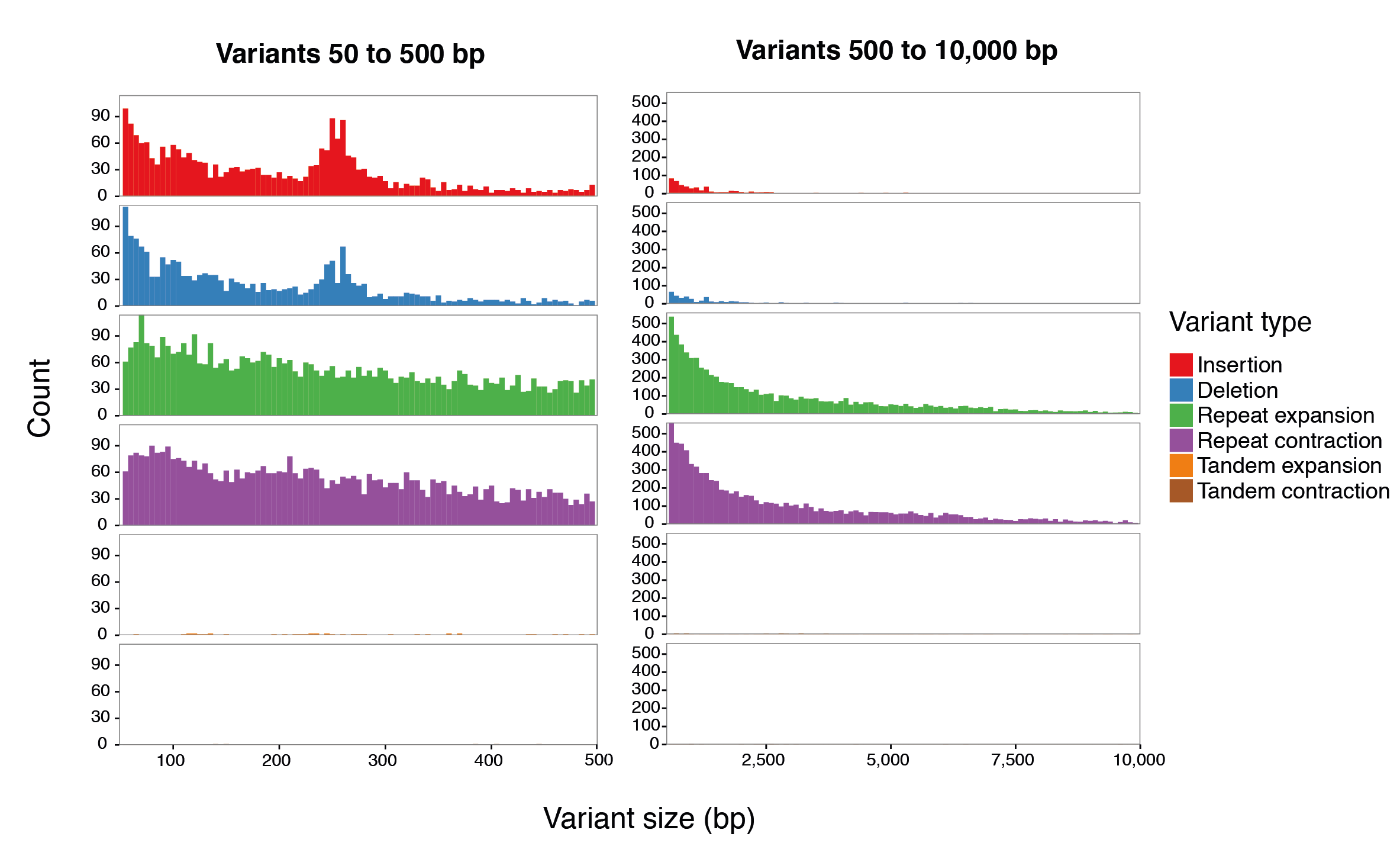


**Supplementary Figure 4. Comparison of structural variant sizes between the *Arctia plantaginis* trio binned haplotypes assemblies.** Plots produced using Assemblytics [36] comparing a whole genome alignment of the iArcPla.TrioW (paternal) and iArcPla.TrioY (maternal) assemblies, using the iArcPla.TrioW assembly as the reference. A total of 32203 structural variants were detected, affecting 51.6 Mbp of the genome.


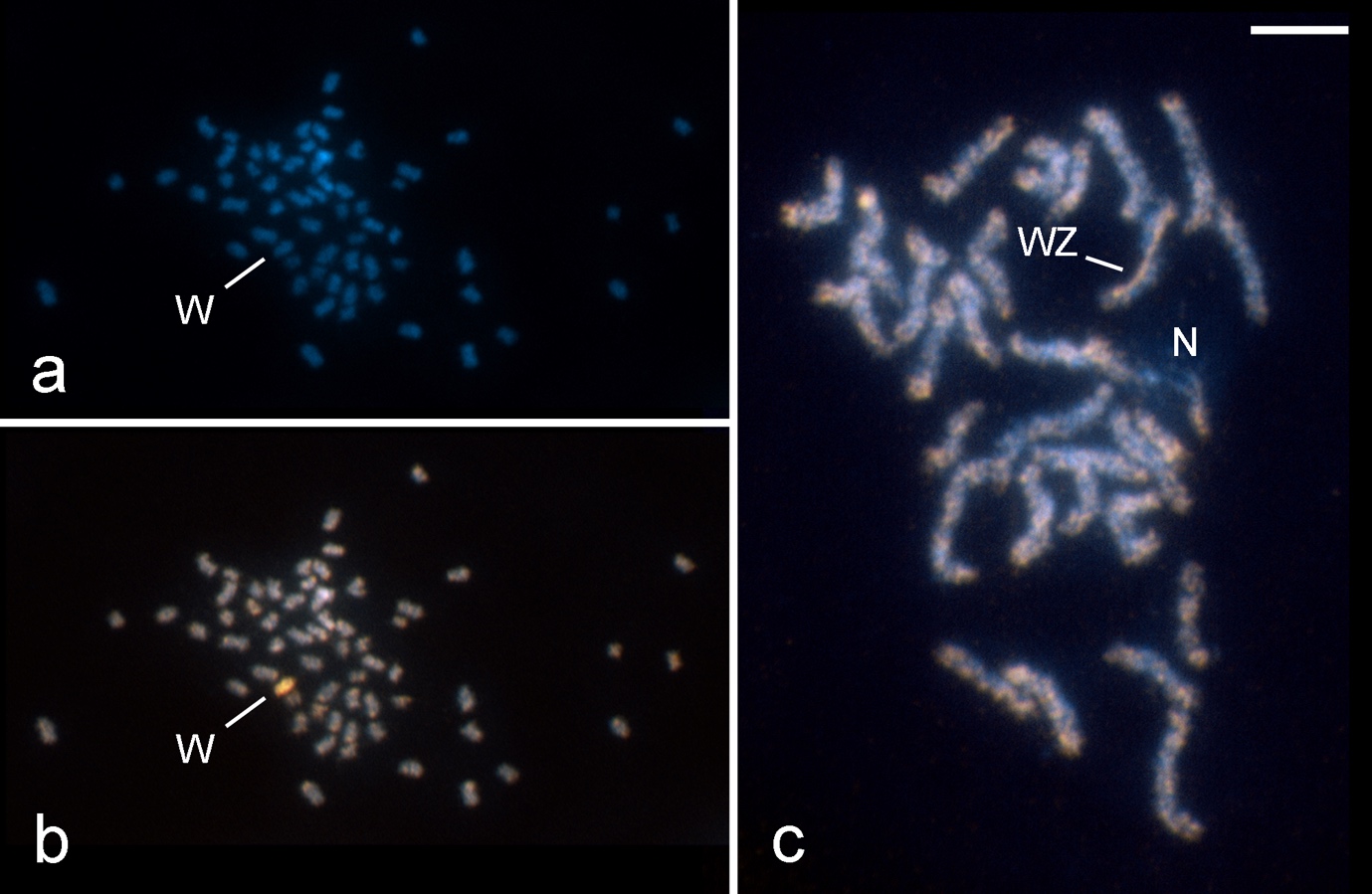


**Supplementary Figure 5.** **Cytogenetic analysis of *Arctia plantaginis* sex chromosomes.** Chromosomes were counterstained with DAPI (blue) and female derived genomic probe was labelled by Cy3 (orange). **(A)** Female mitotic metaphase consists of 2n=62 chromosomes. Note that the W sex chromosome, identified in **(B)**, is not highlighted by DAPI staining, indicating it is not formed by AT-rich heterochromatin. The only DAPI positive block corresponds to overlap of two chromosomes. **(B)** Genomic in situ hybridization (GISH) on the same mitotic nucleus, as in **(A)**, highlighted the W chromosome (orange). **(C)** Female pachytene nucleus consisting of n=31 bivalents were probed by GISH. The WZ sex chromosome bivalent was identified by different signal intensities of the W and Z chromosome threads. The probe (orange) painted almost the entire length of the W chromosome as well as various autosomal regions, but not the Z chromosome. This is probably due to hemizygosity of the Z chromosome in female gDNA used to construct the probe. Also note an interstitially localized nucleolus (N). Scale bar=5 µm.

**Supplementary Text 1. Results for cytogenetic analysis of *Arctia plantaginis* sex chromosomes.** In the female mitotic complement, DAPI staining did not highlight any chromosome which could represent the repeat-rich and thus AT-rich W chromosome (**Supplementary Figure 5A**; cf. Carabajal Paladino et al. 2019 [Supplementary Reference 2]). Therefore, genomic in situ hybridization (GISH) was employed to identify the W sex chromosome in both mitotic and meiotic female nuclei (**Supplementary Figure 5B, C**). In GISH, fluorescently labelled probe derived from female gDNA is hybridized to female chromosome preparations in surplus of unlabelled male competitor gDNA. The male competitor re-associates with autosomal and Z-linked probe sequences, i.e. sequences shared by both sexes. Female specific or enriched probe sequences then preferentially hybridize to chromosomes and highlight the female specific W chromosome in both mitotic and pachytene nuclei. In *A. plantaginis*, GISH clearly identified an inconspicuous medium size chromosome as the W chromosome in female mitotic complements (**Supplementary** **Figure 5A, B**). The WZ sex chromosome bivalent was not identified in DAPI stained female pachytene nuclei either (not shown). This contrasts with the garden tiger moth (*Arctia caja*), in which the W chromosome was easily recognized according to an AT-rich heterochromatin block deeply stained by DAPI [Supplementary Reference 3]. However, GISH with the female genomic probe labelled the W thread in the pachytene WZ sex chromosome bivalent. The hybridization signal covered almost entire W chromosome except for subtelomeric regions but also most autosomal regions (**Supplementary** **Figure 5C**).

**Supplementary Text 2. Method for estimating wild *Arctia plantaginis* genome heterozygosity.** We obtained an estimate for *A. plantaginis* genome heterozygosity in the wild, by computing heterozygosity for wild individuals sampled from the natural Finnish population (n=20; Central Finland: n=10, Southern Finland: n=10) in our population genomic analysis (**Figure 5; Supplementary Table 1**). This population was chosen as the parents used for trio binning assembly were from selection lines derived from natural Finnish populations. BAM files for these individuals were selected and variants were called with monomorphic sites for the 5 largest scaffolds in the iArcPla.TrioW reference assembly, covering 96.5 Mbp (15%) of the assembly. Variants were called for each sample using GATK HaplotypeCaller version 3.7 [68, 69] with the ‘-ERC BP_RESOLUTION’ option, followed by joint genotyping using GATK version 4.1 GenotypeGVCFs [68, 69] with ‘--heterozygosity 0.01’ and ‘--include-non-variant-sites’ options. The raw callset was quality filtered by applying the same thresholds as described in the methods for our population genomic analysis. Filters by depth (DP) of greater than half the mean (DP>137X) and less than double the mean (DP<550X) were also applied. The number of SNPs and indels present per individual was computed using VCFtools version 0.1.15 [71] with the ‘--mac 1’ option, and the number of missing sites was computed using VCFtools with the ‘--missing-indv’ option. Heterozygosity per individual was calculated by dividing the total number of SNPs and indels by the total number of sites present. The wild heterozygosity estimate we report is the mean of all individuals.

| **Sample ID** | **Morph**  **colour** | **Locality** | **Population** | **Latitude** | **Longitude** | **Altitude** |
| --- | --- | --- | --- | --- | --- | --- |
| CAM015089 | Yellow | Sandbäcken, Hanko | Southern Finland | 59.84741 | 23.132814 | 10 |
| CAM015094 | Yellow | Svanvik, Hanko | Southern Finland | 59.8393 | 23.1827 | 15 |
| CAM015133 | Yellow | Huosiaisnotko, Laukaa | Central Finland | 62.385908 | 25.818186 | 142 |
| CAM015134 | Yellow | Haralanharju, Kangasala | Central Finland | 61.534638 | 24.081058 | 99 |
| CAM015135 | Yellow | Heposuo, Laukaa | Central Finland | 62.347827 | 25.827675 | 141 |
| CAM015137 | White | Mäyrämäki, Jyväskylä | Central Finland | 62.228377 | 25.646546 | 194 |
| CAM015138 | White | Heposuo, Laukaa | Central Finland | 62.347827 | 25.827675 | 141 |
| CAM015139 | White | Lautaperä, Keuruu | Central Finland | 62.18704 | 24.874457 | 186 |
| CAM015140 | White | Heposuo, Laukaa | Central Finland | 62.347827 | 25.827675 | 141 |
| CAM015141 | White | Huosiaisnotko, Laukaa | Central Finland | 62.385908 | 25.818186 | 142 |
| CAM015142 | Yellow | Voiaskintie, Ulrikasund | Southern Finland | 60.433844 | 25.323725 | 65 |
| CAM015143 | Yellow | Tvärminne, Hanko | Southern Finland | 59.846 | 23.167 | 11 |
| CAM015144 | Yellow | Tvärminne, Hanko | Southern Finland | 59.846 | 23.167 | 11 |
| CAM015147 | White | Voiaskintie, Ulrikasund | Southern Finland | 60.433844 | 25.323725 | 65 |
| CAM015148 | White | Voiaskintie, Ulrikasund | Southern Finland | 60.433844 | 25.323725 | 65 |
| CAM015149 | White | Mosabackantie, Sipoo | Southern Finland | 60.333267 | 25.159055 | 30 |
| CAM015150 | White | Voiaskintie, Ulrikasund | Southern Finland | 60.433844 | 25.323725 | 65 |
| CAM015151 | White | Voiaskintie, Ulrikasund | Southern Finland | 60.433844 | 25.323725 | 65 |
| CAM015154 | White | Kanaküla 2 | Estonia | 58.270683 | 25.118833 | 44 |
| CAM015155 | White | Kanaküla 1.2 | Estonia | 58.263233 | 25.1449 | 45 |
| CAM015157 | White | Kanaküla 3 | Estonia | 58.301833 | 25.0969 | 36 |
| CAM015158 | White | Kanaküla 4 | Estonia | 58.2631 | 25.19075 | 56 |
| CAM015159 | White | Kanaküla 2 | Estonia | 58.270683 | 25.118833 | 44 |
| CAM015162 | Yellow | Thieves Hill, Aultmore, Keith | Scotland | 57.575232 | -3.048772 | 204 |
| CAM015163 | Yellow | Portknockie, Buckie | Scotland | 57.704217 | -2.876195 | 63 |
| CAM015170 | Yellow | Findlater Castle, Portsoy | Scotland | 57.69157 | -2.77265 | 47 |
| CAM015173 | Red | Borjomi-Kharagauli National Park | Georgia | 41.83177 | 42.83855 | 2189 |
| CAM015174 | Red | Borjomi-Kharagauli National Park | Georgia | 41.83177 | 42.83855 | 2189 |
| CAM015175 | Red | Borjomi-Kharagauli National Park | Georgia | 41.83177 | 42.83855 | 2189 |
| CAM015176 | Red | Borjomi-Kharagauli National Park | Georgia | 41.83177 | 42.83855 | 2189 |
| CAM015177 | Red | Borjomi-Kharagauli National Park | Georgia | 41.83177 | 42.83855 | 2189 |
| CAM015192 | Yellow | Heposuo, Laukaa | Central Finland | 62.347827 | 25.827675 | 141 |
| CAM015193 | Yellow | Heposuo, Laukaa | Central Finland | 62.347827 | 25.827675 | 141 |
| **CAM015202** | Yellow | Portknockie, Buckie | Scotland | 57.704217 | -2.876195 | 63 |
| **CAM015203** | Yellow | Portknockie, Buckie | Scotland | 57.704217 | -2.876195 | 63 |
| **CAM015204** | Yellow | Portknockie, Buckie | Scotland | 57.704217 | -2.876195 | 63 |
| **CAM015206** | Yellow | Portknockie, Buckie | Scotland | 57.704217 | -2.876195 | 63 |
| **CAM015207** | Yellow | Portknockie, Buckie | Scotland | 57.704217 | -2.876195 | 63 |
| **CAM015208** | Yellow | Portknockie, Buckie | Scotland | 57.704217 | -2.876195 | 63 |
| **CAM015211** | Yellow | Portknockie, Buckie | Scotland | 57.704217 | -2.876195 | 63 |

**Supplementary Table 1.** **Exact sampling localities of wild *Arctia plantaginis* males used in population genomic analysis**. Samples highlighted in bold are the F1 offspring of wild parents sampled from the localities presented in the table.

| **Sample ID** | **ENA Sample Accession Number** | **Mean sequencing**  **coverage** | **Number of raw reads** | **% of raw reads**  **mapped against**  **iArcPla.TrioW** |
| --- | --- | --- | --- | --- |
| CAM015089 | ERS4285276 | 13.3X | 67913274 | 99.02% |
| CAM015094 | ERS4285277 | 13.6X | 71591177 | 98.88% |
| CAM015133 | ERS4285280 | 16.0X | 91510105 | 98.94% |
| CAM015134 | ERS4285281 | 14.9X | 84005409 | 99.03% |
| CAM015135 | ERS4285282 | 14.4X | 82119542 | 99.00% |
| CAM015137 | ERS4285283 | 16.0X | 90270209 | 98.99% |
| CAM015138 | ERS4285284 | 14.7X | 83204225 | 98.98% |
| CAM015139 | ERS4285285 | 15.9X | 91852724 | 99.04% |
| CAM015140 | ERS4285286 | 15.1X | 85825617 | 98.96% |
| CAM015141 | ERS4285287 | 13.5X | 76558659 | 99.01% |
| CAM015142 | ERS4285288 | 15.0X | 84287184 | 99.04% |
| CAM015143 | ERS4285289 | 14.4X | 81283183 | 99.00% |
| CAM015144 | ERS4285290 | 15.3X | 88352550 | 98.97% |
| CAM015147 | ERS4285291 | 13.6X | 75042444 | 99.00% |
| CAM015148 | ERS4285292 | 16.0X | 89777636 | 99.00% |
| CAM015149 | ERS4285293 | 13.2X | 73604268 | 99.03% |
| CAM015150 | ERS4285294 | 13.0X | 72332606 | 98.98% |
| CAM015151 | ERS4285295 | 13.9X | 80159712 | 99.06% |
| CAM015154 | ERS4285296 | 12.9X | 71746939 | 98.93% |
| CAM015155 | ERS4285297 | 15.6X | 90247219 | 98.99% |
| CAM015157 | ERS4285298 | 14.4X | 82512306 | 98.99% |
| CAM015158 | ERS4285299 | 13.4X | 77179045 | 98.98% |
| CAM015159 | ERS4285300 | 13.9X | 79287964 | 98.99% |
| CAM015162 | ERS4285301 | 14.5X | 82886426 | 98.95% |
| CAM015163 | ERS4285302 | 14.7X | 83217243 | 98.94% |
| CAM015170 | ERS4285303 | 10.6X | 59511526 | 98.92% |
| CAM015173 | ERS4285304 | 11.1X | 68516047 | 98.09% |
| CAM015174 | ERS4285305 | 10.5X | 65751644 | 98.50% |
| CAM015175 | ERS4285306 | 12.1X | 75845780 | 98.45% |
| CAM015176 | ERS4285307 | 12.1X | 74743346 | 98.46% |
| CAM015177 | ERS4285308 | 13.2X | 83009022 | 98.44% |
| CAM015192 | ERS4285309 | 14.0X | 80351585 | 98.85% |
| CAM015193 | ERS4285310 | 10.6X | 58462162 | 99.04% |
| CAM015202 | ERS4285311 | 11.0X | 59903009 | 99.00% |
| CAM015203 | ERS4285312 | 10.1X | 57251863 | 99.03% |
| CAM015204 | ERS4285313 | 11.6X | 64780636 | 98.08% |
| CAM015206 | ERS4285314 | 15.0X | 87719259 | 98.90% |
| CAM015207 | ERS4285315 | 11.1X | 62197559 | 96.13% |
| CAM015208 | ERS4285316 | 11.9X | 64848486 | 98.96% |
| CAM015211 | ERS4285317 | 12.0X | 63799250 | 99.04% |

**Supplementary Table 2. Resequenced genome statistics for wild *Arctia plantaginis* males used in population genomic analysis.**

|  | **Complete**  **BUSCOs** | **Single copy**  **BUSCOs** | **Duplicated**  **BUSCOs** | **Fragmented**  **BUSCOs** | **Missing**  **BUSCOs** |
| --- | --- | --- | --- | --- | --- |
| ***Arctia plantaginis***  (binned: iArcPla.TrioW,  scaffolded assembly) | 98.1% | 96.9% | 1.2% | 0.5% | 1.4% |
| ***Arctia plantaginis***  (binned: iArcPla.TrioY,  scaffolded assembly) | 96.4% | 95.3% | 1.1% | 0.5% | 3.1% |
| ***Arctia plantaginis***  (binned: iArcPla.TrioW,  unscaffolded assembly) | 97.4% | 96.4% | 1.0% | 1.1% | 1.5% |
| ***Arctia plantaginis***  (binned: iArcPla.TrioY,  unscaffolded assembly) | 95.1% | 94.1% | 1.0% | 1.3% | 3.6% |
| ***Arctia plantaginis***  (unbinned: iArcPla.wtdbg2,  unscaffolded assembly) | 96.9% | 94.8% | 2.1% | 1.3% | 1.8% |
| ***Bicyclus anynana*** | 97.6% | 96.8% | 0.8% | 0.8% | 1.6% |
| ***Bombyx mori*** | 98.4% | 97.2% | 1.2% | 0.5% | 1.1% |
| ***Danaus plexippus*** | 98.0% | 96.0% | 2.0% | 1.0% | 1.0% |
| ***Heliconius melpomene*** | 97.7% | 96.7% | 1.0% | 1.0% | 1.3%% |
| ***Manduca sexta*** | 96.7% | 93.9% | 2.8% | 2.0% | 1.3% |
| ***Melitaea cinxia*** | 83.0% | 82.9% | 0.1% | 8.5% | 8.5% |
| ***Trichoplusia ni*** | 97.4% | 96.6% | 0.8% | 1.1% | 1.5% |

**Supplementary Table 3**. **Full BUSCO summary for *Arctia plantaginis* and seven publicly available lepidopteran genome assemblies**. BUSCO analysis performed using the ‘insecta_odb9’ gene set (n=1658) to evaluate assembly completeness.

| **Sample ID** | **Number of SNPs** | **Number of indels** | **Total number of sites** | **Heterozygosity** |
| --- | --- | --- | --- | --- |
| CAM015089 | 1025270 | 222512 | 70250203 | 0.018 |
| CAM015094 | 1024337 | 222179 | 70334075 | 0.018 |
| CAM015133 | 988466 | 220124 | 70338445 | 0.017 |
| CAM015134 | 1035845 | 229617 | 70380771 | 0.018 |
| CAM015135 | 1032098 | 226728 | 70360655 | 0.018 |
| CAM015137 | 1025090 | 225523 | 70390606 | 0.018 |
| CAM015138 | 1027335 | 226201 | 70358823 | 0.018 |
| CAM015139 | 1031080 | 226790 | 70400157 | 0.018 |
| CAM015140 | 1057119 | 232540 | 70436639 | 0.018 |
| CAM015141 | 1034642 | 227111 | 70338861 | 0.018 |
| CAM015142 | 1053956 | 232354 | 70383647 | 0.018 |
| CAM015143 | 1044757 | 229957 | 70375065 | 0.018 |
| CAM015144 | 1052177 | 231285 | 70396606 | 0.018 |
| CAM015147 | 1038589 | 119161 | 70339322 | 0.017 |
| CAM015148 | 1041668 | 230763 | 70439155 | 0.018 |
| CAM015149 | 1016197 | 222438 | 70230832 | 0.018 |
| CAM015150 | 1033955 | 225155 | 70383710 | 0.018 |
| CAM015151 | 1007757 | 218295 | 70353058 | 0.017 |
| CAM015192 | 953577 | 209485 | 70267185 | 0.017 |
| CAM015193 | 953522 | 205598 | 70209681 | 0.017 |

**Supplementary Table 4. Heterozygosity per male in the wild Finnish *Arctia plantaginis* population.** Heterozygosity was estimated for wild individuals sampled from Finland (n=20) by calling variants with monomorphic sites for the 5 largest scaffolds in the iArcPla.TrioW reference assembly. Heterozygosity was computed for each individual by dividing the total number of SNPs and indels by the total number of sites present.

|  | **50-500 bp** | | **500-10000 bp** | | **Total** | |
| --- | --- | --- | --- | --- | --- | --- |
|  | **Count** | **Total bp** | **Count** | **Total bp** | **Count** | **Total bp** |
| **Insertion** | 2445 | 473007 | 638 | 100713 | 3083 | 1482720 |
| **Deletion** | 2048 | 366934 | 516 | 873750 | 2564 | 1240684 |
| **Tandem expansion** | 40 | 10351 | 147 | 521754 | 187 | 532105 |
| **Tandem contraction** | 5 | 1528 | 19 | 53252 | 24 | 54780 |
| **Repeat expansion** | 4644 | 1099987 | 8069 | 21433470 | 12714 | 22543457 |
| **Repeat contraction** | 4660 | 1108213 | 8971 | 24676802 | 13631 | 25785015 |

**Supplementary Table 5. Structural variant sizes present between the *Arctia plantaginis* trio binned haplotypes assemblies.** Analysis was performed using Assemblytics [36], comparing a whole genome alignment of the iArcPla.TrioW (paternal) and iArcPla.TrioY (maternal) assemblies, with the iArcPla.TrioW assembly set as the reference.

**Supplementary References**

1. The Dazzler Database Library. https://github.com/thegenemyers/DAZZ_DB. Accessed March 2019.
2. Carabajal Paladino LZ, Provazníková I, Berger M, et al. Sex Chromosome Turnover in Moths of the Diverse Superfamily Gelechioidea. Genome Biol Evol. 2019; 11: 1307–1319.
3. Nguyen P, Sahara K, Yoshido A, Marec F. Evolutionary dynamics of rDNA clusters on chromosomes of moths and butterflies (Lepidoptera). Genetica. 2010; 138: 343–354.
