# Supplementary material for: A haplotype-resolved, de novo genome assembly for the wood tiger moth (Arctia plantaginis) through trio binning
Source: Gigascience. 2020 Aug 18;9(8):giaa088. doi: 10.1093/gigascience/giaa088 (PMC7433188; doi:10.1093/gigascience/giaa088)
Supplement: giaa088_Response_to_Reviewer_Comments_Original_Submission [file giaa088_response_to_reviewer_comments_original_submission.pdf]

Author's Response To Reviewer Comments

Close

Please see below for our point by point response to specific reviewer comments, and please see our attached Word document "Yen Response to Reviewers.docx" for a formatted version of the below text.

Reviewer 1 Specific Comments

"General
The resolution of the figures in the main submission, but not the supplement, is a little poor in the review copy."

We checked the resolution of our submitted figures, and we determine this issue should be specific to the reviewer copy only.

"Background
While I agree that full diploid reconstruction is/should be a eukaryotic genome assembly target and that there are few published examples, it might be worth also noting that the Vertebrate Genome Project contains, I believe, 12 trio-based assemblies that are publicly accessible."

We thank the reviewer for pointing this out, and we agree that the mentioned assemblies should be included. We have counted the number of trio-based assemblies currently available on the VGP GenomeArk data and changed the sentence on page 4 accordingly:

This represents the first trio binned assembly available for Insecta and indeed any invertebrate animal species, diversifying the organisms for which trio binning has been applied outside of bovids [6, 7], zebra finches [9], humans [6, 9, 10] and Arabidopsis thaliana [6].
to:
At the time of writing, this represents the first trio binned assembly available for an invertebrate animal species, diversifying the organisms for which published trio binned assemblies exist beyond bovids [6, 7], zebra finches [9], humans [6, 9, 10], Arabidopsis thaliana [6] and additional trio binned assemblies available for eight other vertebrate species on the Vertebrate Genomes Project GenomeArk database [11].

with added reference:

11. Vertebrate Genomes Project GenomeArk. https://vgp.github.io/genomeark. Accessed May 2020.

"Methods
Please confirm that you have not done any of the following (and if you have, please incorporate details in the methods)
Any additional quality trimming of RNAseq reads beyond adaptor removal with cutadapt?"

We have added the suggested details on page 9 by changing:

RNA-seq reads were trimmed for adapter contamination using cutadapt version 1.8.1 [48] and quality controlled pre and post trimming with fastqc version 0.11.8 [49].
to:
Using cutadapt version 1.8.1 [56], RNA-seq reads were trimmed for adapter contamination and quality trimmed at both ends of each read using a quality value of 3 (-q 3,3). Quality control was performed pre and post trimming with fastqc version 0.11.8 [57].

"Any pre-processing of PacBio reads to remove adaptor contamination etc?"

We confirm that there was no pre-processing of PacBio reads. We performed adapter contamination

removal during the assembly curation stage, for which details have been added in our response to the reviewer comment “Can you elaborate further on the types of artefact/contamination/manual curation that was flagged by gEval?”

"Please consider also calculating and reporting QV to provide an estimate of assembly accuracy (presenting figures before and after polishing with the 10x reads would be of interest)."

We thank the reviewer for this suggestion and agree that reporting QV is useful. We have included a QV analysis in our revised manuscript. We have added a sentence describing the method on page 8:

To provide an estimate of assembly consensus accuracy, a quality value (QV) was computed for each assembly using Merqury version 1.0 [34].

and added a sentence describing the results on page 12-13:

Using Merqury [34], we estimated QV scores of Q34.7 for the paternal (iArcPla.TrioW) assembly and Q34.2 for the maternal (iArcPla.TrioY) assembly, indicating high (>99.9%) assembly accuracy.

with added reference:

34. Rhie A, Walenz BP, Koren S et al. Merqury: reference-free quality and phasing assessment for genome assemblies, BioRxiv. 2020; doi: https://doi.org/10.1101/2020.03.15.992941.

For the interest of the reviewer, QV prior to Illumina polishing was Q33.2 for the paternal assembly and Q32.7 for the maternal assembly. In VGP and other places, we are aiming for Q40, but in this case, we are lower than this likely due to the lower coverage we had per-haplotype (~25x per-haplotype).

"Can you elaborate further on the types of artefact/contamination/manual curation that was flagged by gEval?"

We have added the suggested details on page 7 by changing:

The assemblies were checked for contamination and further manually assessed and corrected using gEVAL [25].
to:
Assembly contaminants were identified and removed by checking the assemblies against vector/adapter sequences in the UniVec database [26], common contaminants in eukaryotes [27] and organelle sequences [28, 29]. The assemblies were also checked against other organism sequences from the RefSeq database version 94 [30]. This identified mouse contamination in two scaffolds which were subsequently removed. The assemblies were further manually assessed and corrected using gEVAL [31] with the available PacBio and 10X data. This process involved locating regions of zero or extreme PacBio read coverage and missed or mis-joins indicated by the 10X data, then evaluating the flagged discordances and correcting them where possible, which were typically missed joins, mis-joins and false duplications.

with added references:

26. UniVec Database. NCBI. https://www.ncbi.nlm.nih.gov/tools/vecscreen/univec. Accessed March 2019.

27. Contam_in_euks.fa.gz. ftp://ftp.ncbi.nlm.nih.gov/pub/kitts/contam_in_euks.fa.gz. Accessed March 2019.

28. Mito.nt.gz. ftp://ftp.ncbi.nlm.nih.gov/blast/db/ FASTA/mito.nt.gz. Accessed March 2019.

29. RefSeq Plastid Database. NCBI. ftp://ftp.ncbi.nlm.nih.gov/refseq/release/plastid. Accessed March 2019.

30. RefSeq: NCBI Reference Sequence Database. www.ncbi.nlm.nih.gov/refseq. Accessed March 2019.

"I am interested to know more about how you constructed the plots in supplementary figure 1 (e.g. is this from custom parsing of reads lengths/counts in R or a direct visualisation of output from the

assembler?). I ask with the vague hope that such qc descriptions might eventually become standardised so that direct comparisons of such metrics between assemblies might become straightforward."

For the interest of the reviewer, the plots in Supplementary Figure 1 are based on a Dazzler database (https://github.com/thegenemyers/DAZZ_DB) of the raw data. The command DBstats provided by DAZZ_DB outputs the histogram data used for these plots. We have a simple R script which parses this histogram data to make these plots from the database. This usually needs to be tweaked for individual datasets and for making a plot appropriate for a paper. We have added a sentence to the legend of Supplementary Figure 1 to briefly explain how the plot was constructed on page 2 of the Supplementary Material:

Plots were constructed from a Dazzler database [Supplementary Reference 1] of the raw data, using histogram data outputted by the ‘DBstats’ command.

with added supplementary reference:

1. The Dazzler Database Library. https://github.com/thegenemyers/DAZZ_DB. Accessed March 2019.

"The treatment of the population samples (extraction and sequencing) is the same as for the parental short read sequencing. You could refer back to the earlier description here to avoid repetition."

We have implemented this suggestion on page 11 by replacing the repeated description with:

Whole genomic DNA extraction and short read sequencing was performed following the same method as described for short read sequencing of parental genomes during trio binning assembly.

For clarity, perhaps elaborate briefly on the samples/tissue types within the published RNAseq dataset you use for annotation

We have added this content on page 9 by changing:

Raw RNA-seq reads were obtained from Galarza et al. 2017 [47] under study accession number PRJEB14172
to:
Raw RNA-seq reads were obtained from Galarza et al. 2017 [55] under study accession number PRJEB14172, which came from whole body tissue of A. plantaginis larvae from two families reared under two heat treatments.

"Discussion
Prompted by your statement "Successful haplotype separation was possible due to the high estimated heterozygosity…", it might be interesting to explore further how relevant the degree of heterozygosity really is to the success of this approach. Your statement is certainly right for highly fragmented assemblies but with long contigs, it is my sense that even a substantially lower degree of heterozygosity can still give strong support to contig origin and thus fully resolve the haplotypes."

We thank the reviewer for drawing attention to this statement. We have changed the statement on page 13:

Successful haplotype separation was possible due to the high estimated heterozygosity…
to:
Successful haplotype separation was facilitated by the high estimated heterozygosity…

with a corresponding change to a similar statement on page 4:

This was possible due to the high heterozygosity of the A. plantaginis genome…
to:
This was facilitated by the high heterozygosity of the A. plantaginis genome…

We recognise that trio binning can be successfully applied to organisms with lower heterozygosity. Indeed, the other species with published trio binned assemblies that we reference in our manuscript all have lower heterozygosities, ranging down to 0.1% (humans) in the original trio binning method paper Koren et al. 2018 (our reference [6]). We do not believe it is appropriate to our manuscript to further

investigate how changing heterozygosity affects the success of the trio binning method, since our manuscript is about the application of trio binning for the assembly of a single species, and not about the method itself. Furthermore, this has already been addressed in the original Koren et al. 2018 paper (our reference [6]), which considers crosses with a range of heterozygosities, with an Arabidopsis thaliana cross (1.4%), Homo sapiens cross (0.1%) and Bos taurus x Bos indicus cross (0.9%), and discusses how higher heterozygosity enables the trio binning method work better.

We have included a sentence referring to this discussion about heterozygosity in Koren et al. 2018 (our reference [6]) in the revised manuscript. We also note that we only discuss the yak-cow hybrid heterozygosity value of 1.2% as a comparison, when in fact within species heterozygosity for previously published trio binned assemblies for zebra finch (1.6%) and Arabidopsis (1.4%) are both higher. We have therefore included a comparison to species heterozygosity from all previously published trio binned assemblies to improve our discussion breadth. These changes are located on page 13:

Successful haplotype separation was possible due to the high estimated heterozygosity (~1.9%) of the F1 offspring genome (Supplementary Figure 3), with greater levels of heterozygosity achieved through our same-species A. plantaginis cross than previously achieved through an inter-species cross between yak (Bos grunniens) and cattle (Bos taurus), which gave an F1 heterozygosity of ~1.2% [7].
to:
Successful haplotype separation was facilitated by the high estimated heterozygosity (~1.9%) of the F1 offspring genome (Supplementary Figure 3), as it has previously been discussed that higher heterozygosity makes trio binning easier [6]. Indeed, greater heterozygosity levels were obtained through our same-species A. plantaginis cross than obtained previously through same-species crosses for zebra finch (~1.6%) [9], Arabidopsis (~1.4%) [6], bovid (~0.9%) [6] and human (~0.1%) [6] trio binned assemblies, as well as an inter-species yak (Bos grunniens) x cattle (Bos taurus) cross (~1.2%) [7].

with a corresponding change on page 4:

… heterozygosity of the F1 offspring was estimated to be ~1.9%, exceeding levels (~1.2%) obtained when crossing different bovid species [7].
to:
… heterozygosity of the F1 offspring was estimated to be ~1.9%, exceeding levels obtained in all other published trio binned assemblies through same-species crosses [6, 9, 10] and a yak-cow hybrid cross [7].

"Please consider including some mention of how obtaining appropriate trio samples may be a challenge in non-traditional model systems."

We thank the reviewer for this suggestion, and we have included this content to page 19 by adding the sentence:

Our assembly further highlights that trio binning can work well for a non-model system, provided a family trio can be obtained, which remains challenging for many non-model systems where it is difficult to obtain both parents and rear their offspring.

"It is probably beyond the scope of this manuscript to touch on possible extensions of this approach to polyploid situations, but potentially this could be raised in the discussion."

We agree with the reviewer that this is beyond the scope of our manuscript. This is because we are not presenting our work as a novel method, but as an application of a previously published method to a new species, and note that reference [6] already briefly discusses the potential for applying similar ideas to polyploids.

"Rather than "top tier" perhaps consider using "platinum quality", which seems to be gaining increasing use as a descriptor for assemblies with full chromosome scaffolds and haplotypes resolved across the entire genome."

We thank the reviewer for drawing attention to this statement. We have altered our statement on page 15:

Future chromosomal-level scaffolding work through Hi-C scaffolding technology [67] will elevate the A.

plantaginis assembly quality to the top tier.
to:
Future scaffolding work has the potential to lead to a chromosomal-scale A. plantaginis assembly.

We believe the revised statement is more informative because it is often unclear what descriptors like “top tier” and “platinum quality” mean, as they are continually being redefined and debated. We have also removed the statement and reference about Hi-C scaffolding technology, since Hi-C is not the only way to achieve chromosomal-scale assemblies, so our original discussion statement is too narrow and potentially confusing.

Reviewer 2 Specific Comments

"1. the authors may want to explain more on the results from the KAT (Kmer based) analysis. For example, how did you obtain the initial Kmer set, from your assemblies or the shotgun reads? If you distinguished single-copy and multiple copy Kmers by tallying their occurrence number in the parental and maternal genomes, how did you define those 0-copy Kmer?
In addition, what is the proportion of your Kmer set that was utilized in the KAT analysis comparing to the entire Kmer set which can be obtained from the genome assembly or the shotgun reads. Will enlarge the K value help to increase the proportion and in turn, increase the power of the analysis?"

We thank the reviewer for this feedback. To answer the reviewer’s questions, KAT plots a histogram of the frequencies of all of the Kmers in the raw read data set, coloured by the number of times that the Kmer appears in the assembly. 0-copy Kmers (shown in black in Figure 2) are those found in the raw reads but not in the assembly. Changing the value of K does not change the proportion of Kmers used because we are using all Kmers for any value of K. Enlarging the value of K will increase the fraction of Kmers in the error (0-copy) and haploid (1-copy) peaks at the expense of the diploid (2-copy) peak, since a single discrepancy in a run of diploid sequence will affect K Kmers. We used a standard value of K=21 which clearly identifies error, haploid and diploid peaks for this species and data set. We have clarified these points in our manuscript further by changing the sentence in the legend of Figure 2 on page 28:

The first peak corresponds to k-mers missing from the assembly due to sequencing errors…
to:
The first peak corresponds to k-mers present in the raw reads but missing from the assembly due to sequencing errors…

and added a sentence describing the chosen cut-off K value on page 8:

For this analysis we used parameter K=21, which clearly identified error, haploid and diploid peaks for our dataset.

"2. the authors claim a whole genome heterozygosity level of 1.9% for the wood tiger moth, which, however, is estimated using a Kmer based method before obtaining the genome assembly. As you have already obtained the high-quality genome assembly, you may want to re-calculate it, and also it will be great to show readers that how the heterozygous sites distribute on the genome and briefly categorize them according to their types, e.g. SNPs, small InDels and large structure variances(SVs). Validating and visualizing those heterozygous sites makes the quality assessment part more complete."

We thank the reviewer for this suggestion, and we have included a heterozygosity analysis using the genome assembly in our revised manuscript. We estimated heterozygosity for a wild Finnish population (n=20), using resequenced genomes available from our population genomics analysis. We chose to estimate heterozygosity for this population as the parents used for trio binning assembly were from selection lines derived from a natural Finnish population, making this comparison highly relevant. This comparison is further useful to show that our reference genome is still representative of natural variation in the wild, which is important for population genomic studies.

To perform this analysis, we selected BAM files for the 20 Finnish individuals and called variants with monomorphic sites for the 5 largest scaffolds in the iArcPla.TrioW reference assembly. This subsample is representative of the whole genome as it covers 96.5 Mbp (15%) of the total assembly. The raw callset was filtered in the same way as performed in our population genomics analysis, then the number of SNPs and indels was calculated for each individual using VCFtools with a minor allele count filter of 1, to

filter out sites which were different to the reference assembly in all individuals. We then computed individual heterozygosity by dividing the total number of SNPs and indels by the total number of sites (minus the number of missing sites) per individual. This gave a mean heterozygosity value of ~1.8% across all individuals. This value is highly similar to our estimated heterozygosity for the F1 offspring genome (~1.9%), strengthening our result from kmer analysis. The slightly lower value in the wild might be explained by the parents used in our family trio being derived from different selection lines (3 generations), leading to greater heterozygosity between the trio binned parental haplotypes.

We have added Supplementary Text 2 (page 7 of Supplement) to describe the method for our heterozygosity analysis, and we have added Supplementary Table 4 (page 11 of Supplement), to report the number of SNPs, indels, total sites, and heterozygosity estimate per individual. On page 13 of our revised manuscript, we have changed:

Using GenomeScope, we estimated the F1 offspring haploid genome size to be 590Mb with a repeat fraction of 27% (Supplementary Figure 3).
to:
Using GenomeScope [35], we estimated the F1 offspring haploid genome size to be 590Mb with a repeat fraction of 27% and whole genome heterozygosity of ~1.9% (Supplementary Figure 3). This value was similar to our mean heterozygosity estimate of ~1.8% in a wild, Finnish population (Supplementary Table 4; method described in Supplementary Text 1), demonstrating our reference assembly is representative of natural variation in a wild population. The slight discrepancy may be explained by the parents used for trio binning assembly being derived from different selection lines, leading to greater heterozygosity between the trio binned parental haplotypes.

In response to the reviewer's suggestion, we have included an analysis of SVs present between the trio binned parental haplotypes. To do this, we performed a whole genome alignment between the parental haplotype assemblies and used Assemblytics to detect SVs, which is the same method used in the original trio binning paper Koren et al. 2016 (our reference [6]). Assemblytics reports the number and total bp affected by insertions, deletions, tandem expansions, tandem contractions, repeat expansions and repeat contractions, for size ranges of 50-500 bp and 500-10000 bp.

We have added a sentence describing our method on page 8:

Assemblytics [36] was used to detect structural variants (SVs) between the parental haplotypes. For this, a whole-genome alignment was performed between the haplotype assemblies using the Nucmer module of MUMmer version 3.23 [37] with Assemblytics recommended options.

with a corresponding description of our results to page 13:

Assemblytics [36] detected 32203 SVs between the haplotype assemblies, affecting 51.6 Mbp of the genome (Supplementary Table 5; Supplementary Figure 4).

and added references:

36. Nattestad M, Schatz MC. Assemblytics: a web analytics tool for the detection of variants from an assembly. Bioinformatics. 2016; 32: 3021-3023.

37. Kurtz S, Phillippy A, Delcher AL et al. Versatile and open software for comparing large genomes. Genome Biology. 2004; 5: R12.

We have added Supplementary Figure 4 (page 5 of Supplement) and Supplementary Table 5 (page 11 of Supplement) to report and visualise the distribution of SV sizes present between the alignment of the parental haplotype assemblies.

Whilst we agree it would be interesting to characterise large SVs further, we believe that this type of extensive analysis is beyond the scope of our manuscript, which is a short Data Note to demonstrate the application of the trio binning method to another new species. We do not believe it is appropriate in our manuscript to visualise how heterozygous sites distribute across the genome, as we do not yet have an ordered, chromosomal-scale assembly, so this information would not be as useful at this moment in time. We further think that visualising heterozygosity along the genome would only be valuable if combined with a thorough investigation of the driving factors of the heterozygosity variation (such as selection, recombination, gene content etc.), which we also feel is beyond the scope of this Data Note

paper. Without adding the suggested analysis, we maintain that we have provided a robust quality assessment of our trio binned reference assembly through KAT visualisation, the newly added QV analysis and the comparative assessment of contiguity metrics and BUSCO gene completeness against an unbinned assembly and 7 publicly available lepidopteran genomes, which place our assembly within the context of Lepidoptera genomics and clearly demonstrates it to be one of the best assemblies currently available for Lepidoptera.

"3. the authors may want to give the unbinned data based assembly a more integrity process, so that makes a fair comparison. For example, you did not apply the 10X data to further scaffold the assembly, or maybe you have but I missed it. You'd better clarify it somewhere in your manuscript."

We thank the reviewer for this suggestion, and agree it would facilitate a fairer comparison than the one we report between scaffolded trio binned assemblies and an unscaffolded unbinned assembly. We have implemented the suggestion whilst avoiding the intensive process of producing a new assembly, by comparing unscaffolded versions of the trio binned assemblies against the unbinned assembly, which were all assembled using wtdbg2 followed by one round of Arrow polishing. We therefore compare binned and unbinned assemblies which are both unscaffolded, achieving a fair comparison in an equivalent manner to if we compare binned and unbinned assemblies which are both scaffolded, as suggested by the reviewer. Furthermore, the newly included summary statistics for the unscaffolded trio binned assemblies can also be compared against the scaffolded trio binned assemblies, adding information on the quality improvement after scaffolding with 10X data.

In our revised manuscript, we have altered the methods on page 8:

Quality comparisons were conducted against an assembly of unbinned data from the same F1 offspring (iArcPla.wtdbg2), and against a representative selection of published lepidopteran reference genomes. For this, the latest versions of seven Lepidoptera species were downloaded…
to:
A quality comparison was conducted by comparing unscaffolded, Arrow polished versions of the trio binned assemblies against an unscaffolded, Arrow polished assembly of unbinned data from the same F1 offspring (iArcPla.wtdbg2). Quality comparisons were also performed for the final, scaffolded trio binned assemblies against a representative selection of published lepidopteran reference genomes, for which the latest versions of seven Lepidoptera species were downloaded…

and changed the results on page 14-15:

The paternal (iArcPla.TrioW) assembly contains 1069 scaffolds and N50=6.73 Mb, and the maternal (iArcPla.TrioY) assembly contains 1050 scaffolds and N50=9.77 Mb (Table 2). Both trio binned assemblies are more contiguous than the composite haploid iArcPla.wtdbg2 assembly produced using unbinned data from the same individual, which contains 2948 scaffolds and N50=1.84 Mb (Table 2; Figure 3A), illustrating the contiguity improvement we achieved by separating haplotypes before assembly. The trio binned assemblies are more complete than the unbinned assembly (complete BUSCOs: iArcPla.TrioW=98.1%; iArcPla.TrioY=96.4%; iArcPla.wtdbg2=95.4%). The trio binned assemblies are also less inflated than the unbinned assembly (assembly size: iArcPla.TrioW=585 Mb; iArcPla.TrioY=578 Mb; iArcPla.wtdbg2=615 Mb) and duplicated BUSCOs halved (duplicated BUSCOs: iArcPla.TrioW=1.2%; iArcPla.TrioY=1.1%; iArcPla.wtdbg2=2.1%), suggesting a reduction in artefactual assembly duplication at heterozygous sites through read binning (Table 2; Figure 3A).
to:
The paternal (iArcPla.TrioW) assembly contains 1069 scaffolds with N50=6.73 Mb and 98.1% complete BUSCOs, and the maternal (iArcPla.TrioY) assembly contains 1050 scaffolds with N50=9.77 Mb and 96.4% complete BUSCOs (Table 3). Prior to scaffolding work with 10X data, both unscaffolded trio binned assemblies are already more contiguous and complete than a composite, haploid iArcPla.wtdbg2 assembly produced using unbinned data from the same individual (Table 2; Figure 3A). This illustrates the quality improvement achieved by separating haplotypes before assembly, and further improvement of the trio binned assemblies after scaffolding with 10X linked-reads (Table 2). The trio binned assemblies are also less inflated than the unbinned assembly with halved duplicated BUSCOs (Table 2; Figure 3A), suggesting a reduction in artefactual assembly duplication at heterozygous sites through read binning.

We have added quality statistics for the unscaffolded trio binned assemblies to Table 2 (page 16) and Supplementary Table 3 (page 10 of Supplement). We have also revised Figure 3A to show the revised cumulative contig length plot, and altered its legend on page 29:

Comparison of the A. plantaginis trio binned assemblies iArcPla.TrioW (paternal haplotype) and iArcPla.TrioY (maternal haplotype) against the composite assembly using unbinned data from the same individual (iArcPla.wtdbg2).
to:
Comparison of the unscaffolded A. plantaginis trio binned assemblies iArcPla.TrioW (paternal haplotype) and iArcPla.TrioY (maternal haplotype) against the unscaffolded composite assembly using unbinned data from the same individual (iArcPla.wtdbg2).

Close
