# Supplementary material for: A haplotype-resolved, de novo genome assembly for the wood tiger moth (Arctia plantaginis) through trio binning
Source: Gigascience. 2020 Aug 18;9(8):giaa088. doi: 10.1093/gigascience/giaa088 (PMC7433188; doi:10.1093/gigascience/giaa088)
Supplement: giaa088_Reviewer_1_Report_Original_Submission — Annabel Charlotte Whibley -- 3/31/2020 Reviewed [file giaa088_reviewer_1_report_original_submission.pdf]

Reviewer Report

Title: A haplotype-resolved, de novo genome assembly for the wood tiger moth (Arctia plantaginis) through trio binning

Version: Original Submission **Date:** 3/31/2020

Reviewer name: Annabel Charlotte Whibley

Reviewer Comments to Author:

Yen and colleagues present a haplotype-resolved draft assembly of the wood tiger moth genome using a trio-binning approach to leverage heterozygosity, a component of genome biology that is typically considered a confounding factor in the quest for a high quality assembly.

The manuscript is clearly structured and well-written. The primary data and assembly strategy are well-described with comprehensive and appropriate inclusion of methods, software versions and parameters. I can confirm that the ENA accession number is active and populated with the appropriate raw data. The assembly itself appears to be of excellent quality (in terms of completeness and contiguity) and the comparisons to a composite haplotype assembly from the same primary reads as well as to other lepidopteran genomes are highly relevant. Karyotypic analysis, presented here alongside the assembly, will be a useful point of reference for future scaffolding efforts. Finally, the authors demonstrate an application of the genome with a preliminary survey and population genomics analysis of 5 populations sampled across Europe and are appropriately cautious in the conclusions they draw from this analysis.

The work described here thoughtfully presents an accomplished assembly, with an approach that should be of broad interest, constitutes an important resource for lepidopteran biology and which anticipates the movement of the genome assembly field towards full diploid reconstruction. I have only minor comments and suggestions, which are set out below.

General

The resolution of the figures in the main submission, but not the supplement, is a little poor in the review copy.

Background

While I agree that full diploid reconstruction is/should be a eukaryotic genome assembly target and that there are few published examples, it might be worth also noting that the Vertebrate Genome Project contains, I believe, 12 trio-based assemblies that are publicly accessible.

Methods

Please confirm that you have not done any of the following (and if you have, please incorporate details in the methods)

Any additional quality trimming of RNAseq reads beyond adaptor removal with cutadapt?

Any pre-processing of PacBio reads to remove adaptor contamination etc?

Please consider also calculating and reporting QV to provide an estimate of assembly accuracy (presenting figures before and after polishing with the 10x reads would be of interest).

Can you elaborate further on the types of artefact/contamination/manual curation that was flagged by

gEval?
I am interested to know more about how you constructed the plots in supplementary figure 1 (e.g. is this from custom parsing of reads lengths/counts in R or a direct visualisation of output from the assembler?). I ask with the vague hope that such qc descriptions might eventually become standardised so that direct comparisons of such metrics between assemblies might become straightforward.
The treatment of the population samples (extraction and sequencing) is the same as for the parental short read sequencing. You could refer back to the earlier description here to avoid repetition.
For clarity, perhaps elaborate briefly on the samples/tissue types within the published RNAseq dataset you use for annotation
Discussion
Prompted by your statement "Successful haplotype separation was possible due to the high estimated heterozygosity…", it might be interesting to explore further how relevant the degree of heterozygosity really is to the success of this approach. Your statement is certainly right for highly fragmented assemblies but with long contigs, it is my sense that even a substantially lower degree of heterozygosity can still give strong support to contig origin and thus fully resolve the haplotypes.
Please consider including some mention of how obtaining appropriate trio samples may be a challenge in non-traditional model systems.
It is probably beyond the scope of this manuscript to touch on possible extensions of this approach to polyploid situations, but potentially this could be raised in the discussion.
Rather than "top tier" perhaps consider using "platinum quality", which seems to be gaining increasing use as a descriptor for assemblies with full chromosome scaffolds and haplotypes resolved across the entire genome.

Level of Interest

Please indicate how interesting you found the manuscript: Choose an item.

Quality of Written English

Please indicate the quality of language in the manuscript: Choose an item.

Declaration of Competing Interests

Please complete a declaration of competing interests, considering the following questions:

- Have you in the past five years received reimbursements, fees, funding, or salary from an organisation that may in any way gain or lose financially from the publication of this manuscript, either now or in the future?
- Do you hold any stocks or shares in an organisation that may in any way gain or lose financially from the publication of this manuscript, either now or in the future?

- Do you hold or are you currently applying for any patents relating to the content of the manuscript?
- Have you received reimbursements, fees, funding, or salary from an organization that holds or has applied for patents relating to the content of the manuscript?
- Do you have any other financial competing interests?
- Do you have any non-financial competing interests in relation to this paper?

If you can answer no to all of the above, write 'I declare that I have no competing interests' below. If your reply is yes to any, please give details below.

I declare that I have no competing interests

I agree to the open peer review policy of the journal. I understand that my name will be included on my report to the authors and, if the manuscript is accepted for publication, my named report including any attachments I upload will be posted on the website along with the authors' responses. I agree for my report to be made available under an Open Access Creative Commons CC-BY license (http://creativecommons.org/licenses/by/4.0/). I understand that any comments which I do not wish to be included in my named report can be included as confidential comments to the editors, which will not be published.

Choose an item.

To further support our reviewers, we have joined with Publons, where you can gain additional credit to further highlight your hard work (see: https://publons.com/journal/530/gigascience). On publication of this paper, your review will be automatically added to Publons, you can then choose whether or not to claim your Publons credit. I understand this statement.

Yes Choose an item.
