# Supplementary material for: A haplotype-resolved, de novo genome assembly for the wood tiger moth (Arctia plantaginis) through trio binning
Source: Gigascience. 2020 Aug 18;9(8):giaa088. doi: 10.1093/gigascience/giaa088 (PMC7433188; doi:10.1093/gigascience/giaa088)
Supplement: giaa088_Reviewer_2_Report_Original_Submission — Shanlin Liu -- 4/12/2020 Reviewed [file giaa088_reviewer_2_report_original_submission.pdf]

Reviewer Report

Title: A haplotype-resolved, de novo genome assembly for the wood tiger moth (Arctia plantaginis) through trio binning

Version: Original Submission **Date:** 4/12/2020

Reviewer name: Shanlin Liu

Reviewer Comments to Author:

The manuscript presents a haplotype-resolved genome assembly for the wood tiger moth that possesses a high-level whole genome heterozygosity level. The manuscript was well written and all the materials and methods were presented in a clear and organized manner. Importantly, it will contribute the studies on entomological genomics and meets the scope of GigaScience. I recommend it be accepted for publication after addressing several minor issues as follows:
1. the authors may want to explain more on the results from the KAT (Kmer based) analysis. For example, how did you obtain the initial Kmer set, from your assemblies or the shotgun reads? If you distinguished single-copy and multiple copy Kmers by tallying their occurrence number in the parental and maternal genomes, how did you define those 0-copy Kmer?
In addition, what is the proportion of your Kmer set that was utilized in the KAT analysis comparing to the entire Kmer set which can be obtained from the genome assembly or the shotgun reads. Will enlarge the K value help to increase the proportion and in turn, increase the power of the analysis?
2. the authors claim a whole genome heterozygosity level of 1.9% for the wood tiger moth, which, however, is estimated using a Kmer based method before obtaining the genome assembly. As you have already obtained the high-quality genome assembly, you may want to re-calculate it, and also it will be great to show readers that how the heterozygous sites distribute on the genome and briefly categorize them according to their types, e.g. SNPs, small InDels and large structure variances(SVs). Validating and visualizing those heterozygous sites makes the quality assessment part more complete.
3. the authors may want to give the unbinned data based assembly a more integrity process, so that makes a fair comparison. For example, you did not apply the 10X data to further scaffold the assembly, or maybe you have but I missed it. You'd better clarify it somewhere in your manuscript.

Level of Interest

Please indicate how interesting you found the manuscript: Choose an item.

Quality of Written English

Please indicate the quality of language in the manuscript: Choose an item.

Declaration of Competing Interests

Please complete a declaration of competing interests, considering the following questions:

- Have you in the past five years received reimbursements, fees, funding, or salary from an organisation that may in any way gain or lose financially from the publication of this manuscript, either now or in the future?
- Do you hold any stocks or shares in an organisation that may in any way gain or lose financially from the publication of this manuscript, either now or in the future?
- Do you hold or are you currently applying for any patents relating to the content of the manuscript?
- Have you received reimbursements, fees, funding, or salary from an organization that holds or has applied for patents relating to the content of the manuscript?
- Do you have any other financial competing interests?
- Do you have any non-financial competing interests in relation to this paper?

If you can answer no to all of the above, write 'I declare that I have no competing interests' below. If your reply is yes to any, please give details below.

I declare that I have no competing interests

I agree to the open peer review policy of the journal. I understand that my name will be included on my report to the authors and, if the manuscript is accepted for publication, my named report including any attachments I upload will be posted on the website along with the authors' responses. I agree for my report to be made available under an Open Access Creative Commons CC-BY license (http://creativecommons.org/licenses/by/4.0/). I understand that any comments which I do not wish to be included in my named report can be included as confidential comments to the editors, which will not be published.

Choose an item.

To further support our reviewers, we have joined with Publons, where you can gain additional credit to further highlight your hard work (see: https://publons.com/journal/530/gigascience). On publication of this paper, your review will be automatically added to Publons, you can then choose whether or not to claim your Publons credit. I understand this statement.

Yes Choose an item.
